# KLF17 promotes human naïve pluripotency but is not required for its establishment

**DOI:** 10.1242/dev.199378

**Published:** 2021-11-15

**Authors:** Rebecca A. Lea, Afshan McCarthy, Stefan Boeing, Todd Fallesen, Kay Elder, Phil Snell, Leila Christie, Sarah Adkins, Valerie Shaikly, Mohamed Taranissi, Kathy K. Niakan

**Affiliations:** 1 Human Embryo and Stem Cell Laboratory, The Francis Crick Institute, 1 Midland Road, London NW1 1AT, UK; 2 Bioinformatics and Biostatistics Service, The Francis Crick Institute, 1 Midland Road, London NW1 1AT, UK; 3 Crick Advanced Light Microscopy, The Francis Crick Institute, 1 Midland Road, London NW1 1AT, UK; 4 Bourn Hall Clinic, Bourn, Cambridge CB23 2TN, UK; 5 Assisted Reproduction and Gynaecology Centre, London W1G 6LP, UK; 6 The Centre for Trophoblast Research, Department of Physiology, Development and Neuroscience, University of Cambridge, Cambridge CB2 3EG, UK

**Keywords:** Human embryonic stem cells, Naïve pluripotency, Epiblast, KLF17, KLF factors

## Abstract

Current knowledge of the transcriptional regulation of human pluripotency is incomplete, with lack of interspecies conservation observed. Single-cell transcriptomics analysis of human embryos previously enabled us to identify transcription factors, including the zinc-finger protein KLF17, that are enriched in the human epiblast and naïve human embryonic stem cells (hESCs). Here, we show that KLF17 is expressed coincident with the known pluripotency-associated factors NANOG and SOX2 across human blastocyst development. We investigate the function of KLF17 using primed and naïve hESCs for gain- and loss-of-function analyses. We find that ectopic expression of KLF17 in primed hESCs is sufficient to induce a naïve-like transcriptome and that KLF17 can drive transgene-mediated resetting to naïve pluripotency. This implies a role for KLF17 in establishing naïve pluripotency. However, CRISPR-Cas9-mediated knockout studies reveal that KLF17 is not required for naïve pluripotency acquisition *in vitro*. Transcriptome analysis of naïve hESCs identifies subtle effects on metabolism and signalling pathways following KLF17 loss of function, and possible redundancy with other KLF paralogues. Overall, we show that KLF17 is sufficient, but not necessary, for naïve pluripotency under the given *in vitro* conditions.

## Abstract (precis)

**Summary:** Given that KLF17 was shown to be sufficient, but not necessary, to establish naïve pluripotent hESCs, KLF17 might function as a peripheral regulator of human pluripotent stem cells.

## INTRODUCTION

Model organisms, such as the mouse, have allowed molecular mechanisms that regulate early mammalian development to be identified (Rossant, 2016), some of which are conserved in humans (Gerri et al., 2020). Despite the continued importance of comparative studies in mouse and other organisms, some aspects of early development, such as developmental timing, chromatin accessibility and transcription factor function, are distinct compared with humans (Niakan and Eggan, 2013; Fogarty et al., 2017; Gao et al., 2018). In particular, the advent of single-cell sequencing technologies has allowed in-depth transcriptomic analysis of human embryos, revealing a number of molecular differences compared with the mouse (Yan et al., 2013; Blakeley et al., 2015; Petropoulos et al., 2016; Stirparo et al., 2018). Our previous analysis highlighted that several genes thought of as canonical pluripotency-associated factors in the mouse, including *KLF2*, *ESRRB* and *BMP4* (Blakeley et al., 2015), are not expressed in the pluripotent epiblast (EPI) of the human preimplantation embryo, which forms the embryo itself. Conversely, we also highlighted genes that are specifically enriched in the human EPI, but not expressed in the pluripotent cells of the mouse embryo, including transcriptional regulators and signalling components (Blakeley et al., 2015).

The gene encoding the zinc-finger DNA-binding protein KLF17 is one of these human EPI-enriched genes. KLF17 belongs to the Krüppel-like transcription factor family involved in development, which includes KLF4, a commonly used reprogramming factor (Takahashi and Yamanaka, 2006), and KLF2, a known pluripotency regulator in the mouse (Hall et al., 2009). Given the lack of *KLF2* expression in the human EPI, it is interesting to speculate that KLF17 might function in a similar way. Indeed, the expression patterns of *KLF2* and *KLF17* in the human embryo are diametrically opposite to those of *Klf2* and *Klf17* in the mouse embryo (Yan et al., 2013; Blakeley et al., 2015). Whereas *Klf17* appears to be maternally deposited in the mouse zygote and its expression is abolished around the eight-cell stage, *KLF17* is dramatically upregulated in the eight-cell human embryo, following embryonic genome activation (EGA) (Yan et al., 2013; Deng et al., 2014; Blakeley et al., 2015). Conversely, *Klf2* is expressed from the two-cell stage, corresponding to mouse EGA, and continues through to the blastocyst stage, whereas human *KLF2* is only expressed pre-EGA (Yan et al., 2013; Deng et al., 2014; Blakeley et al., 2015). The human KLF17 and KLF2 sequences share ∼60% homology across the C-terminal region containing the functional C_2_H_2_-type zinc-finger domains. KLF17 and mouse KLF2 also have additional homologous regions (∼50%) throughout the protein, including part of a region in mouse KLF2 annotated as a protein-protein interaction domain, which may contribute to regulation and/or functional specificity. Furthermore, in mouse embryonic stem cells (mESCs), the triple knockout of *Klf2*, *Klf4* and *Klf5* can be rescued by ectopic expression of human *KLF17* or mouse *Klf17* (Yamane et al., 2018). Finally, the human and mouse KLF17 protein sequences have less similarity overall compared with other pairs of KLF orthologues (van Vliet et al., 2006). This is all suggestive of rapid, divergent evolution of the human and mouse KLF genes and a potential switching of their function between species.

To date, KLF17 has been studied primarily in the context of cancer, in which it has been implicated as a tumour suppressor by its interaction with TGFβ/SMAD signalling (Ali et al., 2015b) and p53 (Ali et al., 2015a), and its inhibition of epithelial-to-mesenchymal transition (Gumireddy et al., 2009; Zhou et al., 2016). Since the recognition of its human EPI-specific expression, KLF17 has been widely used as a marker of pluripotency in the human embryo (Blakeley et al., 2015; Guo et al., 2016; Shahbazi et al., 2017; Kilens et al., 2018). The expression of *KLF17* throughout preimplantation development, and in particular in pluripotent cells, is also conserved in a number of other organisms, including nonhuman primates [rhesus monkey, *Macaca mulatta* (Wang et al., 2017); common marmoset, *Callithrix jacchus* (Boroviak et al., 2015); and cynomolgus monkey, *Macaca fascicularis* (Nakamura et al., 2016)] and pig, *Sus scrofa* (Bernardo et al., 2018; Ramos-Ibeas et al., 2019). Intriguingly, KLF17 expression is not detectable in conventionally derived ‘primed’ human ESCs (hESCs) (Blakeley et al., 2015; Stirparo et al., 2018), reflecting their post-implantation-like identity. However, newer methods for deriving and/or culturing hESCs and human induced pluripotent stem cells (hiPSCs) in a naïve pluripotent state result in the maintenance or reinstatement of *KLF17* activity (Theunissen et al., 2014; Guo et al., 2017, 2016; Liu et al., 2017; Kilens et al., 2018). This pattern of expression suggests the intriguing possibility that KLF17 acts as a transcriptional regulator of human naïve pluripotency, as exhibited in the bona fide state of the preimplantation EPI and approximated in *in* *vitro* naïve hESC models. This hypothesis has also been explored by independent transcriptome analysis (Stirparo et al., 2018).

Studies to date have conclusively shown only that KLF17 is a marker of human pluripotency. Here, we aimed to determine the function of KLF17, finding that its induced expression in conventional hESCs is sufficient, alongside naïve-permissive pluripotency conditions, to induce a complete change in phenotype from primed to naïve pluripotency. However, we also found that the null mutation of *KLF17* in conventional hESCs is not detrimental to naïve resetting or maintenance of the resulting naïve cells. Taken together, these results suggest that KLF17 functions to regulate genes associated with human naïve pluripotency, but that there is a degree of redundancy *in vitro*, such that KLF17 itself is not strictly necessary for the acquisition and maintenance of naïve pluripotency.

## RESULTS

### KLF17 expression in the human embryo is gradually restricted to the epiblast

**Fig. 1.**
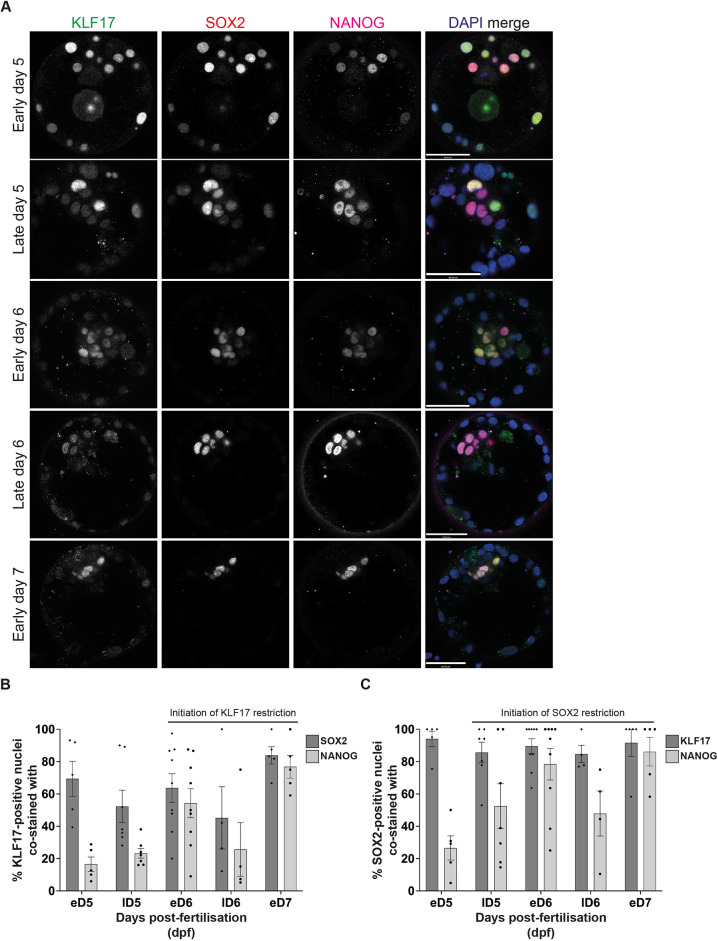
**KLF17 expression in the human embryo is coincident with known pluripotency factors.** (A) Representative images of IF analysis of blastocyst-stage human embryos at early day 5 (*n*=5), late day 5 (*n*=7), early day 6 (*n*=9), late day 6 (*n*=4) and early day 7 (*n*=5) post-fertilisation. (B,C) Proportion of (B) KLF17-positive nuclei per embryo that are SOX2 positive or NANOG positive and (C) SOX2-positive nuclei per embryo that are KLF17 positive or NANOG positive. Bars represent the mean, error bars the s.e.m., and black dots or diamonds the percentage overlap in individual embryos. Scale bars: 50 μm in A.

Detailed single-cell RNA-sequencing (scRNA-seq) studies highlight *KLF17* as a molecular marker that is expressed in the human preimplantation EPI. First, we reassessed the protein expression dynamics of KLF17 in human embryos to investigate its distribution across the developing blastocyst. We performed immunofluorescence (IF) analysis of KLF17 alongside the canonical pluripotency factors SOX2 and NANOG in human embryos from the early to late blastocyst stage (Fig. 1A; Fig. S1). NANOG is the earliest-known EPI-restricted factor in human embryos (Kimber et al., 2008; Niakan and Eggan, 2013), whereas *SOX2* expression dynamics closely resemble those of *KLF17* (Blakeley et al., 2015). In keeping with previous data (Kilens et al., 2018), we found that, during the earliest stage examined [early day 5 post-fertilisation (dpf)], KLF17 protein was detectable in the majority of cells of the embryo, with KLF17 expression in an average of 64% of all nuclei (Fig. 1; Fig. S1). Although the expression levels across all nuclei were heterogeneous, this widespread staining of KLF17 largely coincided with SOX2 in both the inner cell mass (ICM) and trophoectoderm (TE) populations at this stage (Fig. 1; Fig. S1). Indeed, an average of 70% of KLF17-positive cells at early day 5 dpf also expressed SOX2, compared with only a 16.5% overlap with NANOG (Fig. 1B). As blastocyst development progressed, KLF17 expression was gradually restricted, with ICM enrichment by early day 6 dpf (Fig. 1; Fig. S1), as evidenced by an average overlap with SOX2 of 64% and with NANOG of 54% (Fig. 1B). In early day 7 dpf blastocysts, KLF17 was restricted to the presumptive EPI cells, delineated by nearly exclusive co-staining with both SOX2 and NANOG (Fig. 1; Fig. S1). Interestingly, the restriction of KLF17 appeared to progress more slowly than that of SOX2. By late day 5 dpf, SOX2 was only appreciably expressed in the ICM and, to a lesser extent, in polar TE (mean 52% overlap with NANOG) and it was restricted to the NANOG-positive EPI in early day 6 dpf embryos (mean 78% overlap with NANOG) (Fig. 1C). In contrast, there remained appreciable KLF17 protein across cells of the TE in most of the late day 6 dpf blastocysts analysed (Fig. 1; Fig. S1). This suggests that the half-life of KLF17 protein may be longer than that of SOX2, given the absence of *KLF17* transcripts in the extra-embryonic lineages of human blastocysts by scRNA-seq analysis (Blakeley et al., 2015). As reported previously, NANOG was detected in relatively few cells at all stages of blastocyst development (Niakan and Eggan, 2013) (Fig. S1C). These NANOG-positive cells represent the preimplantation EPI. Despite the initial widespread expression pattern of KLF17, its gradual restriction to the NANOG/SOX2 dual-positive EPI suggests that it is specifically retained in the pluripotent compartment, perhaps to perform an unappreciated role in pluripotency regulation or EPI development.

### Induction of KLF17 promotes a naïve pluripotency-like phenotype in conventional hESCs

Given that KLF17 is not expressed in conventional primed hESCs, we investigated the effect of ectopic overexpression of *KLF17* in these conditions. We hypothesised that KLF17, as a transcriptional regulator that is enriched in the naïve state (Blakeley et al., 2015; Guo et al., 2016; Messmer et al., 2019), might be sufficient to regulate other naïve pluripotency-associated genes when ectopically expressed in primed pluripotent hESCs.

**Fig. 2.**
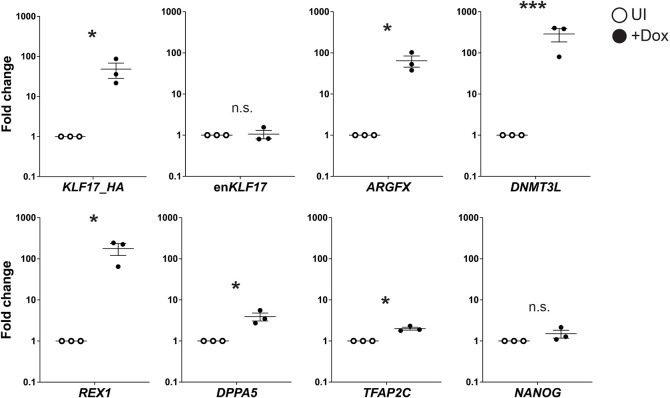
**Exogenous KLF17 overexpression induces naïve factor expression in conventional hESCs.** qRT-PCR analysis of H9 KLF17-HA-inducible hESCs following 5 days with (+Dox) or without (UI) Dox induction of exogenous KLF17. Relative expression is displayed as the fold change versus UI cells and normalised to *GAPDH* as a housekeeping gene using the ΔΔCt method. Individual samples are shown as filled or unfilled dots, horizontal lines represent the mean and whiskers the s.e.m. Data analysed with Welch's *t*-test; ***P<0.005; *P<0.05; n.s., not significant; *n*=3.

We generated hESCs with doxycycline (Dox)-inducible, 3′ HA-tagged *KLF17* transgene expression (Fig. S2A) and found that 5 days of treatment with 1 μg/ml Dox was sufficient for robust expression of KLF17 (Fig. S2B). Therefore, we examined the possibility of gene expression changes in response to ectopic KLF17 in primed culture conditions. Using quantitative RT-PCR (qRT-PCR), we analysed the expression of a number of genes identified as either naïve or primed enriched through previous differential gene expression analyses (Stirparo et al., 2018; Messmer et al., 2019) after 5 days of Dox induction (Fig. 2). We identified naïve-enriched factors that were significantly upregulated in response to *KLF17* induction: *ARGFX* (∼65-fold; *P*=0.03), *ZFP42* (∼180-fold; *P*=0.02), *DPPA5* (∼3.9-fold; *P*=0.04), *DNMT3L* (∼300-fold; *P*=0.003) and *TFAP2C* (∼2-fold; *P*=0.03). Of these genes, our recent scRNA-seq analysis revealed that only *ZFP42* is appreciably expressed in primed hESCs (Wamaitha et al., 2020). Therefore, expression of *KLF17* alone is sufficient not only to upregulate a gene already active in conventional hESCs, but also to initiate the expression of genes that are otherwise transcriptionally silent. By contrast, expression of *NANOG* and endogenous *KLF17* remained unchanged, revealing a lack of KLF17 autoregulation (Fig. 2).

**Fig. 3.**
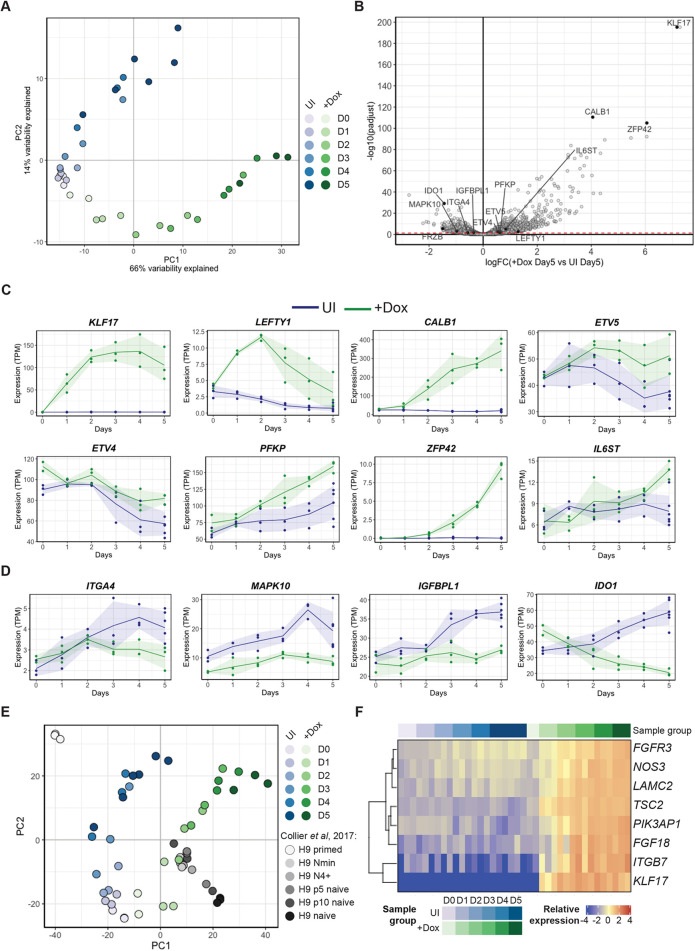
**Exogenous KLF17 overexpression induces widespread transcriptional change in conventional hESCs.** (A) Dimensionality reduction by PCA of bulk RNA-seq data collected across a 5-day time course (D0-D5) of H9 KLF17-HA-inducible hESC growth with (+Dox) or without (UI) Dox induction of exogenous KLF17 expression. (B) Volcano plot displaying relative expression of all genes detected in +Dox versus UI H9 KLF17-HA hESCs at day 5 [logFC(+Dox Day5 versus UI Day5)] against the significance of differential expression [-log10(padjust)]. The red-dashed line indicates *P*_adj_=0.05. Individual genes of interest are displayed as black-filled circles and labelled with the gene name. (C,D) Normalised expression (transcripts per million; TPM) of individual genes of interest across the 5-day time course in UI and KLF17-expressing (+Dox) H9 KLF17-HA hESCs, showing genes that were significantly upregulated (C) or downregulated (D) at day 5. Solid lines show the mean value and shading shows the mean±s.d. (E) Batch-corrected PCA analysis of the data from A integrated with published bulk RNA-seq data of samples collected before (‘H9 primed’), during (‘H9 Nmin’ and ‘H9 N4+’) and following (‘H9 p5 naïve’, ‘H9 p10 naïve’ and ‘H9 naïve’) *NANOG* and *KLF2*-driven resetting of H9 hESCs (Collier et al., 2017). (F) Heatmap grouped by sample (UI or +Dox) and time point showing the genes that were highly correlated with *KLF17* across time [Pearson correlation coefficient (r) ≥0.85] and that fall under the Kyoto Encyclopedia of Genes and Genomes category ‘PI3K-Akt signalling pathway’.

To understand the full extent of the gene expression changes following KLF17 overexpression, we performed mRNA-seq across a 5-day time course of induction. Dimensionality reduction by principal component analysis (PCA) separated the samples by treatment [uninduced (UI) or induced (+Dox)] and timepoint (Fig. 3A). The UI control cells progressed through PC2 with time, reflecting transcriptional changes that occur following passaging. However, even as early as day 2, the induced and UI hESCs were clearly separated by PC1 and PC2 (Fig. 3A). Thus, ectopic expression of KLF17 in primed hESCs was sufficient to rapidly bring about considerable transcriptome-wide changes.

To determine the nature of the genes impacted by KLF17, we performed differential gene expression analysis between UI and induced samples at each timepoint. At day 5, we uncovered 1760 and 1315 up- and downregulated genes, respectively (*P*_adj_<0.05) (Fig. 3B; Table S1). Of the upregulated genes at day 5, 537 (31%) have been previously identified as being enriched in naïve hESCs (Stirparo et al., 2018; Messmer et al., 2019) and/or the human EPI (Blakeley et al., 2015), including 46 genes that are EPI enriched but not differentially expressed between naïve and primed hESCs (e.g. *LEFTY1*, *CALB1*, *ETV5*, *ETV4* and *PFKP*) (Fig. 3C). In contrast, of the downregulated genes, 479 (36%) were previously identified as exclusively enriched in primed hESCs (e.g. *ITGA2*, *ITGA4*, *MAPK10*, *IGFBPL1* and *IDO1*) (Fig. 3D). This suggests that expression of KLF17 in hESCs cultured under primed culture conditions promotes a shift toward a more-naïve pluripotent transcriptome. Indeed, this was supported by the observation that cells following 1 or 2 days of Dox induction began to cluster transcriptionally with bona fide resetting intermediates identified at day 10 during resetting driven by *NANOG* and *KLF2* (*NK2*) expression (Collier et al., 2017) (Fig. 3E).

Thus, it appears that KLF17 alone is sufficient to induce significant transcriptional changes in primed hESCs over 5 days. To identify those genes most likely to be regulated directly by KLF17, we performed a time course correlation analysis. Using a cut-off for the correlation coefficient of 0.85, we found 70 genes the expression of which over time closely mimicked that of exogenous *KLF17* (Fig. S3A; Table S2). Of these genes, two-thirds (47) were classified as significantly enriched from day 1 (*P*_adj_<0.05) and almost all (69) were classified as significantly enriched from day 2 onwards (*P*_adj_<0.05) (Fig. S3B,C), highlighting that these putative KLF17 targets were both rapidly and strongly upregulated following *KLF17* induction. These genes included a number of components of the PI3K-AKT-mTOR signalling pathway (*PIK3AP1*, *TSC2*, *NOS3*, *FGF18*, *FGFR3*, *ITGB7* and *LAMC2*; Fig. 3F), which is active in both primed and naïve hESCs and a driver of primed hESC and human EPI proliferation (Wamaitha et al., 2020). Following *KLF17* overexpression, hESCs significantly upregulated ligands, receptors and downstream components of the PI3K-AKT pathway (Fig. S4A-G), which are also enriched in the human EPI (Wamaitha et al., 2020). PI3K-AKT-mTOR signalling has also been implicated in an alternative state of naïve pluripotency (Duggal et al., 2015). This suggests that KLF17 induction may modulate signalling through PI3K to a more-naïve or EPI-like state.

To determine the activation state of the PI3K-AKT signalling pathway in UI and induced hESCs, we performed western blot analysis of the key players (Fig. S5). We found that phosphorylation of AKT was consistently decreased in response to KLF17 expression at day 5 (Fig. S5C,D), as was phosphorylation of the more-downstream effector, S6 (Fig. S5E). We also observed a decrease in phosphorylation of the upstream receptors, IGF1R and InsR (Fig. S5F), whereas phosphorylation of ERK1/2, which can crosstalk with AKT (Lamothe et al., 2004; Ornitz and Itoh, 2015), was consistently increased (Fig. S5G). The active, phosphorylated form of S6 is known to induce negative feedback at the level of upstream receptors (Harrington et al., 2004; Tremblay et al., 2007; Carracedo and Pandolfi, 2008). Meanwhile, pAKT(Serine473) is usually mediated by the activity of the downstream effector mTOR and stimulates full AKT activity (Alessi et al., 1996; Sarbassov et al., 2005), thereby regulating functions including metabolism, growth and proliferation. Therefore, a reduction in the phosphorylation levels at various points in the pathway following upregulation of genes associated with PI3K-AKT-mTOR signalling may indicate negative feedback, acting to keep the *KLF17*-induced hESCs in a steady state.

Other signalling factors were also highly correlated with *KLF17*, including *JAKMIP2*, *FGFRL1* and *TNFRSF8* and the TGFβ signalling pathway components *LEFTY2* and *TGFB1I1*. Several cell adhesion-related and cytoskeletal proteins were also included in this list: *LAMC2*, *MUC4*, *COL5A1*, *ITGB7* and *MXRA5* (Fig. S4G-O). Given that changes in morphology and signalling are hallmarks of the conversion of primed to naïve pluripotency, it appears that KLF17 induces some of the same resetting-associated changes without any external signalling modulation.

Of note is the strong correlation between *KLF17* transgene expression and that of the long noncoding RNA *LINC*-*ROR* (r=0.921), which was upregulated ∼2.4-fold after 24 h induction (Fig. S4P). *LINC*-*ROR* has been identified as a regulator of iPSC reprogramming (Loewer et al., 2010) and hESC self-renewal (Wang et al., 2013). Its expression is regulated by the core pluripotency transcription factors OCT4, SOX2 and NANOG (Loewer et al., 2010) and, in turn, it acts as a sink for pluripotency destabilising miRNAs that target the mRNA of these core factors for degradation (Wang et al., 2013). Thus, through increased *LINC-ROR* expression, ectopic *KLF17* may limit hESC differentiation.

Finally, we noted that terms related to WNT signalling were enriched among the 1711 genes downregulated after 24 h of Dox induction (Table S3). Activity of the WNT pathway has been suggested to promote differentiation of hESCs in both primed and naïve pluripotent states (Davidson et al., 2012; Singh et al., 2012; Bredenkamp et al., 2019b) and to be suppressed through crosstalk with the PI3K-AKT signalling pathway (Singh et al., 2012). Therefore, downregulation of genes associated with WNT signalling may suggest a mechanism through which KLF17-overexpressing hESCs would be refractory to differentiation cues.

Of the 50 WNT signalling-associated genes identified as significantly downregulated following 24 h of *KLF17* expression, 74% (37) have been suggested as sites of KLF17 binding under alternative naïve hESC culture conditions (Bayerl et al., 2021), including *GSK3B*, *TNKS2* and *CTNNB1* (Fig. S6A-C). Similarly, of the 70 genes the expression dynamics of which closely mimicked the *KLF17* transgene, 63% (44) were identified as putative sites of KLF17 binding (Bayerl et al., 2021), including *LAMC2* and *FGF18* (Fig. S6D,E). Despite the difference in culture conditions, this could suggest that KLF17 directly regulates the expression of these genes.

We confirmed the expression patterns of a number of differentially expressed genes (DEGs) by qRT-PCR (Fig. S6F-K) and/or IF analysis (Fig. S6L), including *DNMT3L*, *VENTX*, *GP130* and *TFAP2C*, the latter of which is an essential regulator of naïve hESCs (Pastor et al., 2018). Altogether, this supports our hypothesis that KLF17 acts to regulate transcriptionally genes associated with naïve human pluripotency.

### KLF17 expression drives hESCs to naïve pluripotency alongside signalling modulation

Given that KLF17 is sufficient to upregulate naïve pluripotency-associated factors under conventional primed hESC conditions, we hypothesised that *KLF17* induction may be sufficient to reset primed hESCs to a naïve pluripotent state under the appropriate culture regime. The use of ectopic gene expression to drive resetting is common, with deployment of transgenes, including *OCT4*, *KLF4*, *SOX2*, *YAP*, *NANOG* and/or *KLF2* (Hanna et al., 2010; Theunissen et al., 2014; Takashima et al., 2014; Qin et al., 2016; Liu et al., 2017), with various media compositions.

**Fig. 4.**
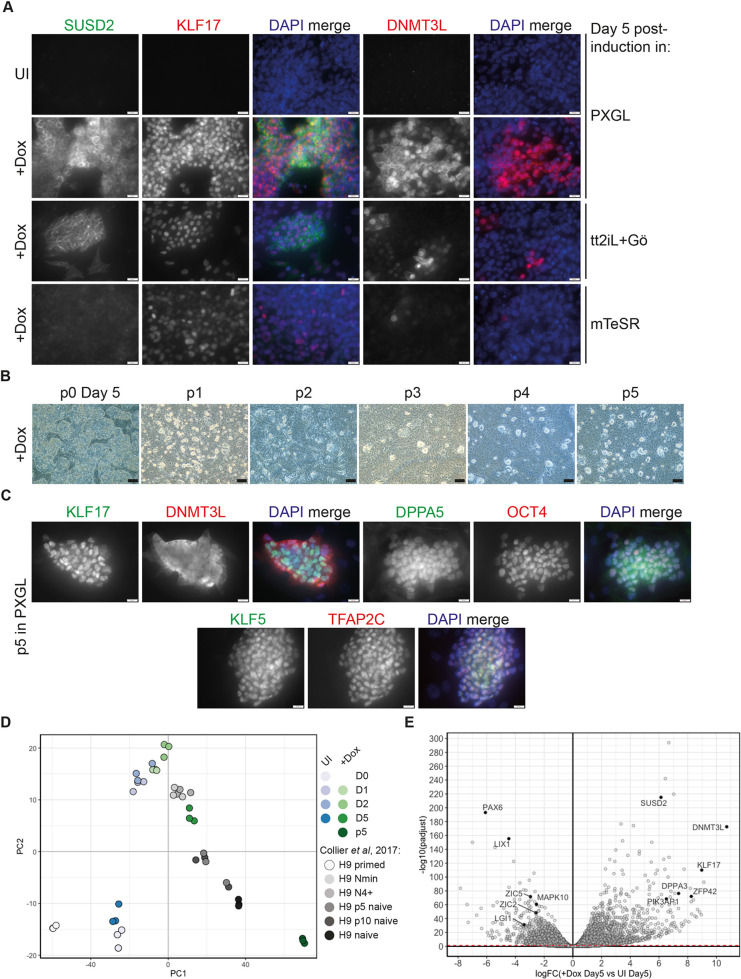
**Exogenous KLF17 overexpression is sufficient to drive conventional hESCs to a naïve pluripotent state under PXGL culture conditions.** (A) IF analysis of H9 KLF17-HA-inducible hESCs following 5 days UI or 5 days of Dox induction (+Dox) in the indicated media. Cells were cultured on a MEF feeder layer and at 5% O_2_; *n* ≥3. (B) Cells induced for 5 days in PXGL medium were uniquely able to give rise to typical naïve hESC-like colonies following serial bulk passaging. (C) Representative IF analysis of H9 KLF17-HA-induced naïve hESCs after four or five passages in PXGL medium; *n* ≥3. (D) Batch-corrected PCA analysis of bulk RNA-seq data tracking the progress of KLF17-driven resetting (this study) and *NK2*-driven resetting (Collier et al., 2017). (E) Volcano plot displaying relative expression of all genes detected in +Dox versus UI H9 KLF17-HA hESCs at day 5 [logFC(+Dox Day5 versus UI Day5)] of culture in PXGL against the significance of differential expression [-log10(padjust)]. The red-dashed line indicates *P*_adj_=0.05. Individual genes of interest are displayed as black-filled circles and labelled with the gene name. Scale bars: 20 μm in A,C; 200 μm in B.

Initial testing of *KLF17* induction for 5 days under two naïve hESC culture conditions, tt2iL+Gö (Guo et al., 2017) and PXGL (Bredenkamp et al., 2019a,b), revealed considerably stronger expression of the naïve markers DNMT3L and SUSD2 compared with cells treated equivalently in conventional (mTeSR1) medium or untreated controls (Fig. 4A; Fig. S7A). The upregulation of SUSD2 expression is particularly noteworthy, because it has been recently identified as a highly specific cell surface marker of naïve hESCs (Bredenkamp et al., 2019a). We attempted to propagate the cells, by single-cell passaging, in both naïve and primed conditions after 5 days of *KLF17* induction. Rounded and highly refractile colonies showing typical naïve hESC morphology began to appear only in cells treated with Dox in PXGL medium (Fig. 4B). Conversely, most of the UI cells in PXGL had died (Fig. S7B). Whereas the survival of UI cells was equally compromised following passaging in tt2iL+Gö, the induced cells showed evidence of differentiation only, whereas all cells grown in mTeSR1 survived with typical primed hESC morphology (Fig. S7B). This suggests that the ectopic expression of *KLF17* is sufficient to reset conventional hESCs to a naïve-like pluripotent phenotype when supported by PXGL medium (Guo et al., 2017). During chemical epigenetic resetting, PXGL medium supports the initial primed-to-naïve transition via WNT signalling modulation through XAV939 (Guo et al., 2017). This suggests that WNT inhibition is important alongside *KLF17* overexpression for primed-to-naïve resetting.

Bulk, single-cell passaging of these naïve-like colonies allowed for stable propagation of *KLF17*-inducible naïve hESCs for a minimum of five passages in PXGL, without requiring additional transgene activation beyond the initial 5-day period of Dox treatment (Fig. 4B). We were able to confirm protein expression of naïve hESC markers, and of factors identified above as upregulated following *KLF17* induction in primed culture conditions, although we also found that DNMT3L was no longer entirely nuclear in PXGL, perhaps because of the substantial increase in its level of expression (Fig. 4C).

Furthermore, we performed bulk mRNA-sequencing of cells undergoing *KLF17*-driven resetting (+Dox on days 1, 2 and 5), alongside day 0 and UI controls and KLF17-inducible naïve cells at passage 5 (p5) in PXGL. As a comparison, we again incorporated the data representing the progression of primed to naïve hESCs driven by *NK2* overexpression in t2iL+Gö conditions (Collier et al., 2017). PCA analysis revealed that, whereas culture in PXGL alone for 48 h caused considerable transcriptome-wide differences (Fig. 4D), UI cells regressed to a more typical hESC transcriptome, as evidenced by clustering of these cells with the day 0 samples from this study and published primed hESCs (Collier et al., 2017) (Fig. 4D). In contrast, hESCs overexpressing exogenous *KLF17* for 5 days in PXGL represented bona fide intermediates between the primed and naïve pluripotent states, given their clustering close to the H9 Nmin and N4+ intermediates of *NK2*-driven resetting (Collier et al., 2017) (Fig. 4D). Continued propagation of these cells to p5 in PXGL induced a similar naïve state to that previously reported (Collier et al., 2017). KLF17-inducible naïve hESCs at p5 were clearly well separated in transcriptional space from conventional primed cells, but did not fall within the cluster of p5, p10 and established naïve hESCs in t2iL+Gö (Collier et al., 2017) (Fig. 4D).

To investigate the transcriptional changes that occur during KLF17-driven resetting in more detail, we performed DEG analysis between the induced and UI cells over time. In keeping with their proximity in the PCA, there were relatively few significant (*P*_adj_<0.05) DEGs at days 1 and 2. However, examination of the transcriptomes of KLF17-inducible hESCs following 5 days of culture in PXGL revealed 5057 genes and 4405 genes significantly up- and downregulated in induced versus UI cells, respectively (Fig. 4E; Table S4). Of the most strongly and significantly enriched genes, we identified a number of factors previously highlighted as naïve-enriched genes (Stirparo et al., 2018; Messmer et al., 2019), including *SUSD2*, *DNMT3L*, *ZFP42* and *DPPA3*, as well as genes previously associated with KLF17 overexpression in primed conditions, such as *PIK3AP1* (Fig. 4E). Conversely, some of the most significantly downregulated genes included a number of primed hESC markers (Stirparo et al., 2018; Messmer et al., 2019), such as *PAX6*, *ZIC2*, *ZIC5*, *LGI1* and *MAPK10* (Fig. 4E).

Examining the dynamics of gene expression in more detail, we manually defined three broad groups of genes: those that were specifically upregulated in response to KLF17 induction (Fig. S8A); those that were restrained or repressed in response to KLF17 induction (Fig. S8B); and those that changed expression in response to the PXGL culture condition alone (Fig. S8C). Whereas culture in PXGL initially led UI hESCs towards a more-naïve intermediate-like state (Fig. 4D; Fig. S8C), the specific transcriptional modulation of a number of factors either directly or indirectly by KLF17 was clearly required to enable primed-to-naïve-like transcriptional conversion (Fig. S8A,B). Interestingly, WNT pathway components were among those genes specifically restrained by KLF17 overexpression (Fig. S8B), reinforcing the notion of WNT signalling regulation via KLF17. Therefore, we demonstrated that KLF17 is a potent inducer of the naïve pluripotent state in hESCs, capable of synergising with the appropriate culture environment to bring about a switch from primed to naïve pluripotency.

### Designing a strategy for KLF17 mutation in hESCs

Next, we sought to determine whether KLF17 expression is required for resetting of primed hESCs to naïve pluripotency. For this, we optimised a protocol for CRISPR-Cas9-mediated mutation of *KLF17*.

**Fig. 5.**
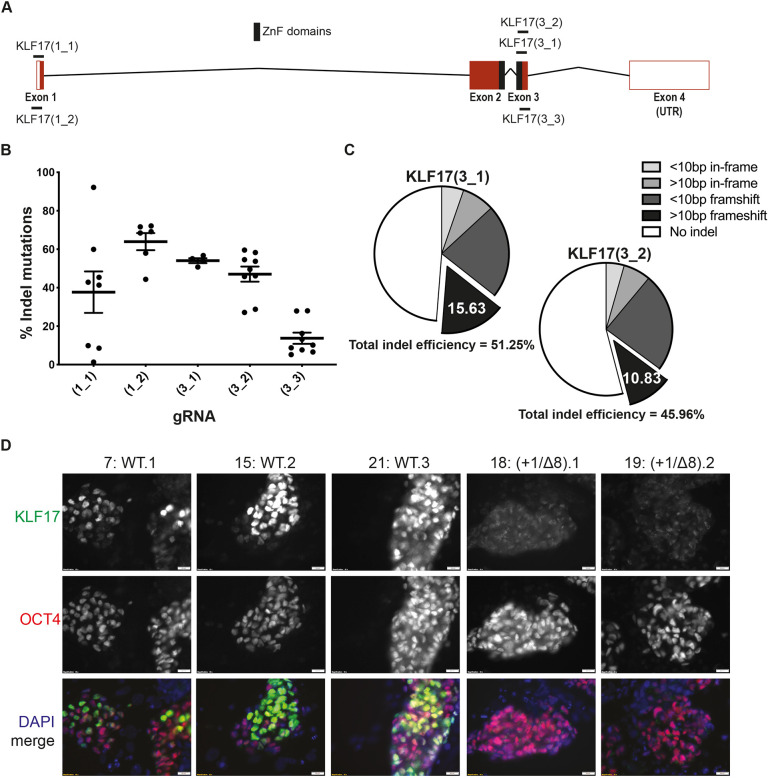
**Generating *KLF17*-null mutant hESCs by CRISPR-Cas9.** (A) Schematic of the human *KLF17* locus on Chromosome 1, showing the relative position of the DNA-binding zinc finger domains (filled-black rectangles) and the gRNAs tested for mutagenic efficiency. Exons are shown as red rectangles, 3′ and 5′-untranslated regions (UTR) are unfilled rectangles and introns are black chevrons. (B) Relative efficiency of each gRNA shown in A measured as a proportion of overall reads containing indel mutations following on-target amplification by MiSeq of the *KLF17* target site. Dots represent individual harvested wells of CRISPR-targeted H9 hESCs, horizontal lines represent the mean and whiskers the s.e.m. (C) Pie charts representing the relative proportions of different outcomes of CRISPR-Cas9 editing of H9 hESCs, based on the sequences detected by MiSeq analysis. (D) IF analysis of H9 hESCs targeted with Cas9 and gRNA KLF17(3_1) following epigenetic resetting (Guo et al., 2017) for 8 days. Internal WT controls (#7, #15 and #21) are clones that were subjected to nucleofection, puromycin selection and clonal expansion, but were unedited, with a WT genotype. Compound null mutant clones (#18 and #19) were verified by MiSeq and IF; *n*=3. Scale bars: 20 μm in D.

Using *in silico* tools, we designed five guide RNAs (gRNAs) against *KLF17* (Fig. 5A; Fig. S9A). We introduced Cas9 and each gRNA in turn into primed hESCs and performed deep sequencing of the *KLF17* on-target locus by MiSeq analysis. This revealed that the introduction of Cas9 and each of the gRNAs led to insertion and deletion (indel) mutations, with an average mutation efficiency of ∼60% (Fig. 5B). However, gRNA KLF17(3_3) was clearly inferior and, therefore, we did not consider it any further.

To decide upon the optimal gRNA for generating *KLF17*-null mutant (*KLF17*^−/−^) hESCs, we investigated the nature of the indels resulting from CRISPR-Cas9 targeting in each case. First, it was clear that both KLF17(1_1) and KLF17(1_2) were biased towards the introduction of very small indel mutations, with the majority of indels less than 10 bp in size (Fig. S9B). Thus, targeting with either of the exon 1-targeted gRNAs would leave the possibility of *KLF17* expression from an identified alternative initiating methionine, with the possibility of generating a hypomorph with unexpected consequences. Therefore, we focused on the exon 3-targeting gRNAs, KLF17(3_1) and KLF17(3_2). The overall efficiency of these two gRNAs was very similar, but sequence analysis revealed a stronger propensity for the introduction of larger frameshift alleles by KLF17(3_1) (Fig. 5C). By disrupting a larger stretch of sequence within the region encoding the KLF17 DNA-binding domain, longer frameshift indels would be expected to lead to null mutations. Therefore, we aimed to generate *KLF17*^−/−^ hESC lines using gRNA KLF17(3_1).

### *KLF17*^−/−^ hESCs are not impaired in their ability to adopt naïve pluripotency

Following nucleofection and single-cell amplification of wild-type (WT), primed hESCs, we generated eight *KLF17*-targeted clones (Fig. S10A). Initial genotyping by short-range PCR and next-generation MiSeq suggested a high proportion of homozygous editing (five of eight edited clones; Fig. S10A,B). However, analysis of a ∼950 bp region surrounding the on-target site revealed that these apparent homozygous clones had in fact undergone an unexpected, long-range editing event on one allele (Fig. S10A,B). This was apparent from the lack of amplification of both alleles, as determined by the presence of only one variant type at a highly polymorphic site in the human genome, whereas the remaining WT and heterozygous clones confirmed that this variant was heterozygous in the parental cells (Fig. S10A-C). The extent of the damage was only determined in one clone, #9, in which a 163 bp deletion was detected in the sequence. For the remaining four clones, the damage appeared to completely prevent amplification of the second allele. This highlights the importance of in-depth genotyping following CRISPR-Cas9-mediated mutagenesis, as previously noted (Kosicki et al., 2018; Cullot et al., 2019; Rayner et al., 2019; Przewrocka et al., 2020; Alanis-Lobato et al., 2021). Therefore, we sought to test whether clones #18 and #19, compound mutants with two frame-shifted alleles predicted to introduce premature stop codons in the sequence encoding the third zinc fingers (Fig. S10D-F), were null for *KLF17* expression.

**Fig. 6.**
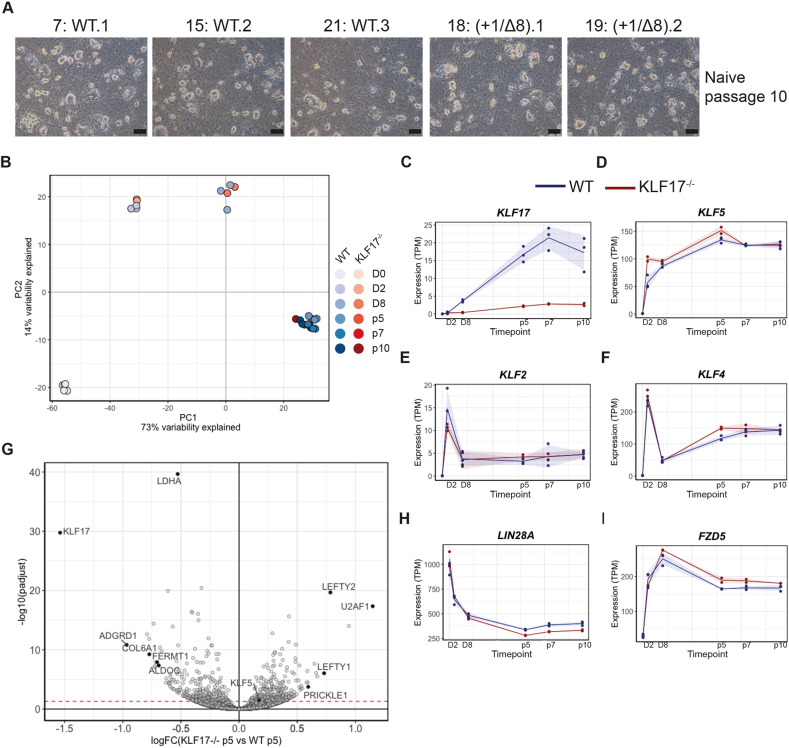
***KLF17*-null hESCs are capable of attaining and maintaining naïve pluripotency.** (A) Representative brightfield images of WT and *KLF17*^−/−^ H9 hESCs following ten passages under naïve culture conditions. (B) Dimensionality reduction by PCA of bulk RNA-seq data collected at various times during the epigenetic resetting (Guo et al., 2017) of WT and *KLF17*^−/−^ H9 hESCs. (C-F) Normalised expression (transcripts per million; TPM) of individual genes of interest across the full resetting time course showing (C) lack of appreciable *KLF17* transcripts and (D) temporally limited upregulation of the paralogue *KLF5* in *KLF17*^−/−^ H9 hESCs and equivalent expression of the paralogues (E) *KLF2* and (F) *KLF4*. Solid lines show the mean value and shading shows the mean±s.d. (G) Volcano plot displaying relative expression of all detected genes in *KLF17*^−/−^ versus WT naïve H9 hESCs following five passages in naïve culture conditions [logFC(*KLF17*^−/−^ p5 versus WT p5)] against the significance of differential expression [-log10(padjust)]. The red-dashed line indicates *P*_adj_=0.05. Individual genes of interest are displayed as black-filled circles and labelled with the gene name. (H,I) Normalised expression (TPM) of individual genes of interest across the full resetting time course showing (H) downregulation of the pluripotency-associated factor *LIN28A* and (I) upregulation of the WNT signalling receptor *FZD5* in *KLF17*^−/−^ H9 hESCs. Solid lines show the mean value and shading shows the mean±s.d. Scale bars: 200 μm in A.

We subjected three WT control clones and clones #18 and #19 to chemical resetting (Guo et al., 2017) for 8 days. In control cells, we observed robust co-expression of KLF17 and OCT4, whereas the compound mutants lacked detectable KLF17 (Fig. 5D). To determine whether *KLF17*^−/−^ hESCs were able to adopt a naïve pluripotent state, we repeated the chemical resetting and found that both the WT controls and *KLF17*^−/−^ hESCs could be propagated in tt2iL+Gö conditions for at least ten passages, maintaining typical naïve morphology (Fig. 6A). To identify molecular differences arising in *KLF17*^−/−^ hESCs, we performed mRNA-seq at various timepoints throughout the chemical resetting process (Fig. S11A). The lack of appreciable *KLF17* RNA expression (TPM <5) in the compound mutant clones (Fig. 6C) suggested that the presence of premature termination codons following the CRISPR-Cas9 target site induced nonsense-mediated decay of the mRNA during translation (Nickless et al., 2017). Therefore, clones #18 and #19 were bona fide *KLF17*-null mutant hESCs. Despite this, PCA analysis of all samples revealed tight clustering of WT and *KLF17*^−/−^ hESCs at all timepoints (Fig. 6B) and with previously published data (Collier et al., 2017) (Fig. S11B). This is consistent with the fact that *KLF17*^−/−^ cells were able to reset and survive long term under naïve culture conditions. This may indicate that KLF17 expression is not required for resetting under the given conditions, or there may be redundancy with other genes that compensate for null mutations in *KLF17*.

Interestingly, DESeq2 analysis identified the *KLF17* paralogue *KLF5* as an early DEG, being significantly upregulated in *KLF17*^−/−^ versus WT naïve hESCs at day 2 of the resetting process (Fig. 6D), a timepoint when *KLF2* and *KLF4* were not differentially expressed (Fig. 6E,F). Furthermore, both *KLF5* and *KLF4* were significantly (*P*_adj_<0.05) upregulated following culture of *KLF17*^−/−^ hESCs in naïve conditions up to p5 (Fig. 6D,F), suggesting possible compensation by one or more KLF paralogues.

Despite this possible redundancy and the clear lack of overt phenotype in *KLF17*^−/−^ naïve hESCs, further analysis revealed an increase in the number of DEGs between WT and mutant hESCs at naïve p5, by which point the naïve pluripotent phenotype is suggested to become more stable (Guo et al., 2017). At p5, 316 genes were significantly upregulated and 311 genes were significantly downregulated (*P*_adj_<0.05) (Fig. 6G; Table S5). Among the genes most significantly downregulated was the gene encoding RNA-binding protein LIN28A (Peng et al., 2011), which was persistently downregulated from p5 onwards (Fig. 6H). LIN28A has been implicated in pluripotency regulation (Heo et al., 2008; Kim et al., 2014; Viswanathan et al., 2008; Yu et al., 2007), although a potential role specifically in naïve hESCs has not been explored. Nevertheless, its significant and maintained downregulation may suggest that its expression depends either directly or indirectly on KLF17 in tt2iL+Gö conditions.

A number of rate-limiting enzymes of glycolysis were also significantly downregulated at p5, including *HK2*, *PFKL*, *ENO1*, *ENO2*, *PGK1* and *PKM* (Fig. S11C-H). Moreover, WNT ligands, receptors and scaffolding proteins were upregulated in the mutant cells at p5 (Fig. 6I; Fig. S11I,J; Table S6). Unlike *LIN28A*, however, expression of these transcripts had recovered at later timepoints, supporting the conclusion that expression of KLF17 is not required for the conversion of primed to naïve hESCs.

## DISCUSSION

In this study, we investigated the human EPI-enriched transcription factor KLF17. By IF analysis of developing human blastocysts, we showed that the protein dynamics of KLF17 were remarkably similar to those of the known pluripotency-associated factor SOX2. Both transcription factors displayed widespread expression in the early blastocyst, with gradual restriction to the pluripotent EPI, marked by NANOG expression. This protein expression dynamic is also shared by the core pluripotency regulator OCT4 (Niakan and Eggan, 2013). Therefore, the expression pattern of KLF17 during preimplantation human development is suggestive of a role in pluripotency regulation.

Indeed, we showed that KLF17 induced the expression of a naïve hESC-like transcriptome in primed hESCs and was sufficient for primed-to-naïve hESC conversion. This implies that KLF17 is a powerful inducer of the human naïve pluripotent state *in vitro*. A previous study highlighted that both KLF17 and KLF4 can regulate gene expression through enhancers located within transposable elements (Pontis et al., 2019), which are often situated near to genes involved in EGA. KLF17-overexpressing hESCs were found to upregulate expression of transposons associated with naïve hESCs, leading to induction of genes involved in mitochondrial function, WNT signalling, cell cycle, adhesion and polarity (Pontis et al., 2019). This is in keeping with our transcriptome analysis, which suggests that KLF17 may be involved in direct regulation of various signalling pathways, primarily PI3K-AKT and WNT, with delayed and indirect regulation of naïve pluripotency-associated markers such as DNMT3L and SUSD2. Given the roles of PI3K-AKT (Wamaitha et al., 2020) and WNT signalling in human pluripotency and differentiation (Singh et al., 2012; Bredenkamp et al., 2019b; Mathieu et al., 2019), and the importance of WNT inhibition for recent methods of naïve pluripotency establishment (Zimmerlin et al., 2016; Guo et al., 2017; Bredenkamp et al., 2019b), we hypothesise that KLF17 acts to dampen WNT signalling endogenously to promote naïve pluripotency and inhibit prodifferentiation cues.

Nonetheless, we found that KLF17-driven resetting was successful only in PXGL medium but not in tt2iL+Gö. This suggests a requirement for exogenous WNT inhibition, implying that the effect of KLF17 expression on WNT signalling activity may not be sufficient alone, or sufficiently rapid, to enable the primed-to-naïve transition. This contrasts with KLF4, which was found to be sufficient for such conversion in t2iLGöY medium (Liu et al., 2017). Differences in the timing and levels of exogenous expression between the Sendai and mRNA methods used for KLF4 (Liu et al., 2017) and the Dox-inducible overexpression system used for KLF17 (this work) might account for this discrepancy. Alternatively, it may suggest that KLF4 is a more-potent inducer of the naïve state of human pluripotency. Specifically, it will be interesting to determine whether KLF4 also acts by dampening the expression of WNT signalling components and, in this way, bypasses a requirement for exogenous WNT inhibition.

Nevertheless, we also found that loss of KLF17 function was not detrimental to hESC resetting. This is surprising, given the rapid upregulation of KLF17 expression that has been reported during chemical resetting (Guo et al., 2017) and raises the possibility of genetic compensation. Indeed, *KLF2*, *KLF4* and *KLF5*, which have all been implicated in pluripotency regulation and to have redundant functions with human *KLF17* in mESCs (Yamane et al., 2018), were also rapidly upregulated during the early stages of resetting in both WT and *KLF17*^−/−^ hESCs. Furthermore, we observed the upregulation of *KLF5* during the early stage of resetting, in which hESCs are undergoing global epigenetic ‘opening’ in response to histone deacetylase inhibition (Guo et al., 2017), and again at p5. This suggests that expression of KLF5 may be sufficient to compensate for a function carried out by KLF17 in the WT state.

In human embryo development, the localisation (Fogarty et al., 2021 preprint) and expression dynamics of *KLF5* and *KLF17* are highly correlated [r=0.76 (Yan et al., 2013)], whereas the correlation coefficient of *KLF4* (r=0.48) is lower and *KLF2* is not expressed in the human pluripotent EPI (Blakeley et al., 2015). Additionally, *in vitro* evidence points to overlapping functions at the molecular level. For instance, overexpressing KLF5 in mESCs increases self-renewal through specific upregulation of the AKT coactivator *Tcl1* (Ema et al., 2008). In the present study, we identified a positive correlation between the expression of exogenous *KLF17* and several PI3K-AKT pathway components and, indeed, the paralogue *TCL1B* was significantly upregulated following 5 days of induction of KLF17 expression (Table S1). Further work could address this question of compensation by performing dual knockout of both *KLF17* and *KLF5* in hESCs and investigating the competency of the cells to undergo chemical resetting.

Alternatively, other KLF paralogues, such as KLF4, may functionally compensate (Yamane et al., 2018) or the combinatorial action of numerous naïve hESC-associated transcription factors with overlapping targets might maintain *KLF17*-null naïve hESCs. This would establish KLF17 as a ‘peripheral’ regulator of human pluripotency and suggest that KLF17, although individually dispensable, is able to reinforce the stability of the pluripotent state mediated by the core factors OCT4 and SOX2 (Nichols and Smith, 2012). For instance, knockdown of *Klf2*, *Klf4* or *Klf5* in naïve mESCs does not appear detrimental (Jiang et al., 2008; Yamane et al., 2018) and *Klf2*- or *Klf4*-null mutant embryos are viable through preimplantation development (Wani et al., 1998; Ehlermann et al., 2003). Despite this, all three factors have validated roles in pluripotency (Parisi et al., 2008; Hall et al., 2009; Jiang et al., 2008). This may suggest that only combinatorial mutation of the KLF factors would be sufficient to induce a detrimental phenotype in naïve hESCs, which will be interesting to explore in the future.

Whereas *KLF17*^−/−^ naïve hESCs did not overtly differ from WT counterparts, we did find interesting trends in differential gene expression at p5 of naïve culture, when WNT inhibition by XAV939 is withdrawn. We observed significant downregulation of metabolism and translation, concomitant with upregulation of protein degradation, in *KLF17*^−/−^ naïve hESCs. This could reflect cellular stress reminiscent of proteasomal inhibition of primed hESCs (Saez et al., 2018), or could result from misexpression of specific genes, such as the RNA-binding protein LIN28A, which has been directly implicated in the growth and survival of hESCs (Peng et al., 2011). Interestingly, LIN28 has been recently identified as a naïve-specific marker in porcine ESCs (Chen et al., 2020) at both the RNA and protein level, suggesting that its downregulation may also be detrimental in naïve hESCs. Thus, it will be interesting to understand whether the transcriptional changes following loss of KLF17 indicate the induction of a cellular stress response or whether there may be further post-transcriptional or translational effects that are not reflected in current transcriptional analyses.

Overall, our overexpression studies showed that KLF17 may typically have a role in regulating naïve pluripotency *in vitro*. Nonetheless, it is clear from our data that KLF17 expression is not necessary for establishing naïve hESCs via chemical resetting (Guo et al., 2017). However, the effect of KLF17 loss in established naïve hESCs or the human preimplantation embryo remains unexplored. Furthermore, a functional requirement for KLF17 expression has been demonstrated under alternative culture conditions for naïve-like hESCs (Bayerl et al., 2021), suggesting that its relative importance may be context specific. Therefore, we theorise that, in a WT situation, KLF17 may act as a peripheral pluripotency factor in human naïve pluripotency, acting alongside a core pluripotency network of OCT4 and SOX2 to maintain robustness of the pluripotent state and prevent premature differentiation.

However, we also note that the lack of KLF17 necessity in naïve hESC establishment does not rule out a more-central role in pluripotency in the human embryo. To date, there have been no systematic comparisons of the outcomes of specific gene modulation in naïve hESCs versus the human pluripotent epiblast, but evidence suggests that they would not necessarily be conserved. For instance, *Nanog*-null naïve mESCs, although prone to differentiation, are still functionally pluripotent, with the capability for chimaera formation (Chambers et al., 2007). In contrast, a *Nanog*-null mouse embryo is unable to form a functional blastocyst or continue development from the peri-implantation stage onward (Mitsui et al., 2003; Chambers et al., 2003; Messerschmidt and Kemler, 2010; Frankenberg et al., 2011). Furthermore, whereas knockdown of *POU5F1* in hESCs causes the expected differentiation phenotype (Wang et al., 2012), even partial loss of OCT4 function in the human embryo has a more-drastic phenotype, with non-cell-autonomous effects across all three lineages at the blastocyst stage (Fogarty et al., 2017). For this reason, future investigation of the function of KLF17 in human *in vivo* pluripotency is an important next step.

## MATERIALS AND METHODS

### Human embryo thaw and culture conditions

Human embryos at various developmental stages that were surplus to family-building desires were donated to the Francis Crick Institute for use in research projects under the UK Human Fertilisation and Embryology Authority Licence number R0162 and Cambridge Central Research Ethics Committee number 16/EE/0067. Informed consent was obtained from all those who donated spare embryos following IVF treatment. Before giving consent, people donating embryos were provided with all the necessary information about the research project and an opportunity to receive counselling.

Slow-frozen blastocysts (day 5 and day 6) were thawed using the BlastThaw kit (Origio; 10542010A) following the manufacturer's instructions. Vitrified blastocysts (day 5 and day 6) were thawed using the Vit Kit-Thaw system (Irvine Scientific; 90137-SO) following the manufacturer's instructions. Human embryos were cultured in pre-equilibrated Global Media (LifeGlobal; LGGG-020) supplemented with 5 mg/ml LifeGlobal HSA (LifeGlobal; LGPS-605), overlaid with mineral oil (Origio; ART-4008-5P) and incubated in an Embryoscope+ time-lapse incubator (Vitrolife).

### Maintenance of standard hESC cultures

hESCs were routinely cultured in mTeSR1 medium (Stem Cell Technologies; 85850) on growth factor-reduced Matrigel-coated dishes (BD Biosciences; 356231) and passaged as clumps at a ∼1:20 ratio using ReLeSR (Stem Cell Technologies; 05872). Cells were maintained in humidified incubators at 37°C, 5% CO_2_. The H9 cell line was obtained under licence and SLA agreement with WiCell. This cell line has been exhaustively tested, including STR profiling, karyotyping, gene expression. The cell lines were subjected to monthly mycoplasma testing in-house and found to be negative.

### Naïve hESC culture

All naïve hESCs were cultured at 5% O_2_ and 5% CO_2_, according to recently published protocols (Guo et al., 2017; Bredenkamp et al., 2019b) on mitotically inactivated DR4 mouse embryonic fibroblasts (MEFs; prepared in-house using a protocol adapted from Nagy, 2003) plated at a density of 1×10^6^ per well of a six-well plate 12-16 h prior to hESC seeding. Naïve hESCs were passaged as single cells by 4 min treatment with Accutase (Thermo Fisher Scientific; A11105-01) at 37°C, at split ratios between 1:3 and 1:6, every 3 to 6 days. For culture in tt2iL+Gö, 10 μM ROCK inhibitor (Tocris Bioscience; Y-27632) was added overnight before and after passaging, to aid survival.

In-house generated, chemically reset, naïve H9 cells were maintained in tt2iL+Gö, with 0.3 μM CHIR99021 (Guo et al., 2017), and XAV supplementation until naïve p5.

### Generation and culture of overexpression hESC lines

Dox-inducible overexpression of HA-tagged KLF17 was achieved using the Lenti-X Tet-On 3G Inducible Expression System (Clontech; 631363) following the manufacturer's protocol, and as outlined previously (Wamaitha et al., 2015). Lentiviral packaging was achieved using 7 μg transgene-containing plasmid and the Lenti-X Packaging Single Shot reagents. Lentiviral supernatant was harvested after 48 h and concentrated by ultracentrifugation. To produce stably transduced cells, hESCs were plated under standard conditions and changed into fresh medium the following morning. Then, 24 h post-plating, 10 μl concentrated virus was added to hESCs for transduction overnight (∼16 h). hESCs were dual selected with 150 μg/ml G418 and 0.5 μg/ml puromycin 48 h post-transduction. For induction of transgene expression, Dox was added to mTeSR1 medium at 1 μg/ml. For the RNA-seq experiments, *KLF17*-inducible hESCs were plated as normal and induction initiated after 24 h by addition of 1 μg/ml Dox to the culture medium (mTeSR1). At ∼30 h, a day 0 (pre-induction) control sample was collected, then both induced (+Dox) and UI samples were collected at 24 h intervals from 48 h (day 1 post-induction) until 144 h (day 5 post-induction). RNA was extracted from the samples and subjected to bulk RNA-seq.

### KLF17-driven resetting of primed to naïve hESCs

H9 KLF17-HA-inducible hESCs were pretreated overnight with 10 μM ROCKi, then harvested from standard culture (mTeSR1 on Matrigel) by 5 min incubation at 37°C with Accutase, resuspended in culture medium supplemented with 10 μM ROCKi and counted. Then, 2×10^5^ hESCs were plated per well of a six-well plate precoated in DR4 MEFs and placed at 5% O_2_ and 5% CO_2_ for ∼24 h. The following day (day 0), medium was changed to PXGL supplemented with 1 μg/ml Dox. From day 2, medium was replenished each day with PXGL freshly supplemented with 1 μg/ml Dox. On day 5, cells were passaged by 4 min incubation in Accutase and plated in PXGL with 10 μM ROCKi at a split ratio between 1:5 and 1:20, dependent upon density. Within 24 h, the cells adopted a domed morphology with highly refractile colony edges. Cells were passaged again on day 7 or 8 and were subsequently maintained in a similar way to chemically reset cells (Guo et al., 2017), with passaging every 3 to 4 days at split ratios of between 1:3 and 1:6.

### Design of gRNAs

gRNAs were designed in a nonbiased manner against the whole cDNA sequence using a standard design tool (Hsu et al., 2013). Two strategies were attempted to achieve functional knockout of KLF17. First, the initiating methionine was targeted to disrupt potentially the entire coding sequence, leading to complete loss of KLF17 expression. Alternatively, the functional domain was targeted to disrupt DNA binding directly or to introduce a premature termination codon, leading to production of a nonfunctional protein. For initial screening, gRNAs were selected on the following criteria: (1) *in silico* score ≥60; (2) identified off-target sites had three or more mismatches; (3) there were no (or very low frequency, ≤0.1%) single nucleotide polymorphisms (SNPs) in the target sequence; and (4) the gRNA target site fell across an annotated DNA-binding domain.

### Transient nucleofection of hESCs

For cell line testing of CRISPR-Cas9 efficiency, gRNAs were individually cloned into pSpCas9(BB)-2A-Puro (PX459) V2.0 (Addgene 62988) (Ran et al., 2013), using the BbsI restriction sites. Nucleofection was carried out on an Amaxa 4D-Nucleofector (Lonza; AAF-1002B, AAF-1002X) with 4 μg plasmid. Then, 24 h prior to nucleofection, H9 hESCs were treated with 10 μM Y-27632 (Tocris Bioscience; 1254). hESCs were harvested as single cells by Accutase treatment (5 min, 37°C) and counted with an automatic cell counter (Nucleocounter NC-200, ChemoMetec). For each gRNA, 2×10^6^ cells were resuspended in 100 μl P3 Primary Cell 4D-Nucleofector X Solution (Lonza; V4XP-3024) and transferred to nucleocuvettes with 4 μg plasmid. Nucleofection was performed with the pre-set H9 hESC program (CB-150). Cells were then resuspended in antibiotic-free mTeSR1 medium supplemented with 10 μM Y-27632 and plated across half of a six-well plate coated with DR4 MEFs to aid attachment and survival. After 24 h, medium was changed to mTeSR1 supplemented with 0.5 μg/ml puromycin for 48 h. Cells were allowed to recover for 8 days prior to harvesting for DNA extraction and assessment of CRISPR-Cas9 editing efficiency by MiSeq analysis. On-target editing was assessed by next-generation sequencing on the MiSeq platform (Illumina), and editing efficiency determined by analysing the FastQ files using both the Cas-Analyzer tool from CRISPR RGEN Tools (Park et al., 2017) and the CrispRVariants package in R (Lindsay et al., 2016).

### Generation of clonal knockout hESCs

H9 hESCs were first nucleofected with 4 μg pSpCas9(BB)-2A-Puro (PX459) V2.0 containing the gRNA KLF17(3_1) as described above. Following 48 h of treatment with 0.5 μg/ml puromycin, cells were allowed to recover on DR4 MEFs for ∼10 days, then manually passaged as single cells following treatment with Accutase (5 min, 37°C) or Accumax (10 min, 37°C; Thermo Fisher Scientific; 00-4666-56) at clonal density into Matrigel-coated 24-well tissue culture plates (Corning; CLS3524). Cells were subcloned once more by manual picking and single-cell dissociation into 12-well plates; 24 clones were then passaged in duplicate and assessed for KLF17 mutation by on-target Sanger sequencing and MiSeq analysis.

### Immunofluorescence analysis

Cultured cells were fixed with 4% paraformaldehyde (PFA) in PBS for 1 h at 4°C, then permeabilised in PBS containing 0.5% Tween-20 [PBS-T (0.5%)] for 20 min at room temperature. Blocking was carried out for 1 h at room temperature in PBS-T (0.1%) with 10% donkey serum. Primary antibodies were diluted in blocking solution as listed in Table S7, and incubated overnight at 4°C. Cells were washed several times in PBS-T (0.1%), then incubated with secondary antibodies (Table S7) in blocking solution for 1 h at room temperature. Following repeated washing, cells were treated with DAPI-Vectashield mounting medium (Vector Labs; H1200) at a 1:30 dilution in PBS-T (0.1%), prior to imaging on an Olympus IX73 microscope.

For human embryos, fixation was performed in 4% PFA in PBS for 1 h at 4°C; the embryos were then permeabilised in PBS containing 0.5% Triton-X100 [PBS-Tx (0.5%)] for 20 min at room temperature. Blocking was performed for 1 h at room temperature in PBS-Tx (0.2%) containing 10% donkey serum and 3% bovine serum albumin (BSA). Primary antibodies were diluted in blocking solution as listed in Table S7, and incubated overnight at 4°C. Embryos were washed several times, then incubated with secondary antibodies in blocking solution for 1 h at room temperature. Following repeated washing, embryos were transferred into DAPI-Vectashield mounting medium at a 1:30 dilution in PBS-T (0.1%) on coverslip dishes (MatTek), and imaged on a Leica SP8 inverted confocal microscope.

### RNA isolation from hESCs and qRT-PCR

RNA was isolated using TRI reagent (Sigma Aldrich; 93289) and DNase I-treated (Ambion; AM2222). cDNA was synthesised using a Maxima first strand cDNA synthesis kit (Thermo Fisher Scientific; R1362). qRT-PCR was performed using a SensiMix SYBR low-ROX kit (Bioline; QT625-05) on a QuantStudio5 machine (Thermo Fisher Scientific). Primer pairs used are listed in Table S8. Each sample was run in triplicate. Gene expression was normalised using *GAPDH* as the housekeeping gene, and the results analysed using the ΔΔCt method.

### RNA-sequencing

For RNA-seq, RNA was isolated and DNase treated as above, and libraries were prepared using the polyA KAPA mRNA HyperPrep Kit (Roche; KK8581). The quality of submitted RNA samples and the resulting cDNA libraries was determined by ScreenTape Assay on a 4200 TapeStation (Agilent; G2991BA). Prepared libraries were submitted for single-ended 75 bp sequencing on an Illumina HiSeq 4000 System.

### Genomic DNA extraction

Total genomic DNA was extracted from hESCs using the DNeasy Blood and Tissue Kit (Qiagen; 69506) following the manufacturer's instructions. The concentration and purity of extracted DNA was measured using a NanoDrop spectrophotometer (DeNovix). Genomic DNA was used to assess the genotype at the KLF17 on-target locus using PCR with the primer pairs shown in Table S9.

### Protein extraction and quantification

hESCs were harvested for protein extraction by addition of CelLytic M lysis buffer (Merck; C2978), freshly supplemented with protease inhibitors (PIC, cOmplete, EDTA-free protease inhibitor cocktail; Roche; 1187358001) and phosphatase inhibitors (PhIC, phosSTOP phosphatase inhibitor; Roche; 4906845001) directly onto plated cells. Cells were scraped, then incubated in lysis buffer for 15 min at 4°C. The lysate was collected and clarified by centrifugation at 20,000 ***g*** for 15 min at 4°C. Protein concentration in the lysates was determined using the BCA assay. Proteins were then denatured by addition of 4× Laemmli sample buffer (Bio-Rad; 1610747) and heating at 90°C for 5 min.

### Protein detection by western blotting

Denatured proteins were thawed at 65°C for 5 min and vortexed to ensure homogeneity. Then, 20 μg protein per lane was loaded onto 10% Mini-PROTEAN TGX Stain-free protein gels (Bio-Rad; 4568033), alongside 5 μl PageRuler Prestained Protein Ladder (Thermo Fisher Scientific; 26617), and electrophoresed at 100-200 V for 1-2 h in a Mini-PROTEAN Tetra Vertical Electrophoresis Cell (Bio-Rad; 1658004). Proteins were transferred onto PVDF membranes (TransBlot Turbo Mini PVDF Transfer Packs, Bio-Rad; 1704156) using a Trans-Blot Turbo Transfer System (Bio-Rad; 1704150). PVDF membranes were blocked for 1 h in TBS-T (0.1%) containing 5% nonfat milk and incubated with primary antibodies diluted in either 5% milk or 5% BSA in TBST-T (0.1%), as shown in Table S10, overnight at 4°C. Following washes with TBS-T (0.1%), membranes were incubated with secondary antibodies in 5% milk for 1 h at room temperature. Proteins of interest were visualised using the SuperSignal West Dura Extended Duration Substrate or SuperSignal West Femto Maximum Sensitivity Substrate (Thermo Fisher Scientific; 34075; 34094) and imaged on an Amersham Imager 600RGB (GE Healthcare).

### Bulk RNA-sequencing analysis

‘Trim Galore!’ utility version 0.4.2 was used to remove sequencing adaptors and to quality trim individual reads with the q-parameter set to 20 [https://www.bioinformatics.babraham.ac.uk/projects/trim_galore/ (retrieved 3 May 2017)]. Then, sequencing reads were aligned to the human genome and transcriptome (Ensembl GRCh38 release-89) using RSEM version 1.3.0 (Li and Dewey, 2011) in conjunction with STAR aligner version 2.5.2 (Dobin et al., 2013). Sequencing quality of individual samples was assessed using FASTQC version 0.11.5 [https://www.bioinformatics.babraham.ac.uk/projects/fastqc/ (retrieved 3 May 2017)] and RNA-SeQC version 1.1.8 (DeLuca et al., 2012). Differential gene expression was determined using the R-Bioconductor package DESeq2 version 1.24.0 (Love et al., 2014). Within the DESeq2 package, adjusted *P* values for log-fold changes were calculated using the Benjamini–Hochberg method and the betaPrior parameter was set to ‘TRUE’. For *KLF17*^−/−^ hESCs in naïve conditions, each timepoint was normalised individually to account for the significant cell-state changes occurring across the extended time course of the experiment (∼60 days). Enrichment analysis was performed using the online EnrichR tool (https://maayanlab.cloud/Enrichr/) (Chen et al., 2013; Kuleshov et al., 2016). EnrichR was used to identify those genes from Table S2 that fall into the Kyoto Encyclopedia of Genes and Genomes 2021 category ‘PI3K-Akt signaling pathway’ (Fig. 3F). Batch-corrected PCA figures were created using the ComBat-Seq method described by Zhang et al. (2020) using the R-package sva version 3.32.1.

### Quantification of confocal immunofluorescence data

Nuclei were identified using StarDist (Schmidt et al., 2018) and colocalisation with fluorescent signals from KLF17 (488 nm), SOX2 (594 nm) and NANOG (647 nm) was quantified using a custom CellProfiler pipeline. Briefly, multichannel confocal imaging *z* stacks were split into single-channel image slices for preprocessing using Fiji (Schindelin et al., 2012). Nuclei were identified in image slices using the Versatile model in the StarDist 2D plugin in Fiji (Normalized Image; Percentile Low and Percentile High, 3 and 99.2%, respectively; Probability Threshold and Overlap Threshold 0.5 and 0.4, respectively), using the DAPI channel as input. The output of the StarDist plugin, which consisted of images in which each segmented nucleus is assigned a unique integer pixel value in each image slice, was saved as .TIF files. The remaining fluorescent channels (488, 594 and 647 nm) were processed with a two-pixel-radius median filter. The StarDist output image set was imported into CellProfiler v4.1.3 (McQuin et al., 2018) and the nuclei contained therein were converted into CellProfiler ‘Objects’ using the ConvertImageToObjects module, preserving original labels. Nuclei objects were tracked through the *z* stack using the Center of Mass distance-based TrackObjects module, such that an individual nucleus could be tracked through the entire *z* stack and given a unique identifier. Before tracking, nuclei objects were filtered to retain only objects bigger than 750 pixels, to create spacing between nuclei that overlapped on the *z* axis. Fluorescent images were also imported into CellProfiler, background corrected and the fluorescent signal inside each nucleus was measured using the MeasureObjectIntensity module. The identifier provided by tracking the nuclei was used to aggregate the signal across all image slices for each individual nucleus using a custom MATLAB script. The segmentation output was manually checked and corrected as needed, to avoid false positive and false negative errors. All code for this project is available at https://github.com/todd-fallesen/Niakan_Lab_KLF17.

## Supplementary Material

Supplementary information

Reviewer comments

## References

[DEV199378C1] Alanis-Lobato, G., Zohren, J., Mccarthy, A., Fogarty, N. M. E., Kubikova, N., Hardman, E., Greco, M., Wells, D., Turner, J. M. A. and Niakan, K. K. (2021). Frequent loss of heterozygosity in CRISPR-Cas9-edited early human embryos. *Proc. Natl. Acad. Sci. USA* 118, e2004832117. 10.1073/pnas.2004832117 34050011 PMC8179174

[DEV199378C2] Alessi, D. R., Andjelkovic, M., Caudwell, B., Cron, P., Morrice, N., Cohen, P. and Hemmings, B. A. (1996). Mechanism of activation of protein kinase B by insulin and IGF-1. *EMBO J.* 15, 6541-6551. 10.1002/j.1460-2075.1996.tb01045.x 8978681 PMC452479

[DEV199378C3] Ali, A., Bhatti, M. Z., Shah, A. S., Duong, H. Q., Alkreathy, H. M., Mohammad, S. F., Khan, R. A. and Ahmad, A. (2015a). Tumor-suppressive p53 signaling empowers metastatic inhibitor KLF17-dependent transcription to overcome tumorigenesis in non-small cell lung cancer. *J. Biol. Chem.* 290, 21336-21351. 10.1074/jbc.M114.635730 25911104 PMC4571863

[DEV199378C4] Ali, A., Zhang, P., Liangfang, Y., Wenshe, S., Wang, H., Lin, X., Dai, Y., Feng, X. H., Moses, R., Wang, D. et al. (2015b). KLF17 empowers TGF-beta/Smad signaling by targeting Smad3-dependent pathway to suppress tumor growth and metastasis during cancer progression. *Cell Death Dis* 6, e1681. 10.1038/cddis.2015.48 25766320 PMC4385926

[DEV199378C5] Bayerl, J., Ayyash, M., Shani, T., Manor, Y. S., Gafni, O., Massarwa, R., Kalma, Y., Aguilera-Castrejon, A., Zerbib, M., Amir, H. et al. (2021). Principles of signaling pathway modulation for enhancing human naive pluripotency induction. *Cell Stem Cell* 28, 1549-1565.e12. 10.1016/j.stem.2021.04.001 33915080 PMC8423434

[DEV199378C6] Bernardo, A. S., Jouneau, A., Marks, H., Kensche, P., Kobolak, J., Freude, K., Hall, V., Feher, A., Polgar, Z., Sartori, C. et al. (2018). Mammalian embryo comparison identifies novel pluripotency genes associated with the naive or primed state. *Biol Open* 7, bio033282. 10.1242/bio.033282 30026265 PMC6124576

[DEV199378C7] Blakeley, P., Fogarty, N. M., Del Valle, I., Wamaitha, S. E., Hu, T. X., Elder, K., Snell, P., Christie, L., Robson, P. and Niakan, K. K. (2015). Defining the three cell lineages of the human blastocyst by single-cell RNA-seq. *Development* 142, 3613. 10.1242/dev.131235 26487783 PMC4631772

[DEV199378C8] Boroviak, T., Loos, R., Lombard, P., Okahara, J., Behr, R., Sasaki, E., Nichols, J., Smith, A. and Bertone, P. (2015). Lineage-specific profiling delineates the emergence and progression of naive pluripotency in mammalian embryogenesis. *Dev. Cell* 35, 366-382. 10.1016/j.devcel.2015.10.011 26555056 PMC4643313

[DEV199378C9] Bredenkamp, N., Stirparo, G. G., Nichols, J., Smith, A. and Guo, G. (2019a). The cell-surface marker sushi containing domain 2 facilitates establishment of human naive pluripotent stem cells. *Stem Cell Reports* 12, 1212-1222. 31031191 10.1016/j.stemcr.2019.03.014 PMC6565611

[DEV199378C10] Bredenkamp, N., Yang, J., Clarke, J., Stirparo, G. G., Von Meyenn, F., Dietmann, S., Baker, D., Drummond, R., Ren, Y., Li, D. et al. (2019b). Wnt inhibition facilitates RNA-mediated reprogramming of human somatic cells to naive pluripotency. *Stem Cell Reports* 13, 1083-1098. 10.1016/j.stemcr.2019.10.009 31708477 PMC6915845

[DEV199378C11] Carracedo, A. and Pandolfi, P. P. (2008). The PTEN-PI3K pathway: of feedbacks and cross-talks. *Oncogene* 27, 5527-5541. 10.1038/onc.2008.247 18794886

[DEV199378C12] Chambers, I., Colby, D., Robertson, M., Nichols, J., Lee, S., Tweedie, S. and Smith, A. (2003). Functional expression cloning of Nanog, a pluripotency sustaining factor in embryonic stem cells. *Cell* 113, 643-655. 10.1016/S0092-8674(03)00392-1 12787505

[DEV199378C13] Chambers, I., Silva, J., Colby, D., Nichols, J., Nijmeijer, B., Robertson, M., Vrana, J., Jones, K., Grotewold, L. and Smith, A. (2007). Nanog safeguards pluripotency and mediates germline development. *Nature* 450, 1230-1234. 10.1038/nature06403 18097409

[DEV199378C14] Chen, E. Y., Tan, C. M., Kou, Y., Duan, Q., Wang, Z., Meirelles, G. V., Clark, N. R. and Ma'ayan, A. (2013). Enrichr: interactive and collaborative HTML5 gene list enrichment analysis tool. *BMC Bioinformatics* 14, 128. 10.1186/1471-2105-14-128 23586463 PMC3637064

[DEV199378C15] Chen, Q., Zhang, H., Jiang, H., Zhang, M., Wang, J., Zhao, L., Wang, C., Liu, M. and Li, R. (2020). Conversion between porcine naive-like and primed ESCs and specific pluripotency marker identification. *In Vitro Cell. Dev. Biol. Anim.* 56, 412-423. 10.1007/s11626-020-00448-3 32424450

[DEV199378C16] Collier, A. J., Panula, S. P., Schell, J. P., Chovanec, P., Plaza Reyes, A., Petropoulos, S., Corcoran, A. E., Walker, R., Douagi, I., Lanner, F. et al. (2017). Comprehensive cell surface protein profiling identifies specific markers of human naive and primed pluripotent states. *Cell Stem Cell* 20, 874-890.e7. 10.1016/j.stem.2017.02.014 28343983 PMC5459756

[DEV199378C17] Cullot, G., Boutin, J., Toutain, J., Prat, F., Pennamen, P., Rooryck, C., Teichmann, M., Rousseau, E., Lamrissi-Garcia, I., Guyonnet-Duperat, V. et al. (2019). CRISPR-Cas9 genome editing induces megabase-scale chromosomal truncations. *Nat. Commun.* 10, 1136. 10.1038/s41467-019-09006-2 30850590 PMC6408493

[DEV199378C18] Davidson, K. C., Adams, A. M., Goodson, J. M., Mcdonald, C. E., Potter, J. C., Berndt, J. D., Biechele, T. L., Taylor, R. J. and Moon, R. T. (2012). Wnt/beta-catenin signaling promotes differentiation, not self-renewal, of human embryonic stem cells and is repressed by Oct4. *Proc. Natl. Acad. Sci. USA* 109, 4485-4490. 10.1073/pnas.1118777109 22392999 PMC3311359

[DEV199378C19] Deluca, D. S., Levin, J. Z., Sivachenko, A., Fennell, T., Nazaire, M. D., Williams, C., Reich, M., Winckler, W. and Getz, G. (2012). RNA-SeQC: RNA-seq metrics for quality control and process optimization. *Bioinformatics* 28, 1530-1532. 10.1093/bioinformatics/bts196 22539670 PMC3356847

[DEV199378C20] Deng, Q., Ramskold, D., Reinius, B. and Sandberg, R. (2014). Single-cell RNA-seq reveals dynamic, random monoallelic gene expression in mammalian cells. *Science* 343, 193-196. 10.1126/science.1245316 24408435

[DEV199378C21] Dobin, A., Davis, C. A., Schlesinger, F., Drenkow, J., Zaleski, C., Jha, S., Batut, P., Chaisson, M. and Gingeras, T. R. (2013). STAR: ultrafast universal RNA-seq aligner. *Bioinformatics* 29, 15-21. 10.1093/bioinformatics/bts635 23104886 PMC3530905

[DEV199378C22] Duggal, G., Warrier, S., Ghimire, S., Broekaert, D., Van Der Jeught, M., Lierman, S., Deroo, T., Peelman, L., Van Soom, A., Cornelissen, R. et al. (2015). Alternative routes to induce naive pluripotency in human embryonic stem cells. *Stem Cells* 33, 2686-2698. 10.1002/stem.2071 26108678

[DEV199378C23] Ehlermann, J., Pfisterer, P. and Schorle, H. (2003). Dynamic expression of Kruppel-like factor 4 (Klf4), a target of transcription factor AP-2alpha during murine mid-embryogenesis. *Anat. Rec. A Discov. Mol. Cell Evol. Biol.* 273, 677-680. 10.1002/ar.a.10089 12845703

[DEV199378C24] Ema, M., Mori, D., Niwa, H., Hasegawa, Y., Yamanaka, Y., Hitoshi, S., Mimura, J., Kawabe, Y., Hosoya, T., Morita, M. et al. (2008). Kruppel-like factor 5 is essential for blastocyst development and the normal self-renewal of mouse ESCs. *Cell Stem Cell* 3, 555-567. 10.1016/j.stem.2008.09.003 18983969

[DEV199378C25] Fogarty, N. M. E., Mccarthy, A., Snijders, K. E., Powell, B. E., Kubikova, N., Blakeley, P., Lea, R. A., Elder, K., Wamaitha, S. E., Kim, D. et al. (2017). Genome editing reveals a role for OCT4 in human embryogenesis. *Nature* 550, 67-73. 10.1038/nature24033 28953884 PMC5815497

[DEV199378C26] Fogarty, N. M. E., Abdelbaki, A., Mccarthy, A., Devito, L., Chen, A. E., Munusamy, P., Blakeley, P., Elder, K., Snell, P., Christie, L. et al. (2021). Direct reprogramming of human embryonic to trophoblast stem cells. *bioRxiv*, 2021.08.18.456785.

[DEV199378C27] Frankenberg, S., Gerbe, F., Bessonnard, S., Belville, C., Pouchin, P., Bardot, O. and Chazaud, C. (2011). Primitive endoderm differentiates via a three-step mechanism involving Nanog and RTK signaling. *Dev. Cell* 21, 1005-1013. 10.1016/j.devcel.2011.10.019 22172669

[DEV199378C28] Gao, L., Wu, K., Liu, Z., Yao, X., Yuan, S., Tao, W., Yi, L., Yu, G., Hou, Z., Fan, D. et al. (2018). Chromatin accessibility landscape in human early embryos and its association with evolution. *Cell* 173, 248-259.e15. 10.1016/j.cell.2018.02.028 29526463

[DEV199378C29] Gerri, C., Mccarthy, A., Alanis-Lobato, G., Demtschenko, A., Bruneau, A., Loubersac, S., Fogarty, N. M. E., Hampshire, D., Elder, K., Snell, P. et al. (2020). Initiation of a conserved trophectoderm program in human, cow and mouse embryos. *Nature* 587, 443-447. 10.1038/s41586-020-2759-x 32968278 PMC7116563

[DEV199378C30] Gumireddy, K., Li, A., Gimotty, P. A., Klein-Szanto, A. J., Showe, L. C., Katsaros, D., Coukos, G., Zhang, L. and Huang, Q. (2009). KLF17 is a negative regulator of epithelial-mesenchymal transition and metastasis in breast cancer. *Nat. Cell Biol.* 11, 1297-1304. 10.1038/ncb1974 19801974 PMC2784164

[DEV199378C31] Guo, G., Von Meyenn, F., Santos, F., Chen, Y., Reik, W., Bertone, P., Smith, A. and Nichols, J. (2016). Naive pluripotent stem cells derived directly from isolated cells of the human inner cell mass. *Stem Cell Reports* 6, 437-446. 10.1016/j.stemcr.2016.02.005 26947977 PMC4834040

[DEV199378C32] Guo, G., Von Meyenn, F., Rostovskaya, M., Clarke, J., Dietmann, S., Baker, D., Sahakyan, A., Myers, S., Bertone, P., Reik, W. et al. (2017). Epigenetic resetting of human pluripotency. *Development* 144, 2748-2763. 10.1242/dev.146811 28765214 PMC5560041

[DEV199378C33] Hall, J., Guo, G., Wray, J., Eyres, I., Nichols, J., Grotewold, L., Morfopoulou, S., Humphreys, P., Mansfield, W., Walker, R. et al. (2009). Oct4 and LIF/Stat3 Additively induce krüppel factors to sustain embryonic stem cell self-renewal. *Cell Stem Cell* 5, 597-609. 10.1016/j.stem.2009.11.003 19951688

[DEV199378C34] Hanna, J., Cheng, A. W., Saha, K., Kim, J., Lengner, C. J., Soldner, F., Cassady, J. P., Muffat, J., Carey, B. W. and Jaenisch, R. (2010). Human embryonic stem cells with biological and epigenetic characteristics similar to those of mouse ESCs. *Proc. Natl. Acad. Sci. USA* 107, 9222-9227. 10.1073/pnas.1004584107 20442331 PMC2889088

[DEV199378C35] Harrington, L. S., Findlay, G. M., Gray, A., Tolkacheva, T., Wigfield, S., Rebholz, H., Barnett, J., Leslie, N. R., Cheng, S., Shepherd, P. R. et al. (2004). The TSC1-2 tumor suppressor controls insulin-PI3K signaling via regulation of IRS proteins. *J. Cell Biol.* 166, 213-223. 10.1083/jcb.200403069 15249583 PMC2172316

[DEV199378C36] Heo, I., Joo, C., Cho, J., Ha, M., Han, J. and Kim, V. N. (2008). Lin28 mediates the terminal uridylation of let-7 precursor MicroRNA. *Mol. Cell* 32, 276-284. 10.1016/j.molcel.2008.09.014 18951094

[DEV199378C37] Hsu, P. D., Scott, D. A., Weinstein, J. A., Ran, F. A., Konermann, S., Agarwala, V., Li, Y., Fine, E. J., Wu, X., Shalem, O. et al. (2013). DNA targeting specificity of RNA-guided Cas9 nucleases. *Nat. Biotechnol.* 31, 827-832. 10.1038/nbt.2647 23873081 PMC3969858

[DEV199378C38] Jiang, J., Chan, Y. S., Loh, Y. H., Cai, J., Tong, G. Q., Lim, C. A., Robson, P., Zhong, S. and Ng, H. H. (2008). A core Klf circuitry regulates self-renewal of embryonic stem cells. *Nat. Cell Biol.* 10, 353-360. 10.1038/ncb1698 18264089

[DEV199378C39] Kilens, S., Meistermann, D., Moreno, D., Chariau, C., Gaignerie, A., Reignier, A., Lelievre, Y., Casanova, M., Vallot, C., Nedellec, S. et al. (2018). Parallel derivation of isogenic human primed and naive induced pluripotent stem cells. *Nat. Commun.* 9, 360. 10.1038/s41467-017-02107-w 29367672 PMC5783949

[DEV199378C40] Kim, S. K., Lee, H., Han, K., Kim, S. C., Choi, Y., Park, S. W., Bak, G., Lee, Y., Choi, J. K., Kim, T. K. et al. (2014). SET7/9 methylation of the pluripotency factor LIN28A is a nucleolar localization mechanism that blocks let-7 biogenesis in human ESCs. *Cell Stem Cell* 15, 735-749. 10.1016/j.stem.2014.10.016 25479749 PMC4258232

[DEV199378C41] Kimber, S. J., Sneddon, S. F., Bloor, D. J., El-Bareg, A. M., Hawkhead, J. A., Metcalfe, A. D., Houghton, F. D., Leese, H. J., Rutherford, A., Lieberman, B. A. et al. (2008). Expression of genes involved in early cell fate decisions in human embryos and their regulation by growth factors. *Reproduction* 135, 635-647. 10.1530/REP-07-0359 18411410

[DEV199378C42] Kosicki, M., Tomberg, K. and Bradley, A. (2018). Repair of double-strand breaks induced by CRISPR-Cas9 leads to large deletions and complex rearrangements. *Nat. Biotechnol.* 36, 765-771. 10.1038/nbt.4192 30010673 PMC6390938

[DEV199378C43] Kuleshov, M. V., Jones, M. R., Rouillard, A. D., Fernandez, N. F., Duan, Q., Wang, Z., Koplev, S., Jenkins, S. L., Jagodnik, K. M., Lachmann, A. et al. (2016). Enrichr: a comprehensive gene set enrichment analysis web server 2016 update. *Nucleic Acids Res.* 44, W90-W97. 10.1093/nar/gkw377 27141961 PMC4987924

[DEV199378C44] Lamothe, B., Yamada, M., Schaeper, U., Birchmeier, W., Lax, I. and Schlessinger, J. (2004). The docking protein Gab1 is an essential component of an indirect mechanism for fibroblast growth factor stimulation of the phosphatidylinositol 3-kinase/Akt antiapoptotic pathway. *Mol. Cell. Biol.* 24, 5657-5666. 10.1128/MCB.24.13.5657-5666.2004 15199124 PMC480891

[DEV199378C45] Li, B. and Dewey, C. N. (2011). RSEM: accurate transcript quantification from RNA-Seq data with or without a reference genome. *BMC Bioinformatics* 12, 323. 10.1186/1471-2105-12-323 21816040 PMC3163565

[DEV199378C46] Lindsay, H., Burger, A., Biyong, B., Felker, A., Hess, C., Zaugg, J., Chiavacci, E., Anders, C., Jinek, M., Mosimann, C. et al. (2016). CrispRVariants charts the mutation spectrum of genome engineering experiments. *Nat. Biotechnol.* 34, 701-702. 10.1038/nbt.3628 27404876

[DEV199378C47] Liu, X., Nefzger, C. M., Rossello, F. J., Chen, J., Knaupp, A. S., Firas, J., Ford, E., Pflueger, J., Paynter, J. M., Chy, H. S. et al. (2017). Comprehensive characterization of distinct states of human naive pluripotency generated by reprogramming. *Nat. Methods* 14, 1055-1062. 10.1038/nmeth.4436 28945704

[DEV199378C48] Loewer, S., Cabili, M. N., Guttman, M., Loh, Y. H., Thomas, K., Park, I. H., Garber, M., Curran, M., Onder, T., Agarwal, S. et al. (2010). Large intergenic non-coding RNA-RoR modulates reprogramming of human induced pluripotent stem cells. *Nat. Genet.* 42, 1113-1117. 10.1038/ng.710 21057500 PMC3040650

[DEV199378C49] Love, M. I., Huber, W. and Anders, S. (2014). Moderated estimation of fold change and dispersion for RNA-seq data with DESeq2. *Genome Biol.* 15, 550. 10.1186/s13059-014-0550-8 25516281 PMC4302049

[DEV199378C50] Mathieu, J., Detraux, D., Kuppers, D., Wang, Y., Cavanaugh, C., Sidhu, S., Levy, S., Robitaille, A. M., Ferreccio, A., Bottorff, T. et al. (2019). Folliculin regulates mTORC1/2 and WNT pathways in early human pluripotency. *Nat. Commun.* 10, 632. 10.1038/s41467-018-08020-0 30733432 PMC6367455

[DEV199378C51] Mcquin, C., Goodman, A., Chernyshev, V., Kamentsky, L., Cimini, B. A., Karhohs, K. W., Doan, M., Ding, L., Rafelski, S. M., Thirstrup, D. et al. (2018). CellProfiler 3.0: Next-generation image processing for biology. *PLoS Biol.* 16, e2005970. 10.1371/journal.pbio.2005970 29969450 PMC6029841

[DEV199378C52] Messerschmidt, D. M. and Kemler, R. (2010). Nanog is required for primitive endoderm formation through a non-cell autonomous mechanism. *Dev. Biol.* 344, 129-137. 10.1016/j.ydbio.2010.04.020 20435031

[DEV199378C53] Messmer, T., Von Meyenn, F., Savino, A., Santos, F., Mohammed, H., Lun, A. T. L., Marioni, J. C. and Reik, W. (2019). Transcriptional heterogeneity in naive and primed human pluripotent stem cells at single-cell resolution. *Cell Rep* 26, 815-824.e4. 10.1016/j.celrep.2018.12.099 30673604 PMC6344340

[DEV199378C54] Mitsui, K., Tokuzawa, Y., Itoh, H., Segawa, K., Murakami, M., Takahashi, K., Maruyama, M., Maeda, M. and Yamanaka, S. (2003). The homeoprotein Nanog is required for maintenance of pluripotency in mouse epiblast and ES cells. *Cell* 113, 631-642. 10.1016/S0092-8674(03)00393-3 12787504

[DEV199378C97] Nagy, A. (2003). *Manipulating the Mouse Embryo: a Laboratory Manual*. Cold Spring Harbor, NY: Cold Spring Harbor Laboratory Press.

[DEV199378C55] Nakamura, T., Okamoto, I., Sasaki, K., Yabuta, Y., Iwatani, C., Tsuchiya, H., Seita, Y., Nakamura, S., Yamamoto, T. and Saitou, M. (2016). A developmental coordinate of pluripotency among mice, monkeys and humans. *Nature* 537, 57-62. 10.1038/nature19096 27556940

[DEV199378C56] Niakan, K. K. and Eggan, K. (2013). Analysis of human embryos from zygote to blastocyst reveals distinct gene expression patterns relative to the mouse. *Dev. Biol.* 375, 54-64. 10.1016/j.ydbio.2012.12.008 23261930

[DEV199378C57] Nichols, J. and Smith, A. (2012). Pluripotency in the embryo and in culture. *Cold Spring Harb Perspect Biol* 4, a008128. 10.1101/cshperspect.a008128 22855723 PMC3405859

[DEV199378C58] Nickless, A., Bailis, J. M. and You, Z. (2017). Control of gene expression through the nonsense-mediated RNA decay pathway. *Cell Biosci* 7, 26. 10.1186/s13578-017-0153-7 28533900 PMC5437625

[DEV199378C59] Ornitz, D. M. and Itoh, N. (2015). The fibroblast growth factor signaling pathway. *Wiley Interdiscip Rev Dev Biol* 4, 215-266. 10.1002/wdev.176 25772309 PMC4393358

[DEV199378C60] Parisi, S., Passaro, F., Aloia, L., Manabe, I., Nagai, R., Pastore, L. and Russo, T. (2008). Klf5 is involved in self-renewal of mouse embryonic stem cells. *J. Cell Sci.* 121, 2629-2634. 10.1242/jcs.027599 18653541

[DEV199378C61] Park, J., Lim, K., Kim, J. S. and Bae, S. (2017). Cas-analyzer: an online tool for assessing genome editing results using NGS data. *Bioinformatics* 33, 286-288. 10.1093/bioinformatics/btw561 27559154 PMC5254075

[DEV199378C62] Pastor, W. A., Liu, W., Chen, D., Ho, J., Kim, R., Hunt, T. J., Lukianchikov, A., Liu, X., Polo, J. M., Jacobsen, S. E. et al. (2018). TFAP2C regulates transcription in human naive pluripotency by opening enhancers. *Nat. Cell Biol.* 20, 553-564. 10.1038/s41556-018-0089-0 29695788 PMC5926822

[DEV199378C63] Peng, S., Chen, L. L., Lei, X. X., Yang, L., Lin, H., Carmichael, G. G. and Huang, Y. (2011). Genome-wide studies reveal that Lin28 enhances the translation of genes important for growth and survival of human embryonic stem cells. *Stem Cells* 29, 496-504. 10.1002/stem.591 21425412

[DEV199378C64] Petropoulos, S., Edsgard, D., Reinius, B., Deng, Q., Panula, S. P., Codeluppi, S., Plaza Reyes, A., Linnarsson, S., Sandberg, R. and Lanner, F. (2016). Single-cell RNA-Seq reveals lineage and X Chromosome dynamics in human preimplantation embryos. *Cell* 165, 1012-1026. 10.1016/j.cell.2016.03.023 27062923 PMC4868821

[DEV199378C65] Pontis, J., Planet, E., Offner, S., Turelli, P., Duc, J., Coudray, A., Theunissen, T. W., Jaenisch, R. and Trono, D. (2019). Hominoid-specific transposable elements and KZFPs facilitate human embryonic genome activation and control transcription in naive human ESCs. *Cell Stem Cell* 24, 724-735.e5. 10.1016/j.stem.2019.03.012 31006620 PMC6509360

[DEV199378C66] Przewrocka, J., Rowan, A., Rosenthal, R., Kanu, N. and Swanton, C. (2020). Unintended on-target chromosomal instability following CRISPR/Cas9 single gene targeting. *Ann. Oncol.* 31, 1270-1273. 10.1016/j.annonc.2020.04.480 32422169 PMC7487774

[DEV199378C67] Qin, H., Hejna, M., Liu, Y., Percharde, M., Wossidlo, M., Blouin, L., Durruthy-Durruthy, J., Wong, P., Qi, Z., Yu, J. et al. (2016). YAP Induces human naive pluripotency. *Cell Rep* 14, 2301-2312. 10.1016/j.celrep.2016.02.036 26947063 PMC4807727

[DEV199378C68] Ramos-Ibeas, P., Sang, F., Zhu, Q., Tang, W. W. C., Withey, S., Klisch, D., Wood, L., Loose, M., Surani, M. A. and Alberio, R. (2019). Pluripotency and X chromosome dynamics revealed in pig pre-gastrulating embryos by single cell analysis. *Nat. Commun.* 10, 500. 10.1038/s41467-019-08387-8 30700715 PMC6353908

[DEV199378C69] Ran, F. A., Hsu, P. D., Wright, J., Agarwala, V., Scott, D. A. and Zhang, F. (2013). Genome engineering using the CRISPR-Cas9 system. *Nat. Protoc.* 8, 2281-2308. 10.1038/nprot.2013.143 24157548 PMC3969860

[DEV199378C70] Rayner, E., Durin, M.-A., Thomas, R., Moralli, D., O'cathail, S. M., Tomlinson, I., Green, C. M. and Lewis, A. (2019). CRISPR-Cas9 causes chromosomal instability and rearrangements in cancer cell lines, detectable by cytogenetic methods. *CRISPR J.* 2, 406-416. 10.1089/crispr.2019.0006 31742432 PMC6919265

[DEV199378C71] Rossant, J. (2016). Making the mouse blastocyst: past, present, and future. *Curr. Top. Dev. Biol.* 117, 275-288. 10.1016/bs.ctdb.2015.11.015 26969983

[DEV199378C72] Saez, I., Koyuncu, S., Gutierrez-Garcia, R., Dieterich, C. and Vilchez, D. (2018). Insights into the ubiquitin-proteasome system of human embryonic stem cells. *Sci. Rep.* 8, 4092. 10.1038/s41598-018-22384-9 29511261 PMC5840266

[DEV199378C73] Sarbassov, D. D., Guertin, D. A., Ali, S. M. and Sabatini, D. M. (2005). Phosphorylation and regulation of Akt/PKB by the rictor-mTOR complex. *Science* 307, 1098-1101. 10.1126/science.1106148 15718470

[DEV199378C74] Schindelin, J., Arganda-Carreras, I., Frise, E., Kaynig, V., Longair, M., Pietzsch, T., Preibisch, S., Rueden, C., Saalfeld, S., Schmid, B. et al. (2012). Fiji: an open-source platform for biological-image analysis. *Nat. Methods* 9, 676-682. 10.1038/nmeth.2019 22743772 PMC3855844

[DEV199378C75] Schmidt, U., Weigert, M., Broaddus, C. and Myers, G. (2018). *Cell Detection with Star-Convex Polygons*, pp. 265-273. Cham. Springer International Publishing.

[DEV199378C76] Shahbazi, M. N., Scialdone, A., Skorupska, N., Weberling, A., Recher, G., Zhu, M., Jedrusik, A., Devito, L. G., Noli, L., Macaulay, I. C. et al. (2017). Pluripotent state transitions coordinate morphogenesis in mouse and human embryos. *Nature* 552, 239-243. 10.1038/nature24675 29186120 PMC5768241

[DEV199378C77] Singh, A. M., Reynolds, D., Cliff, T., Ohtsuka, S., Mattheyses, A. L., Sun, Y., Menendez, L., Kulik, M. and Dalton, S. (2012). Signaling network crosstalk in human pluripotent cells: a Smad2/3-regulated switch that controls the balance between self-renewal and differentiation. *Cell Stem Cell* 10, 312-326. 10.1016/j.stem.2012.01.014 22385658 PMC3294294

[DEV199378C78] Stirparo, G. G., Boroviak, T., Guo, G., Nichols, J., Smith, A. and Bertone, P. (2018). Integrated analysis of single-cell embryo data yields a unified transcriptome signature for the human preimplantation epiblast. *Development* 145, dev158501. 10.1242/dev.158501 29361568 PMC5818005

[DEV199378C79] Takahashi, K. and Yamanaka, S. (2006). Induction of pluripotent stem cells from mouse embryonic and adult fibroblast cultures by defined factors. *Cell* 126, 663-676. 10.1016/j.cell.2006.07.024 16904174

[DEV199378C80] Takashima, Y., Guo, G., Loos, R., Nichols, J., Ficz, G., Krueger, F., Oxley, D., Santos, F., Clarke, J., Mansfield, W. et al. (2014). Resetting transcription factor control circuitry toward ground-state pluripotency in human. *Cell* 158, 1254-1269. 10.1016/j.cell.2014.08.029 25215486 PMC4162745

[DEV199378C81] Theunissen, T. W., Powell, B. E., Wang, H., Mitalipova, M., Faddah, D. A., Reddy, J., Fan, Z. P., Maetzel, D., Ganz, K., Shi, L. et al. (2014). Systematic identification of culture conditions for induction and maintenance of naive human pluripotency. *Cell Stem Cell* 15, 471-487. 10.1016/j.stem.2014.07.002 25090446 PMC4184977

[DEV199378C82] Tremblay, F., Brule, S., Hee Um, S., Li, Y., Masuda, K., Roden, M., Sun, X. J., Krebs, M., Polakiewicz, R. D., Thomas, G. et al. (2007). Identification of IRS-1 Ser-1101 as a target of S6K1 in nutrient- and obesity-induced insulin resistance. *Proc. Natl. Acad. Sci. USA* 104, 14056-14061. 10.1073/pnas.0706517104 17709744 PMC1950339

[DEV199378C83] Van Vliet, J., Crofts, L. A., Quinlan, K. G., Czolij, R., Perkins, A. C. and Crossley, M. (2006). Human KLF17 is a new member of the Sp/KLF family of transcription factors. *Genomics* 87, 474-482. 10.1016/j.ygeno.2005.12.011 16460907

[DEV199378C84] Viswanathan, S. R., Daley, G. Q. and Gregory, R. I. (2008). Selective blockade of microRNA processing by Lin28. *Science* 320, 97-100. 10.1126/science.1154040 18292307 PMC3368499

[DEV199378C85] Wamaitha, S. E., Del Valle, I., Cho, L. T., Wei, Y., Fogarty, N. M., Blakeley, P., Sherwood, R. I., Ji, H. and Niakan, K. K. (2015). Gata6 potently initiates reprograming of pluripotent and differentiated cells to extraembryonic endoderm stem cells. *Genes Dev.* 29, 1239-1255. 10.1101/gad.257071.114 26109048 PMC4495396

[DEV199378C86] Wamaitha, S. E., Grybel, K. J., Alanis-Lobato, G., Gerri, C., Ogushi, S., Mccarthy, A., Mahadevaiah, S. K., Healy, L., Lea, R. A., Molina-Arcas, M. et al. (2020). IGF1-mediated human embryonic stem cell self-renewal recapitulates the embryonic niche. *Nat. Commun.* 11, 764. 10.1038/s41467-020-14629-x 32034154 PMC7005693

[DEV199378C87] Wang, Z., Oron, E., Nelson, B., Razis, S. and Ivanova, N. (2012). Distinct lineage specification roles for NANOG, OCT4, and SOX2 in human embryonic stem cells. *Cell Stem Cell* 10, 440-454. 10.1016/j.stem.2012.02.016 22482508

[DEV199378C88] Wang, Y., Xu, Z., Jiang, J., Xu, C., Kang, J., Xiao, L., Wu, M., Xiong, J., Guo, X. and Liu, H. (2013). Endogenous miRNA sponge lincRNA-RoR regulates Oct4, Nanog, and Sox2 in human embryonic stem cell self-renewal. *Dev. Cell* 25, 69-80. 10.1016/j.devcel.2013.03.002 23541921

[DEV199378C89] Wang, X., Liu, D., He, D., Suo, S., Xia, X., He, X., Han, J. J. and Zheng, P. (2017). Transcriptome analyses of rhesus monkey preimplantation embryos reveal a reduced capacity for DNA double-strand break repair in primate oocytes and early embryos. *Genome Res.* 27, 567-579. 10.1101/gr.198044.115 28223401 PMC5378175

[DEV199378C90] Wani, M. A., Means, R. T., Jr. and Lingrel, J. B. (1998). Loss of LKLF function results in embryonic lethality in mice. *Transgenic Res.* 7, 229-238. 10.1023/A:1008809809843 9859212

[DEV199378C91] Yamane, M., Ohtsuka, S., Matsuura, K., Nakamura, A. and Niwa, H. (2018). Overlapping functions of Kruppel-like factor family members: targeting multiple transcription factors to maintain the naive pluripotency of mouse embryonic stem cells. *Development* 145, dev162404. 10.1242/dev.162404 29739838

[DEV199378C92] Yan, L., Yang, M., Guo, H., Yang, L., Wu, J., Li, R., Liu, P., Lian, Y., Zheng, X., Yan, J. et al. (2013). Single-cell RNA-Seq profiling of human preimplantation embryos and embryonic stem cells. *Nat. Struct. Mol. Biol.* 20, 1131-1139. 10.1038/nsmb.2660 23934149

[DEV199378C93] Yu, J., Vodyanik, M. A., Smuga-Otto, K., Antosiewicz-Bourget, J., Frane, J. L., Tian, S., Nie, J., Jonsdottir, G. A., Ruotti, V., Stewart, R. et al. (2007). Induced pluripotent stem cell lines derived from human somatic cells. *Science* 318, 1917-1920. 10.1126/science.1151526 18029452

[DEV199378C94] Zhang, Y., Parmigiani, G. and Johnson, W. E. (2020). ComBat-seq: batch effect adjustment for RNA-seq count data. *NAR Genom Bioinform* 2, lqaa078. 10.1093/nargab/lqaa078 33015620 PMC7518324

[DEV199378C95] Zhou, S., Tang, X. and Tang, F. (2016). Kruppel-like factor 17, a novel tumor suppressor: its low expression is involved in cancer metastasis. *Tumour Biol.* 37, 1505-1513. 10.1007/s13277-015-4588-3 26662959 PMC4842221

[DEV199378C96] Zimmerlin, L., Park, T. S., Huo, J. S., Verma, K., Pather, S. R., Talbot, C. C., Jr, Agarwal, J., Steppan, D., Zhang, Y. W., Considine, M. et al. (2016). Tankyrase inhibition promotes a stable human naive pluripotent state with improved functionality. *Development* 143, 4368-4380. 10.1242/dev.138982 27660325 PMC5201042

