# Supplementary material for: KLF17 promotes human naïve pluripotency but is not required for its establishment
Source: Development. 2021 Nov 15;148(22):dev199378. doi: 10.1242/dev.199378 (PMC8645209; doi:10.1242/dev.199378)
Supplement: Supplementary information [file develop-148-199378-s1.pdf]

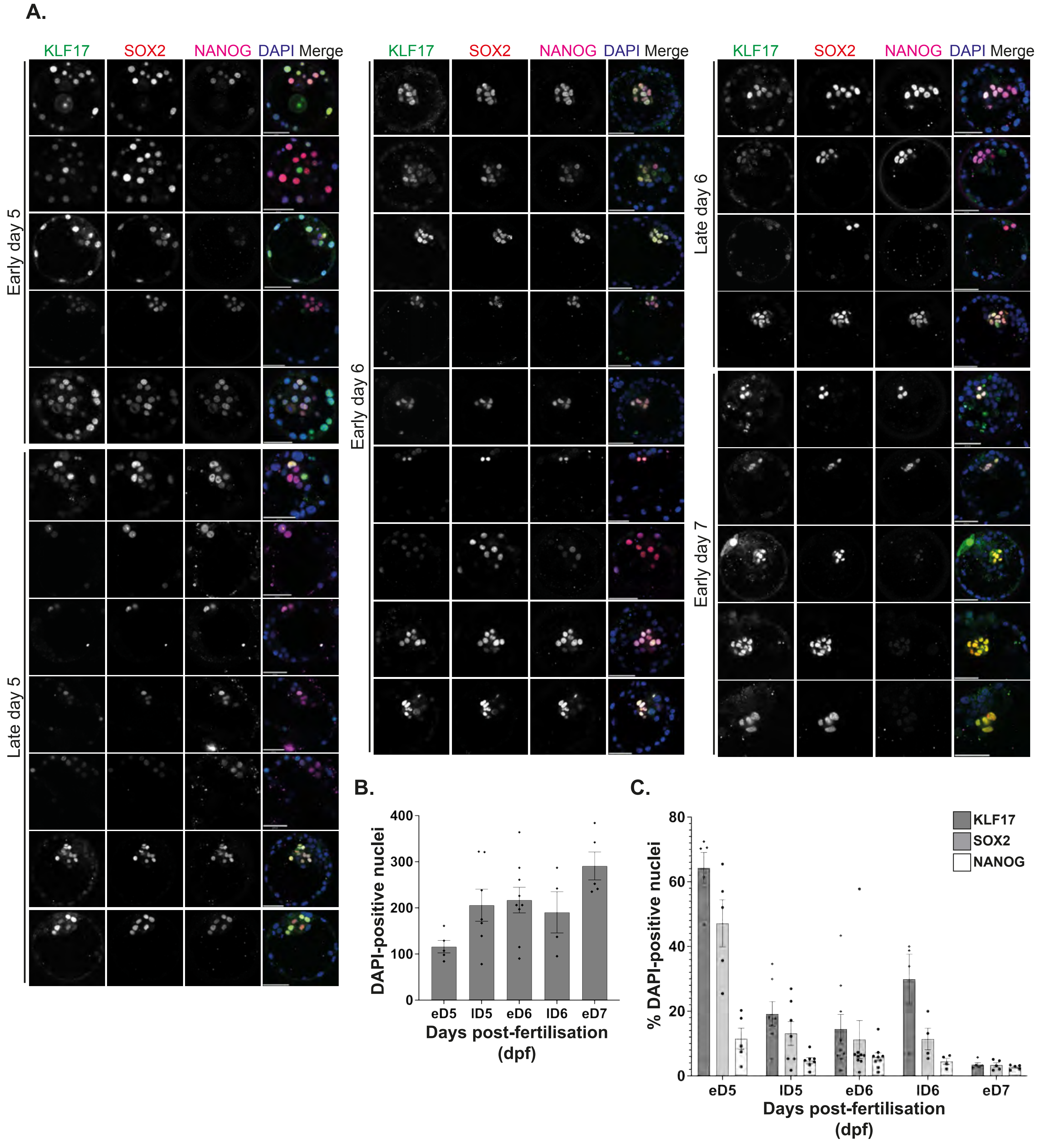

Fig. S1. KLF17 expression in the human embryo is coincident with known pluripotency factors. **(A)** Immunofluorescence analysis of blastocyst-stage human embryos at early day 5 (*n*=5), late day 5 (*n*=7), early day 6 (*n*=9), late day 6 (*n*=4) and early day 7 (*n*=5) post-fertilisation. **(B)** Quantification of the number of segmented DAPI-positive nuclei per embryo across blastocyst development. **(C)** Quantification of the total proportion of DAPI-positive nuclei per embryo that are KLF17-positive, SOX2-positive or NANOG-positive. Bars represent the mean, error bars the s.e.m. and points the percentage in individual embryos. Scale bars: 50 µm in A.

Development • Supplementary information

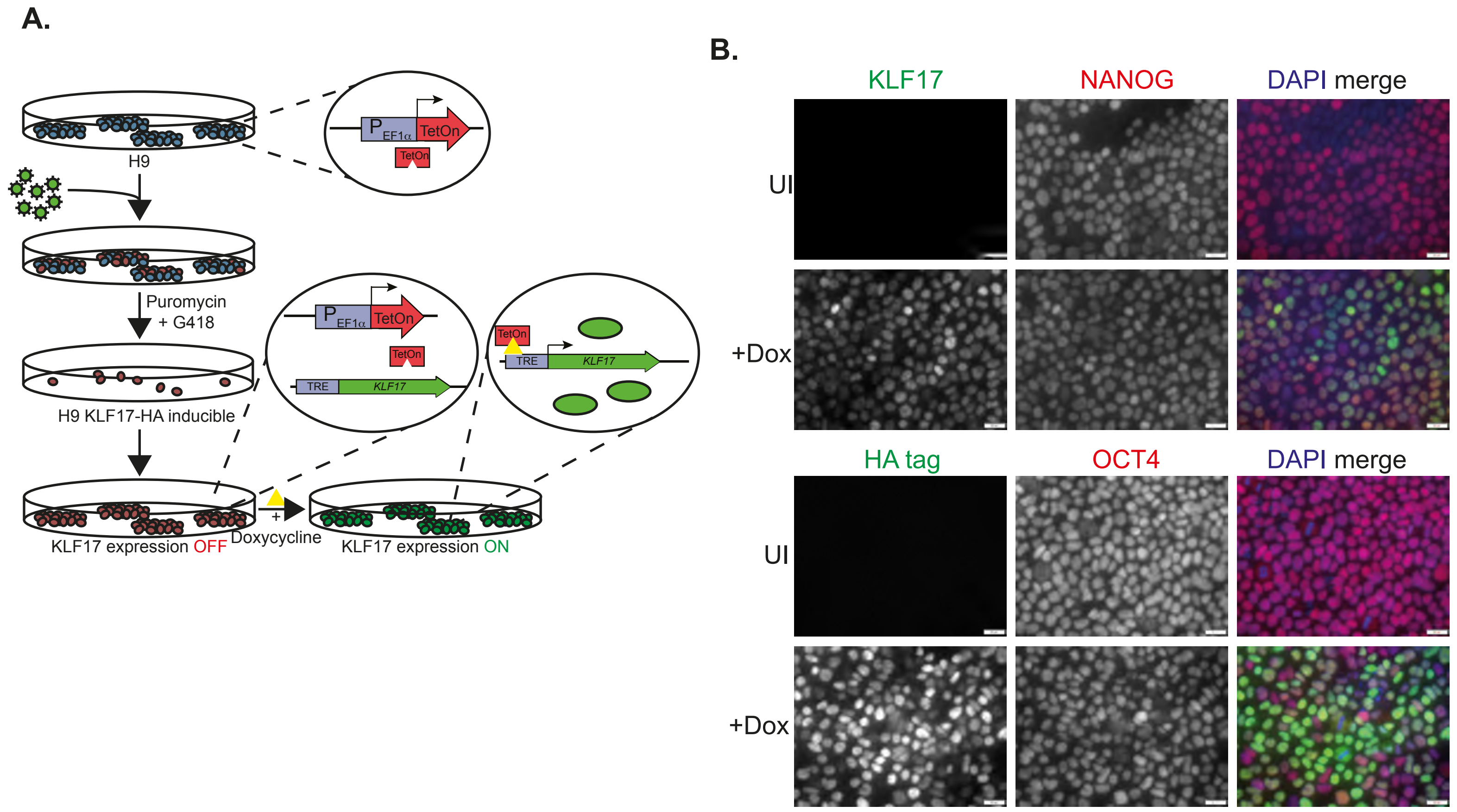

Fig. S2. Generating primed hESCs to inducibly express ectopic HA-tagged KLF17. (A) Schematic diagram showing the generation of H9 KLF17-HA inducible hESCs via lentiviral transduction. **(B)** Immunofluorescence analysis of H9 KLF17-HA inducible hESCs following 5 days uninduced (UI) or 5 days doxycycline induction (+Dox). Scale bars: 20 µm in B. *n* ≥ 3.

Development • Supplementary information

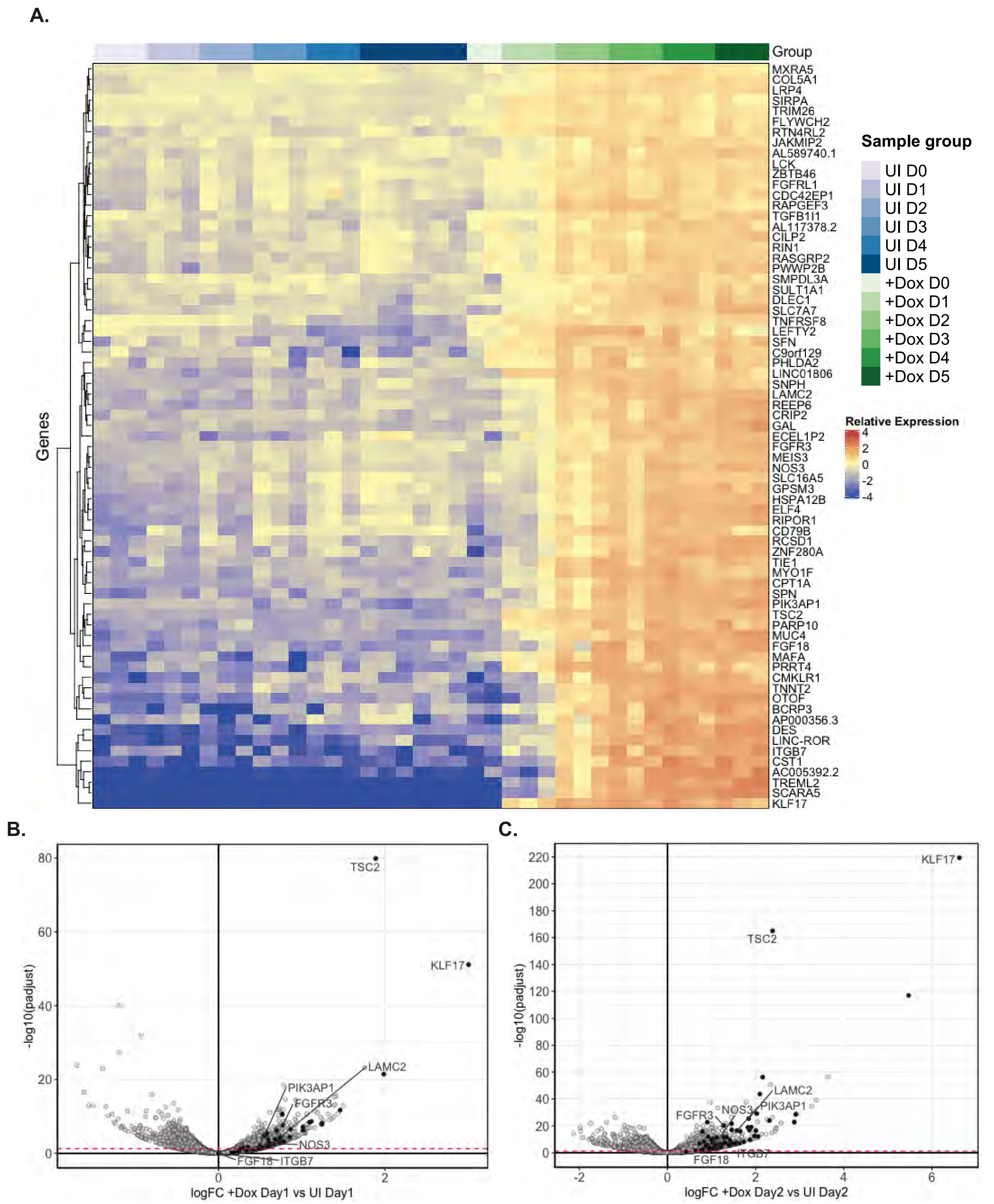

Development • Supplementary information

Fig. S3. 70 genes are strongly correlated with *KLF17* expression over time. (A) Heatmap ordered by sample (UI or +Dox) and time point showing all genes that are highly correlated with *KLF17* across time (Pearson correlation coefficient (r) ≥ 0.85). **(B-C)** Volcano plots displaying relative expression of all detected genes in +Dox versus UI H9 KLF17-HA hESCs at (B) day 1 (logFC(+Dox Day1 vs UI Day1)) and (C) day 2 (logFC(+Dox Day2 vs UI Day2)) against the significance of differential expression (-log10(padjust)). The red-dashed line indicates padj = 0.05. All genes with correlation coefficient to *KLF17* ≥ 0.85 are displayed as filled circles and genes associated with PI3K-AKT signalling (as shown in Fig. 3F) are labelled with the gene name.

Development • Supplementary information

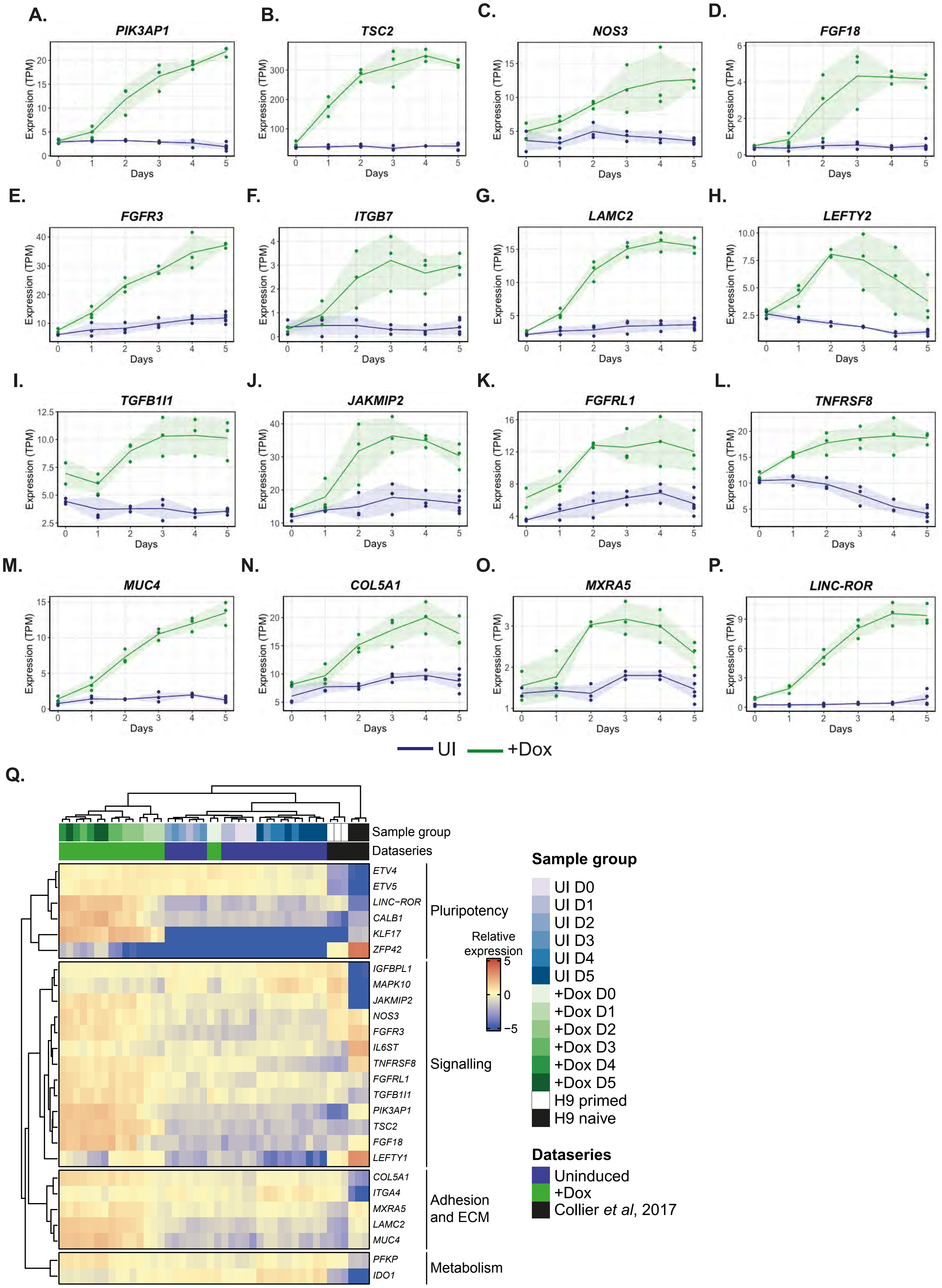

Development • Supplementary information

Fig. S4. Genes highly correlated with *KLF17* expression include numerous signalling components and cytoskeletal/ECM components. (A-P) Normalised expression (TPM) of individual genes of interest across the 5-day time course showing factors involved in (A-G) PI3K-AKT signalling, (H-I) TGFβ signalling, (J-L) other signalling pathways, (F-G,M-O) the cytoskeleton/ICM and (P) the pluripotency-regulating long non-coding RNA *LINC-ROR*. Solid lines show the mean value and shading shows the mean ± s.d. **(Q)** A heatmap depicting the relative expression, based on TPM values, of the genes of interest from A-P and Fig. 3C-D in UI and +Dox H9 KLF17-HA hESCs alongside established primed and naïve hESCs (Collier *et al*., 2017).

Development • Supplementary information

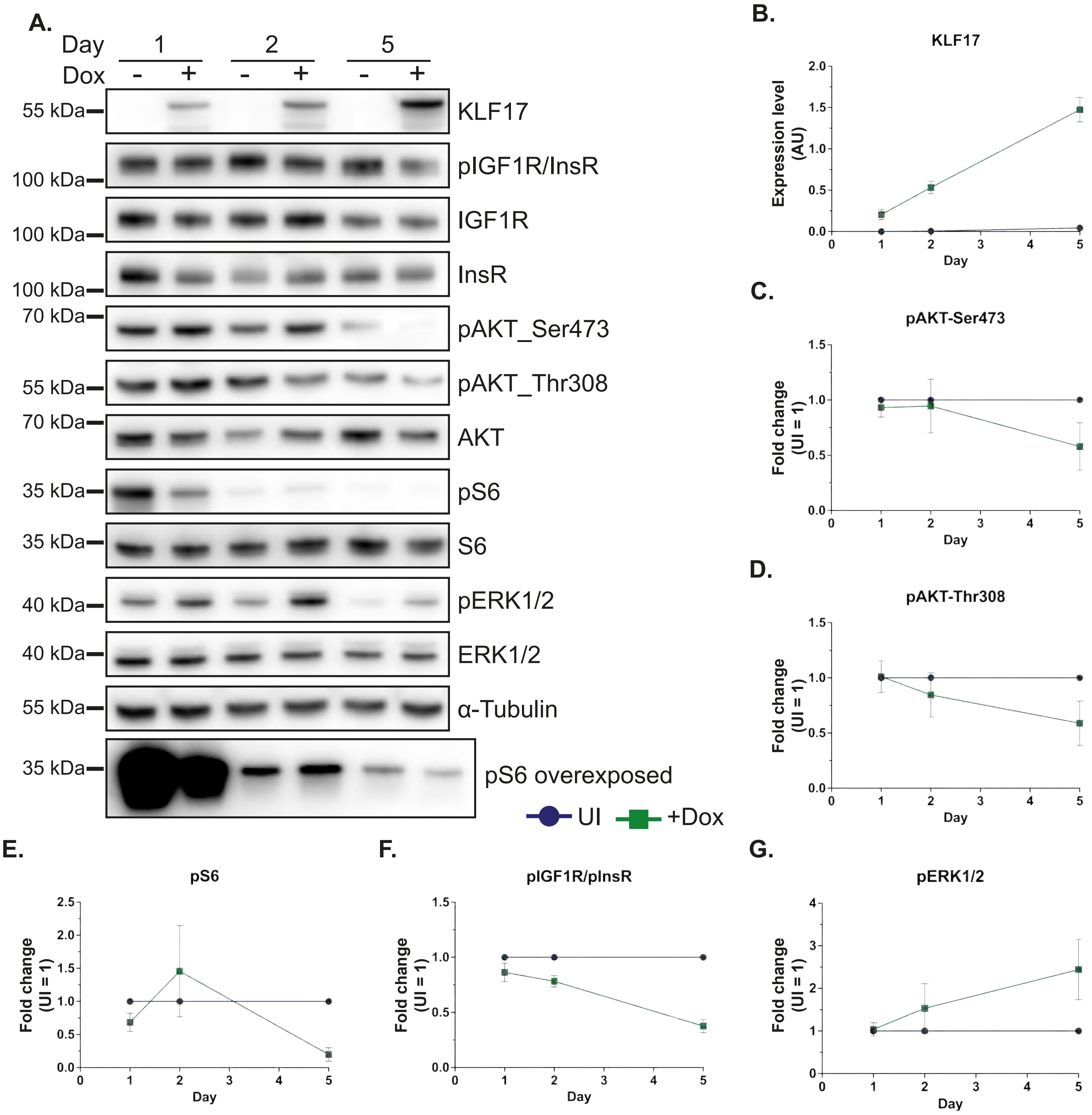

Fig. S5. KLF17 overexpression brings about changes in PI3K-AKT signalling pathway activity. (A) Representative western blot analysis of ectopic KLF17 induction following 1, 2 and 5 days Dox treatment of H9 KLF17-HA and the associated changes in phosphorylation of various components of the PI3K-AKT signalling pathway. **(B-G)** Quantification of the levels of protein detection by western blot showing (B) the steady increase in KLF17 protein levels (arbitrary units, AU) in +Dox versus UI H9 KLF17-HA hESCs and (C-G) the dynamic changes in phosphorylation and activation status of various components of the PI3K-AKT signalling pathway, represented as a fold-change of +Dox versus UI. All intensity values were normalised to the level of the α-Tubulin loading control. Circles and squares represent the mean value and the whiskers the s.e.m. *n*=3.

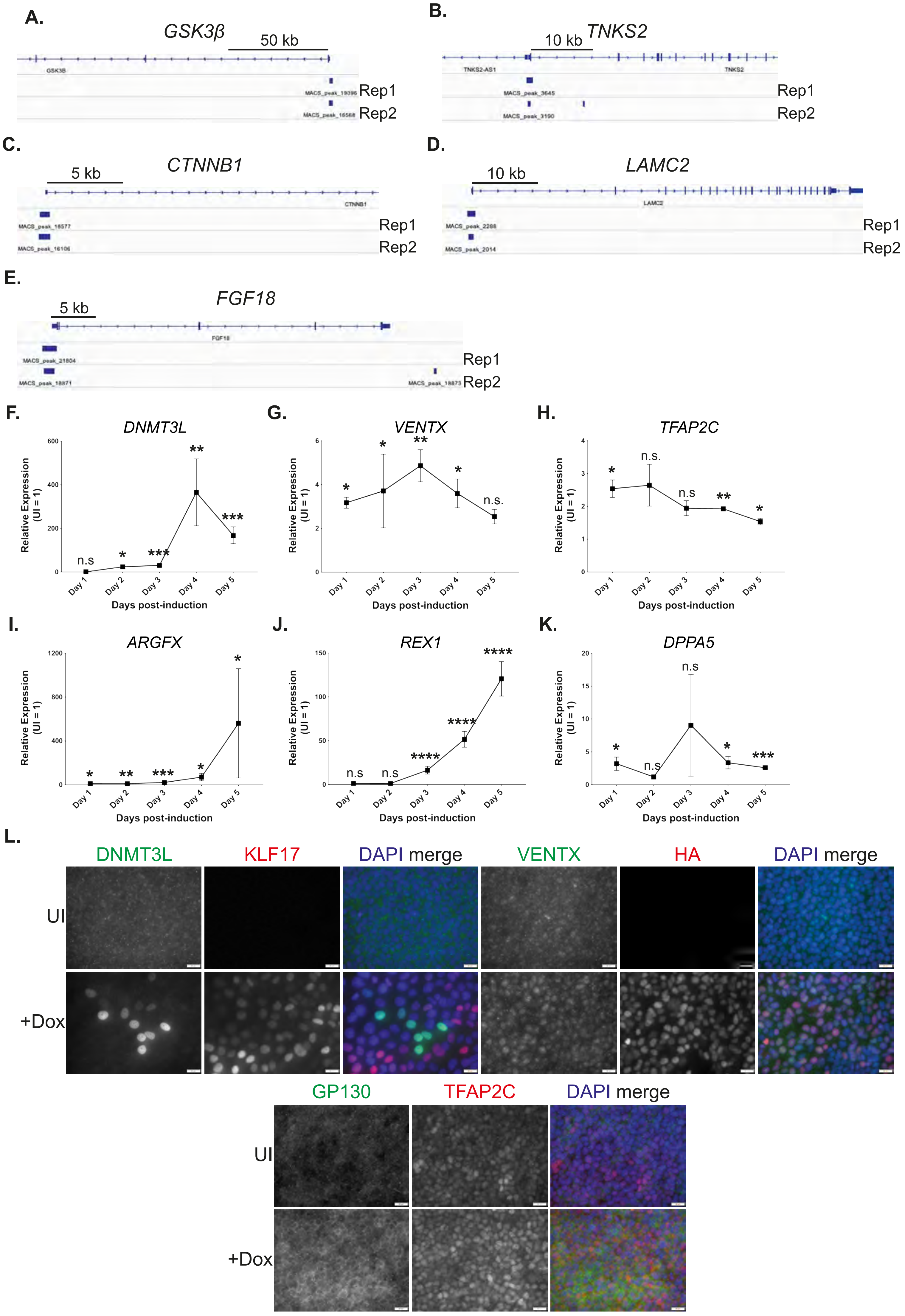

Development • Supplementary information

Fig. S6. Further investigation of putative KLF17 target genes. (A-E) Example tracks from the IGV browser, showing the binding sites of KLF17 near to selected genes of interest, as identified in naïve hESCs cultured under HENSM conditions (Bayerl *et al*., 2021). **(F-K)** qRT-PCR analysis across the 5-day time course of Dox treatment in H9 KLF17-HA hESCs. Relative expression is displayed as fold change versus uninduced cells and normalised to *GAPDH* as a housekeeping gene using the ΔΔCt method. Dots represent the mean and whiskers the s.e.m. Welch's t test; **** $p < 0.001$; *** $p < 0.005$; ** $p < 0.01$; * $p < 0.05$; n.s., not significant; n=3. **(L)** Immunofluorescence analysis of H9 KLF17-HA inducible hESCs following 5 days uninduced (UI) or 5 days doxycycline induction (+Dox). Scale bars: 20 μm in L. $n \geq 3$.

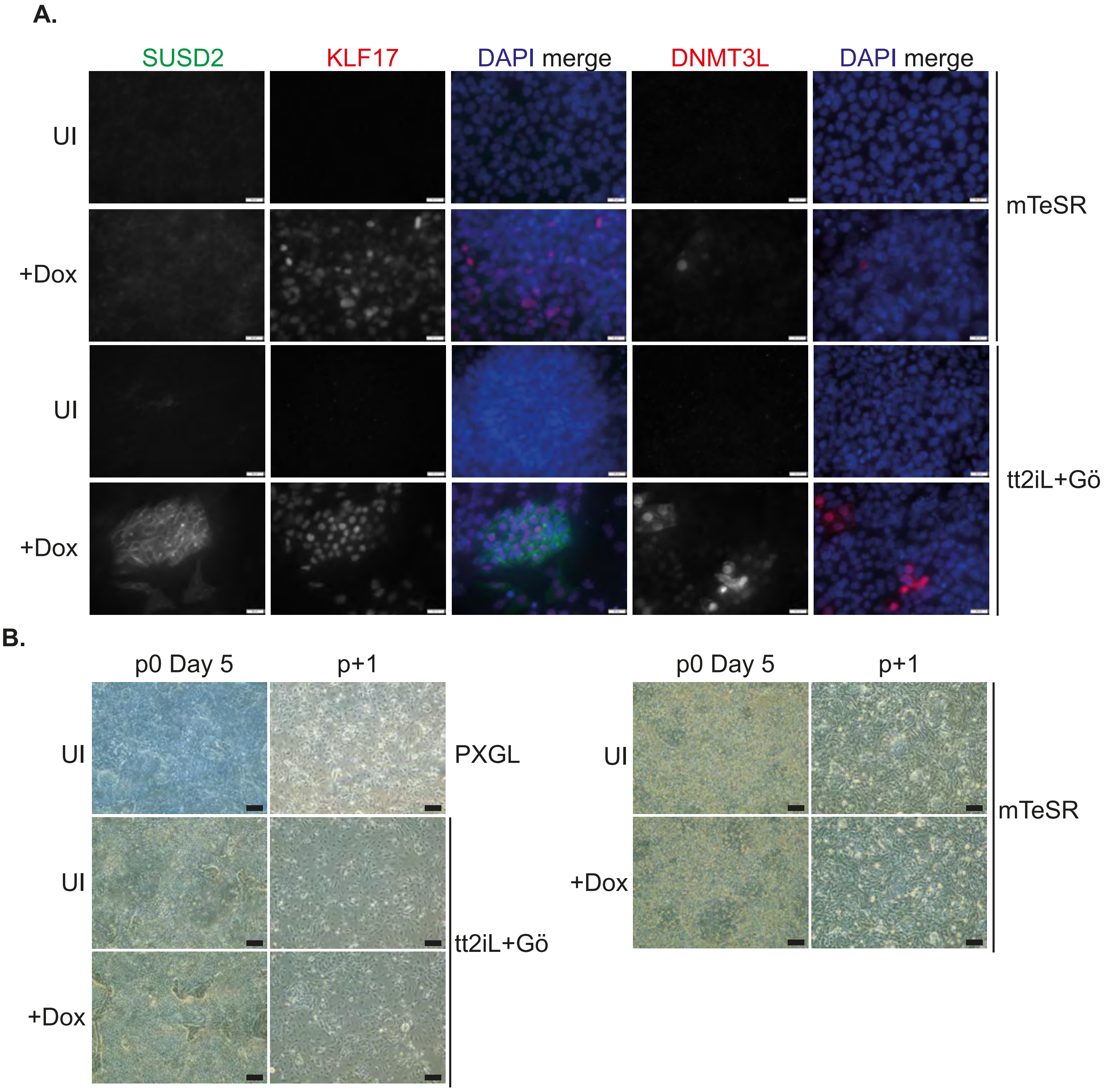

Fig. S7. PXGL is uniquely able to support KLF17-driven naïve resetting of H9 KLF17-HA hESCs **(A)** Immunofluorescence analysis of H9 KLF17-HA inducible hESCs following 5 days uninduced (UI) or 5 days doxycycline induction (+Dox) in the indicated media. Cells were cultured on a mouse embryonic fibroblast (MEF) feeder layer and at 5% O_2. **(B)** Unlike induced cells, UI control H9 KLF17-HA cells were unable to survive in PXGL medium following the first passage. Under mTeSR1 or tt2iL+Gö conditions, neither +Dox nor UI cells transitioned to naïve morphology. Scale bars: 20 μm in A; 200 μm in B. *n* ≥ 3.

Development • Supplementary information

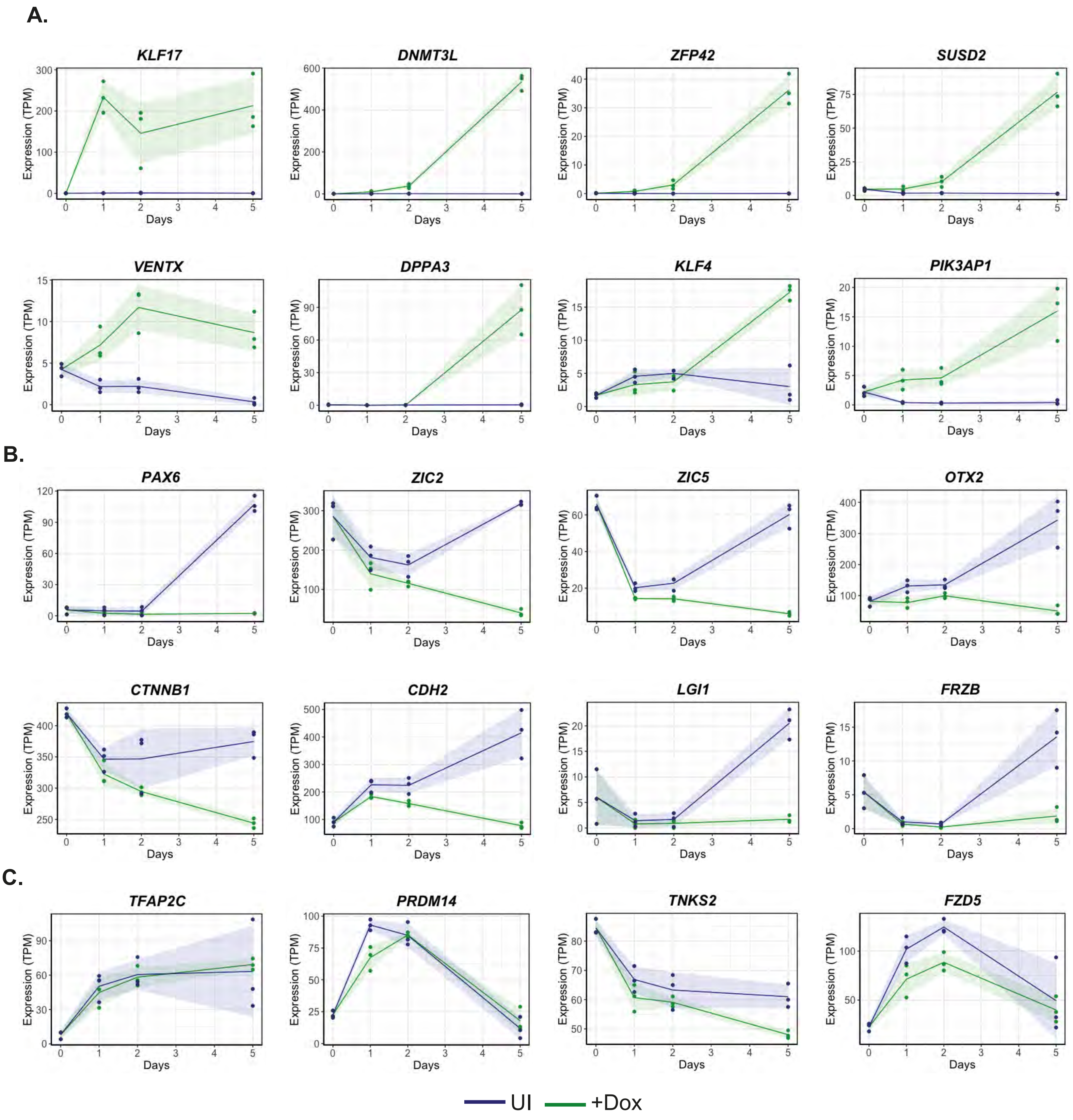

Fig. S8. KLF17 expression in PXGL drives upregulation of naïve-associated genes and downregulation of primed-associated genes. Normalised expression (TPM) of individual genes of interest across the 5-day time course of H9 KLF17-HA in PXGL. **(A)** Examples of naïve hESC-associated genes whose expression is induced only in the presence of ectopic *KLF17* expression. **(B)** Examples of primed hESC-associated genes whose expression is specifically restrained when *KLF17* expression is induced. **(C)** Examples of genes whose expression dynamics are similar irrespective of *KLF17* expression. Solid lines show the mean value and shading shows the mean ± s.d.

A.

gRNA	Direction	Sequence
KLF17(1_1)	Minus	5'-GCCGTACATGAAGACTGGGT-3'
KLF17(1_2)	Minus	5'-GGTCGGCCGTACATGAAGAC-3'
KLF17(3_1)	Plus	5'-TGAGCTTAGACGACATATGC-3'
KLF17(3_2)	Plus	5'-ATGAGCTTAGACGACATATG-3'
KLF17(3_3)	Plus	5'-GAGGCCATATTCTTGCAACT-3'

B.

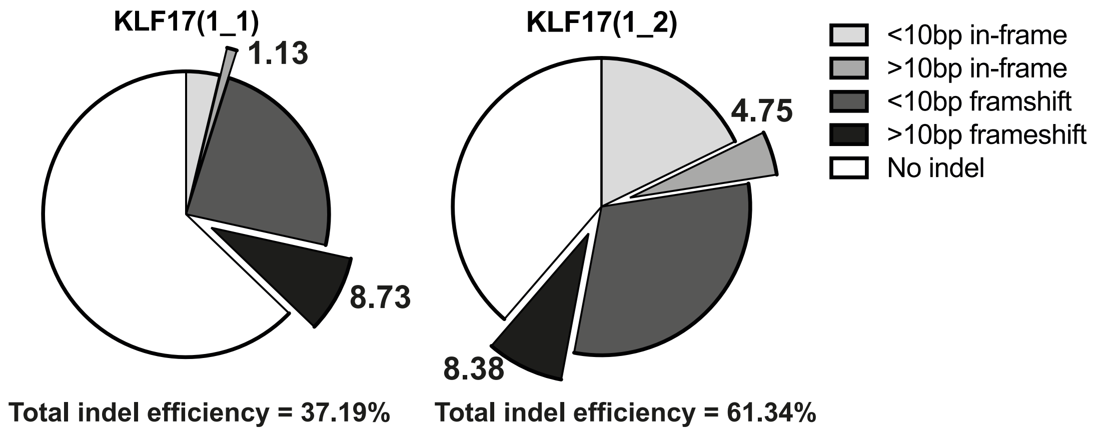

Fig. S9. Generating *KLF17*-null hESCs by CRISPR-Cas9. (A) A table showing the gRNA sequences tested for mutagenic efficiency. **(B)** Pie charts representing the relative proportions of different outcomes of CRISPR-Cas9 editing of H9 hESCs, based on the sequences detected by MiSeq analysis.

Development • Supplementary information

A.

Clone #	1	4	7	8	9	10	11	15	18	19	21
**Short-range genotype**	Δ8	Δ16/Δ21	WT	Δ2	Δ9	Δ8	Δ8	WT	+1/Δ8	+1/Δ8	WT
**Long-range genotype**	Δ8/?	Δ16/Δ21	WT	Δ2/?	Δ9/Δ163	Δ8/?	Δ8/?	WT	+1/Δ8	+1/Δ8	WT

KLF17-null mutants

B.

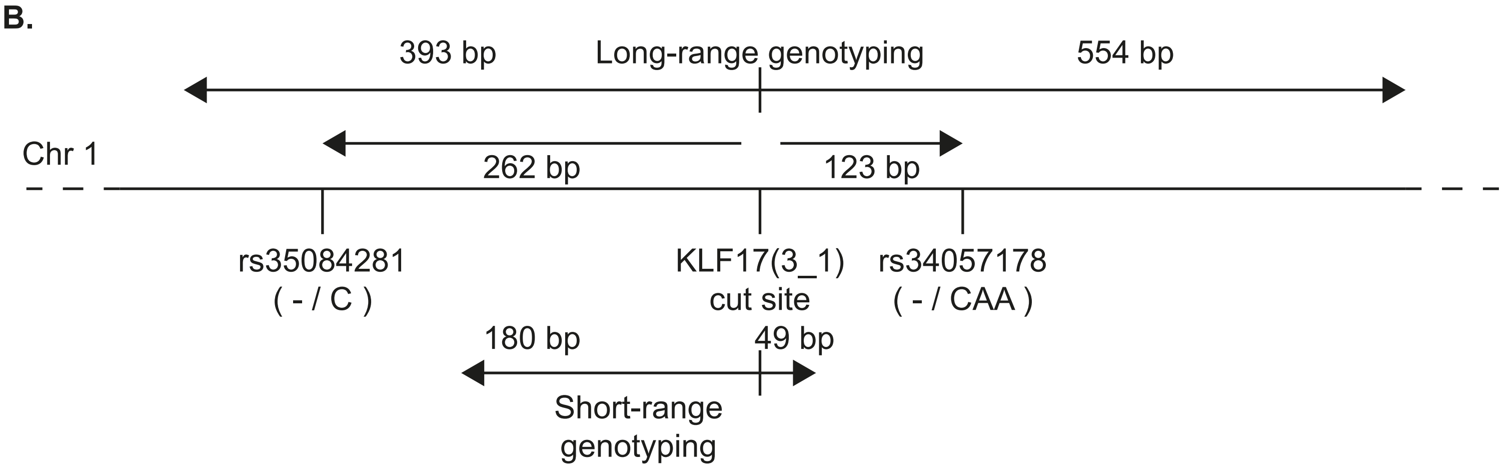

C.

Cell Line	Parental H9	#7	#1	#8	#9	#10	#11
**C / CAA**	53.85%	37.5%	0%	0%	29.17%	0%	0%
**- / -**	46.15%	62.5%	100%	100%	70.83%	100%	100%

D.

HxnH motif — Zinc finger domains

D E L R R H M R V H T R Y R P Y K C D Q C S R
GATGAGCTTAGACGACATATGCGGGTACACACCAGATATCGACCATATAAATGTGATCAGTGCAGCCGA
KLF17(3_1) PAM

Reference target region

E.

WT
D E L R R H M R V H T R Y R P Y K C D Q C S R
GATGAGCTTAGACGACATATGCGGGTACACACCAGATATCGACCATATAAATGTGATCAGTGCAGCCGA

Δ8 bp deletion
GATGAGCTTAGACGACATA--------CACACCAGATATCGACCATATAAATGTGATCAGTGCAGCCGA
D E L R R H T H Q I S T I * M * S V Q P
HxnH motif is disrupted
Premature termination codons - third zinc finger is lost

F.

WT
D E L R R H M R V H T R Y R P Y K C D Q C S R
GATGAGCTTAGACGACATA-TGCGGGTACACACCAGATATCGACCATATAAATGTGATCAGTGCAGCCGA

+1 bp insertion
GATGAGCTTAGACGACATA**A**TGCGGGTACACACCAGATATCGACCATATAAATGTGATCAGTGCAGCCGA
D E L R R H N A G T H Q I S T I * M * S V Q P
HxnH motif is disrupted
Premature termination codons - third zinc finger is lost

Development • Supplementary information

Fig. S10. Genotyping of H9 hESCs following targeting with gRNA KLF17(3_1) and clonal expansion. (A) A table showing the results of genotyping 11 clones generated following CRISPR-Cas9 targeting. Short-range genotype denotes the results of MiSeq of a ~250 bp region surrounding the KLF17(3_1) cut site. Long-range genotype denotes the results of Sanger sequencing of a ~950 bp region surrounding the KLF17(3_1) cut site. The red rectangle highlights the verified KLF17-/- H9 hESCs that were carried forward. **(B)** Schematic of the short- and long-range genotyping approach employed on the 11 clones in (A). **(C)** A table showing the percentage of interpretable reads that showed one of two possible variant-types at the highly polymorphic regions illustrated in (B) – rs35084281 and rs34057178. Parental H9 is the unmodified control cell line, #7 is an internal wild-type control clone generated following nucleofection of KLF17(3_1), #1, #8, #9, 10 and #11 are the KLF17-targeted H9 clones that appeared to have undergone homozygous editing based on short-range genotyping. **(D-F)** Illustration of the sequence context surrounding the KLF17(3_1) cut site in (D) the wild-type reference sequence, (E) the case of an 8 bp deletion and (F) the case of a 1 bp insertion. Important features of the KLF17 sequence are highlighted. DNA sequence is shown in regular font, amino acid sequence is bold above or below the DNA.

Development • Supplementary information

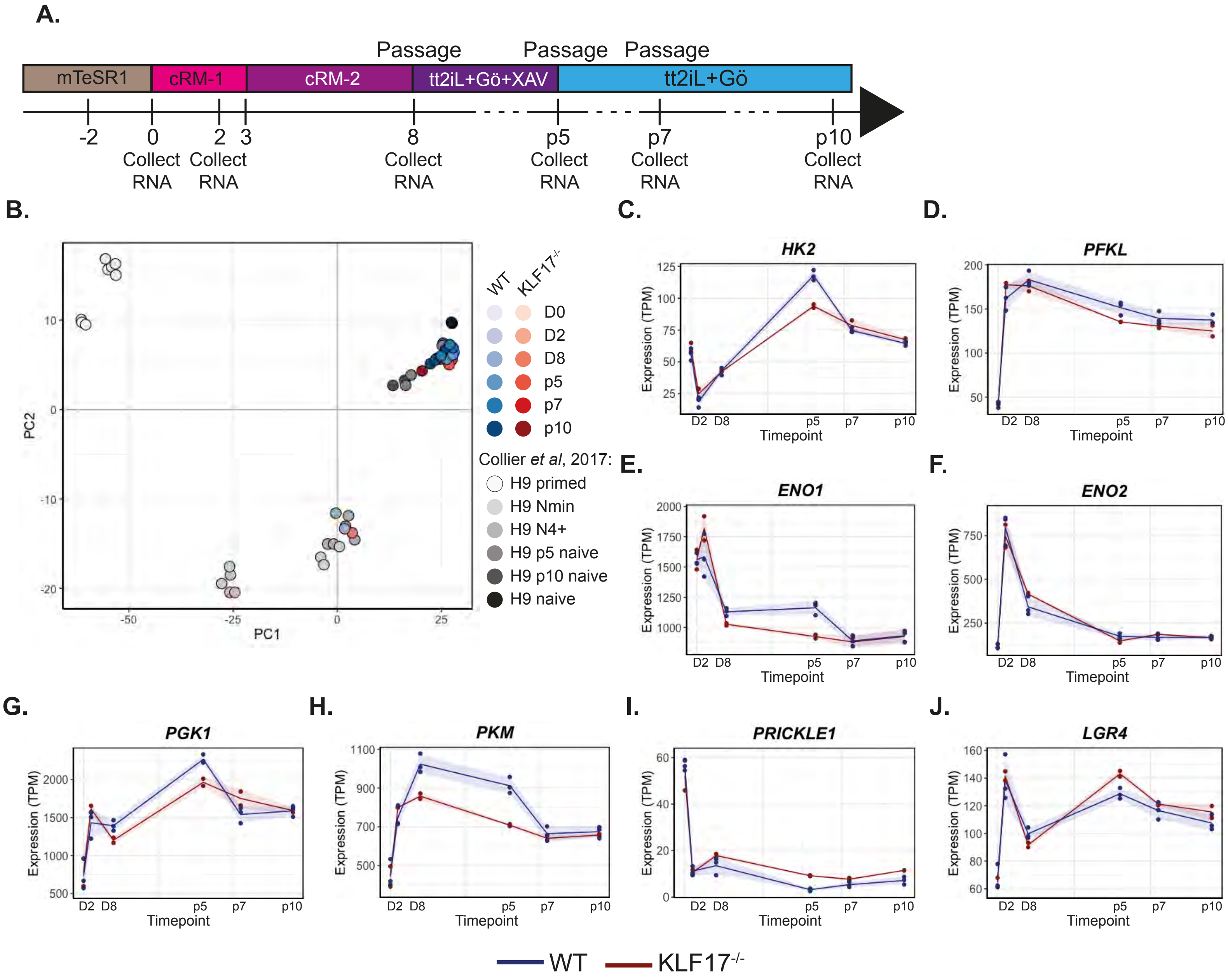

Fig. S11. ***KLF17*-null naïve hESCs at passage 5 display misregulated expression of core glycolytic enzymes and WNT pathway components. (A)** Schematic representation of the chemical epigenetic resetting experiment, showing the timings of mRNA collections. **(B)** Batch-corrected PCA analysis of the bulk RNA-seq data shown in Fig. 6B incorporated with data from *NK2*-driven resetting (Collier *et al*., 2017). **(C-J)** Normalised expression (TPM) of individual genes of interest across the resetting protocol showing (C-H) downregulation of glycolytic enzymes and (I-J) upregulation of WNT signalling factors at p5. Solid lines show the mean value and shading shows the mean ± s.d.

Development • Supplementary information

Table S1.- Genes significantly differentially expressed following 5 days expression of ectopic *KLF17* in primed hESCs

Click here to download Table S1

Table S2. - Genes whose expression is highly correlated (r ≥ 0.85) to ectopic *KLF17*

Click here to download Table S2

Table S3. - Genes associated with WNT signalling and significantly downregulated following 24hrs ectopic *KLF17* expression in primed hESCs

Click here to download Table S3

Table S4. - Genes significantly differentially expressed following 5 days expression of ectopic *KLF17* in hESCs cultured in PXGL medium

Click here to download Table S4

Table S5. - Genes significantly differentially expressed following 5 passages in naive conditions of *KLF17*$^{-/-}$ hESCs

Click here to download Table S5

Table S6. - Genes associated with WNT signalling and significantly upregulated following 5 passages in naive conditions of *KLF17*$^{-/-}$ hESCs

Click here to download Table S6

Table S7. Primary and secondary antibodies used in immunofluorescence.

Target	Species	Dilution	Supplier	Catalogue Number
**Anti-DNMT3L**	Mouse	1 in 500	Abcam	ab93613
**Anti-DPPA5**	Rabbit	1 in 250	Sigma Aldrich	D2569
**Anti-GP130**	Rabbit	1 in 250	Thermo Fisher	PA5-80735
**Anti-HA (3F10)**	Rat	1 in 500	Sigma Aldrich (Roche)	11867423001
**Anti-KLF5**	Rabbit	1 in 500	Abcam	ab137676
**Anti-KLF17**	Rabbit	1 in 500 (hESCs)<br>1 in 200 (embryo)	Atlas Antibodies	HPA024629
**Anti-NANOG**	Goat	1 in 200	R&D Systems	AF1997
**Anti-OCT4**	Mouse	1 in 100	Santa Cruz Biotechnology	SC-5279
**Anti-SOX2**	Rat	1 in 100	Invitrogen	14-9811-82
**Anti-SUSD2**	Mouse	1 in 250	Biolegend	327401
**Anti-TFAP2C**	Goat	1 in 200	R&D Systems	AF5059
**Anti-VENTX**	Rabbit	1 in 500	Cambridge Bioscience	HPA050955
**Alexa Fluor anti-mouse IgG**	Donkey	1 in 300	Invitrogen	A21202 (488 nm)<br>A21203 (594 nm)<br>A31571 (647 nm)
**Alexa Fluor anti-rabbit IgG**	Donkey	1 in 300	Invitrogen	A21206 (488 nm)<br>A21207 (594 nm)<br>A31573 (647 nm)
**Alexa Fluor anti-goat IgG**	Donkey	1 in 300	Invitrogen	A11055 (488 nm)<br>A11058 (594 nm)<br>A21447 (647 nm)
**Alexa Fluor anti-rat IgG**	Donkey	1 in 300	Invitrogen	A21208 (488 nm)<br>A21209 (594 nm)

Development • Supplementary information

Table S8. Primers used for qRT-PCR.

Target	Forward primer sequence	Reverse primer sequence
ARGFX	CCAGTTTCACTCTGTTATCCAAG	CGTTCTTTATGCCTTCTCCG
DNMT3L	GGACCCTTCGATCTTGTGTA	ACCAGATTGTCCACGAACAT
DPPA5	GTGGTTTACGGCTCCTATTT	TCATCCAAGGGCCTAGTT
GAPDH	GATGACATCAAGAAGGTGGTG	GTCTACATGGCAACTGTGAGG
KLF17	ACCCAGTCTTCATGTACGGC	GCACTCCAGAGCTTCCAGAA
KLF17_HA	ACACCAGAAGACTCATCGGC	ACATCGTATGGGTAAGGACCAG
NANOG	CATGAGTGTGGATCCAGCTTG	CCTGAATAAGCAGATCCATGG
ZFP42	GGAATGTGGGAAAGCGTTCGT	CCGTGTGGATGCGCACGT
TFCP2L1	AGCACATCCACCGAGTCTAC	TGAGGACAAAACAGGATTCATCT
VENTX	CAGCTCTCAGAGGTCCAGATA	AGACGTTGAGTAGAAAGCTGG

Table S9. Primers used for genotyping the KLF17 on-target locus.

Target	Forward primer sequence	Reverse primer sequence
**Exon 1**	GTGGCGATGTACCGATACCC	CTCCGCCTCACCTCTCCT
**Exon 3**	GGACCTTCCCTTTTGAATCCTC	CGGCTGCACTGATCACATTT
**Long-range**	ACAGGTGAAGGAGGTGTCAG	GAACTGGTCAGAGGCAGGTA

Development • Supplementary information

Table S10. Primary and secondary antibodies used in western blot

Target	Species	Dilution	Supplier	Catalogue Number
**Anti-alpha tubulin**	Mouse	1 in 1000	Sigma Aldrich	T9026
**Anti-pan AKT**	Mouse	1 in 2000	Cell Signaling Technologies	2920
**Anti-phospho AKT (Ser473)**	Rabbit	1 in 2000	Cell Signaling Technologies	4060
**Anti-phospho AKT (Thr308)**	Rabbit	1 in 1000	Cell Signaling Technologies	13038
**Anti-pan ERK1/2**	Mouse	1 in 2000	Cell Signaling Technologies	9107
**Anti-phospho ERK1/2**	Rabbit	1 in 2000	Cell Signaling Technologies	4370
**Anti-pan IGF1R**	Rabbit	1 in 1000	Cell Signaling Technologies	3027
**Anti-pan Insulin Receptor (InsR)**	Mouse	1 in 1000	Cell Signaling Technologies	3020
**Anti-phospho IGF1R/InsR**	Rabbit	1 in 1000	Cell Signaling Technologies	3024
**Anti-KLF17**	Rabbit	1 in 500	Atlas Antibodies	HPA024629
**Anti-pan S6**	Mouse	1 in 1000	Cell Signaling Technologies	2317
**Anti-phospho S6**	Rabbit	1 in 1000	Cell Signaling Technologies	2211
**Anti-mouse IgG (H+L), HRP-conjugated**	Goat	1 in 20000	Cell Signaling Technologies	7076
**Anti-rabbit IgG (H+L), HRP-conjugated**	Goat	1 in 20000	Cell Signaling Technologies	7074
**Anti-goat IgG (H+L), HRP-conjugated**	Donkey	1 in 20,000	Santa Cruz Biotechnology	SC-2020
**Anti-rat IgG (H+L), HRP-conjugated**	Goat	1 in 20,000	Cell Signaling Technologies	7077

Development • Supplementary information
