# Supplementary material for: KLF17 promotes human naïve pluripotency but is not required for its establishment
Source: Development. 2021 Nov 15;148(22):dev199378. doi: 10.1242/dev.199378 (PMC8645209; doi:10.1242/dev.199378)
Supplement: Reviewer comments [file develop-148-199378_review_history.pdf]

KLF17 promotes human naïve pluripotency but is not required for its establishment

Rebecca Anne Lea, Afshan McCarthy, Stefan Boeing, Todd Fallesen, Kay Elder, Phil Snell, Leila Christie, Sarah Adkins, Valerie Shaikly, Mohamed Taranissi and Kathy K Niakan
DOI: 10.1242/dev.199378

Editor: Patrick Tam

Review timeline

Original submission:	18 December 2020
Editorial decision:	26 January 2021
First revision received:	14 September 2021
Accepted:	11 October 2021

Original submission

First decision letter

MS ID#: DEVELOP/2020/199378

MS TITLE: KLF17 promotes human naive pluripotency but is not required for its establishment

AUTHORS: Rebecca Anne Lea, Afshan McCarthy, Stefan Boeing, and Kathy K Niakan

I have now received all the referees' reports on the above manuscript, and have reached a decision. The referees' comments are appended below, or you can access them online: please go to BenchPress and click on the 'Manuscripts with Decisions' queue in the Author Area.

As you will see, the referees express considerable interest in your work, but have some significant criticisms and recommend a revision of your manuscript before we can consider publication. If you are able to revise the manuscript along the lines suggested, which may involve further experiments (e.g., on genetically modified hESCs), I will be happy receive a revised version of the manuscript. Your revised paper will be re-reviewed by one or more of the original referees, and acceptance of your manuscript will depend on your addressing satisfactorily the reviewers' major concerns. Please also note that Development will normally permit only one round of revision.

We are aware that you may be experiencing disruption to the normal running of your lab that make experimental revisions challenging. If it would be helpful, we encourage you to contact us to discuss your revision in greater detail. Please send us a point-by-point response indicating where you are able to address concerns raised (either experimentally or by changes to the text) and where you will not be able to do so within the normal timeframe of a revision. We will then provide further guidance. Please also note that we are happy to extend revision timeframes as necessary.

Please attend to all of the reviewers' comments and ensure that you clearly highlight all changes made in the revised manuscript. Please avoid using 'Tracked changes' in Word files as these are lost in PDF conversion. I should be grateful if you would also provide a point-by-point response detailing how you have dealt with the points raised by the reviewers in the 'Response to Reviewers' box. If you do not agree with any of their criticisms or suggestions please explain clearly why this is so.

© 2021. Published by The Company of Biologists under the terms of the Creative Commons Attribution License (https://creativecommons.org/licenses/by/4.0/).

Reviewer 1

Advance summary and potential significance to field

Prior studies have uncovered KLF17 as a marker of human naïve pluripotency. In this paper, the authors demonstrate the overexpression of KLF17 in primed hESCs (and under primed hESC culture conditions) induces a more naïve pluripotency-like expression program. The authors then speculate that KLF17 overexpression might enhance the conversion of primed hESCs to the naïve state. Indeed they find that chemical resetting medium enables efficient reprogramming upon KLF17 overexpression. Yet, they also show that s null mutation of KLF17 in conventional hESCs does not interfere with the naïve resetting or the maintenance of resulting naïve hESCs. Overall, the insights into the regulation of human naïve pluripotency are relatively small, yet, importantly, this paper nicely addresses the role of a key marker of the naïve pluripotent state. The obvious follow-up experiments include reprogramming in alternative naïve media and similar experiments with the Klf5/17 double knockout. The latter is interesting as the authors demonstrate that Klf5 becomes induced upon Klf17 deletion. Regardless, Klf17 seems like an interesting transcription factor that is regulated differently between mouse and human and therefore, this work is helpful for the field.

Comments for the author

Major points:
1. Whilst it appears there are global transcriptional changes when comparing UI and +Dox treated cells (Fig3A), it is unclear how much KLF17 induction can push the cell fate towards naïve pluripotency. Can the authors please expand upon the PCA analysis performed in Fig3A by including published datasets that permit this comparison, such as early and late naïve reprogramming samples from Liu 2017/Liu 2020/Collier 2017, or Linneberg-Agerholm 2019/Guo 2017 for established naïve hESCs cultured in PXGL/t2iLGö. Likewise, it would be informative to display the magnitude of expression changes between UI and +Dox treated samples relative to their expression levels in primed and naïve hESCs for Fig3C-E and S3 A-P. For example, a separate heatmap of TPM values would be sufficient.

2. The authors suggest that KLF17 may be regulating members of the WNT and PI3K-AKT-mTOR signaling pathways based upon the observation that KLF17 overexpression in primed hESCs led to a rapid downregulation of WNT components and upregulation of PI3K-AKT-mTOR components. Can the authors validate this observation by performing ChIP-seq/CUT&Run/CUT&Tag for either endogenous KLF17 or HA-tagged KLF17 to see whether KLF17 directly regulates these components? Similarly it might be interesting to uncover the binding targets of Klf17 in naïve hESCs.

3. The authors demonstrate by RNA-sequencing that WT and KLF17 null hESCs are transcriptionally similar along the time course of primed to naïve reprogramming (Fig6B). Can the authors please include published RNA-seq datasets for established naïve hESC cultures and/or intermediate reprogramming samples as mentioned in point 1 to validate successful reprogramming.

4. The authors claim that KLF17 null naïve hESCs are not as phenotypically stable as WT naïve hESCs (lines 389-392), based upon the observation that at p5 there are several hundred DEGs enriched in metabolic and signaling pathways. The data does not strongly support this conclusion given that globally the transcriptional profiles are highly similar Fig6B, and no phenotypic experiments have been performed to examine glycolysis rates or alterations in WNT signaling activity. Slight transcriptional changes at one time point are not sufficient to support this conclusion, especially given the trend no longer exists at a later passage. Can the authors please reconsider the tone of their conclusions or provide additional supporting data to justify them.

Minor points:
1. Citations for introduction lines 64-72.
2. It would be informative to label genes of interest on the volcano plots in Fig3B and S2B-C.
3. Missing citation line 250-251 Liu 2017 showed that transgene induction of KLF4 can induce naïve pluripotency.

© 2021. Published by The Company of Biologists under the terms of the Creative Commons Attribution License (https://creativecommons.org/licenses/by/4.0/).

4. Can the authors please comment on why DNMT3L staining appears to be nuclear localized, as expected in Fig4A, but in Fig4C the staining appears to be both nuclear and cytoplasmic.
5. Can the authors please either show the data for the comments made on lines 263-266 or omit these statements.
6. Can the authors please clarify Fig6 and FigS8A-F to be consistent with showing days or passage points during the reprogramming time course. This would aid the reader to know what day passage 5 relates to.
7. Figure 3E – X-axis label should be improved.

Reviewer 2

Advance summary and potential significance to field

KLF17 is an intriguing transcriptional factor that is expressed in human pre-implantation epiblast and naïve human pluripotent stem cells (PSCs), but not in the mouse equivalents. Nevertheless, the exact role of KLF17 in human is unknown. This manuscript by Dr Niakan and colleagues describes new understanding of role of KLF17 in human PSCs. First, they found that the expression dynamics of KLF17 differs from that of two other pluripotent genes, SOX2 and NANOG. Notably, they found KLF17 target pathways by qPCR and RNAseq.
The authors also showed the function of KLF17 by overexpression and knockout. Interestingly, the overexpression of KLF17 induced primed PCSs to naïve PSCs under tt2iL+Go and PXGL without KLF2/NANOG or valproic acid, which are normally required to induce naïve PSCs. However, KLF17 KO primed PSCs could be reset to naïve PSCs. The authors explained that KLF5 compensated for the role of KLF17. These findings are very interesting, in that they provide new insights on how KLF17 regulates pluripotency in human cells.

Comments for the author

There are a few questions that need answering to strengthen the study's conclusions.
1) Although I expect redundancy in the KLF family, the authors need more data validate their claim that KLF5 functionally compensated the role of KLF17. KLF17 and KLF5 double KO PSCs will be required.
2) It is difficult to distinguish the direct effect of KLF17 or the result of different cell states.
The downregulation of pPKA was analyzed at day 5 (Fig S3). Are all the data of biological replicates at day 5? What happens at day 1 or shorter time point after the induction?
3) Figure 3 Why are the gene expression profiles so different between cells under no treatment (UI)? I understood these cells were only cultured under mTeSR1.
In addition, the gene expression profiles in cells under UI and +Dox shifted to the same PC1 direction (Fig 3A). Fig 3C also showed some genes have similar time courses in UI and +Dox. Do these results reflect KLF17?

Reviewer 3

Advance summary and potential significance to field

This manuscript from Lea et al. investigates the necessity and sufficiency of the transcription factor KLF17 during induction of naïve pluripotency in human embryonic stem cells (hESCs). Previous work from the Niakan group based on single cell RNA sequencing showed that KLF17 is specifically associated with the human pre-implantation epiblast (EPI) (Blakeley et al., 2015). Here, Lea et al. monitored the dynamics of KLF17 expression in human pre-implantation embryos at the protein level, revealing a gradual restriction to the EPI lineage. The authors then turn their attention to naïve hESCs, in which KLF17 is known to be transcriptionally activated compared to conventional "primed" hESCs. Using a lentiviral overexpression strategy, they show that KLF17 activates the expression of naïve-associated transcripts and alters the expression of signaling effectors, resulting in increased expression of PI3K-AKT components and reduced expression of genes associated with WNT signaling. They also show that KLF17 overexpression drives the conversion of primed hESCs to naïve pluripotency in combination with PXGL conditions and that

© 2021. Published by The Company of Biologists under the terms of the Creative Commons Attribution License (https://creativecommons.org/licenses/by/4.0/).

primed hESCs with mutations in KLF17 are still able to undergo chemical conversion to naïve pluripotency, suggesting redundancy by other KLF paralogs.

Overall, this is a carefully designed and well-written manuscript, which clarifies that KLF17 overexpression is sufficient to induce naïve human pluripotency, but dispensable for establishment of naïve hESCs under stringent culture conditions. An obvious question for future experimental work is to test with KLF5 (or other KLF paralogs, see point 3 below) indeed compensate for the absence of KLF17 during primed-to-naïve resetting. However, such experiments would require substantial time and effort and are not needed to support the main conclusions in this manuscript. However, I do request that the authors address the points below in their revision.

Comments for the author

Specific comments:
1. Lines 73-74: “In mouse embryonic stem cells (mESCs), the triple knockout of Klf2, Klf4 and Klf5 can be rescued by human KLF17, but not mouse KLF17 (Yamane et al., Development, 2018)”. I’m not sure how the authors reached this conclusion from the Yamane paper, which showed little significant difference in rescue ability between mouse and human KLF17. Yamane et al. described their own data as follows: “The rescue ability of human KLF17 was comparable to that of mouse Klf17 and lower than that of human KLF4.”

2. The RNA-seq data indicated rapid and strong upregulation of various components of the PI3K-AKT-mTOR pathway upon KLF17 overexpression in primed hESC (Fig. 3E). Therefore, it is rather surprising to see reduction in p-AKT-Ser473 upon KLF17 overexpression (Fig. S3Q), although this may reflect negative feedback as the authors suggest. Have they checked the phosphorylation status upon KLF17 overexpression of other PI3K pathway components that they showed are activated in naïve and primed cells, e.g. AKT-T308 and S6 (pS6) (Wamaitha et al., 2020)?

3. KLF5 was upregulated in KLF17-/- cells undergoing chemical resetting (Fig. 6D), leading the authors to speculate that KLF5 may be compensating for KLF17.
However, KLF2 and KLF4 are upregulated during the early stages of primed-to-naïve resetting as well. Therefore, the potential role of these other KLF paralogs (especially KLF4) in compensating for KLF17 cannot be easily dismissed given the well-described functional redundancy among Klf family members in mouse ESCs (Jiang et al., 2008; Yamane et al. 2018). Even if KLF5 is the only KLF paralog upregulated in KLF17-/- cells, there may enough KLF4 around to compensate for both KLF5 and KLF17. It would be safer to suggest in the Discussion that KLF17 may be compensated by one or several of its paralogs in naïve cells.

4. Along the above lines, it is worth noting that Liu et al. (Nature Methods 2017) showed that overexpression of KLF4 is sufficient for conversion of primed hPSCs into naïve hPSCs in t2iLGöY media. In contrast, Lea et al. report here that KLF17 was unable to convert primed cells to naïve pluripotency in tt2iLGo (a modified version of t2iLGo). This would imply that KLF4, but not KLF17, is capable of inducing naïve pluripotency in the absence of an external WNT inhibitor.

First revision

<u>Author response to reviewers' comments</u>

Reviewer 1:
Prior studies have uncovered KLF17 as a marker of human naïve pluripotency. In this paper, the authors demonstrate the overexpression of KLF17 in primed hESCs (and under primed hESC culture conditions) induces a more naïve pluripotency-like expression program. The authors then speculate that KLF17 overexpression might enhance the conversion of primed hESCs to the naïve state. Indeed they find that chemical resetting medium enables efficient reprogramming upon KLF17 overexpression. Yet, they also show that a null mutation of KLF17 in conventional hESCs does not interfere with the naïve resetting or the maintenance of resulting naïve hESCs. Overall, the insights into the regulation of human naïve pluripotency are relatively small, yet, importantly, this paper

© 2021. Published by The Company of Biologists under the terms of the Creative Commons Attribution License (https://creativecommons.org/licenses/by/4.0/).

nicely addresses the role of a key marker of the naïve pluripotent state. The obvious follow-up experiments include reprogramming in alternative naïve media and similar experiments with the Klf5/17 double knockout. The latter is interesting as the authors demonstrate that Klf5 becomes induced upon Klf17 deletion. Regardless, Klf17 seems like an interesting transcription factor that is regulated differently between mouse and human and therefore, this work is helpful for the field.

We thank the reviewer for their kind comments and suggestions of future avenues of research to follow up.

Reviewer 1 Comments for the Author:
Major points:

1. Whilst it appears there are global transcriptional changes when comparing UI and +Dox treated cells (Fig3A), it is unclear how much KLF17 induction can push the cell fate towards naïve pluripotency. Can the authors please expand upon the PCA analysis performed in Fig3A by including published datasets that permit this comparison, such as early and late naïve reprogramming samples from Liu 2017/Liu 2020/Collier 2017, or Linneberg-Agerholm2019/Guo 2017 for established naïve hESCs cultured in PXGL/t2iLGö.

Likewise, it would be informative to display the magnitude of expression changes between UI and +Dox treated samples relative to their expression levels in primed and naïve hESCs for Fig3C-E and S3 A-P. For example, a separate heatmap of TPM values would be sufficient.

We thank the reviewers for these helpful suggestions. As suggested by the reviewer, we have incorporated the data from Collier *et al*, 2017 into our analysis pipeline. As shown below, we have found that primed hESCs induced to express KLF17 for 1 or 2 days cluster closely with the day 10 resetting intermediates from the published study (H9 Nmin and H9N4+). We have included this analysis in our revised manuscript in Fig. 3E, as shown below.

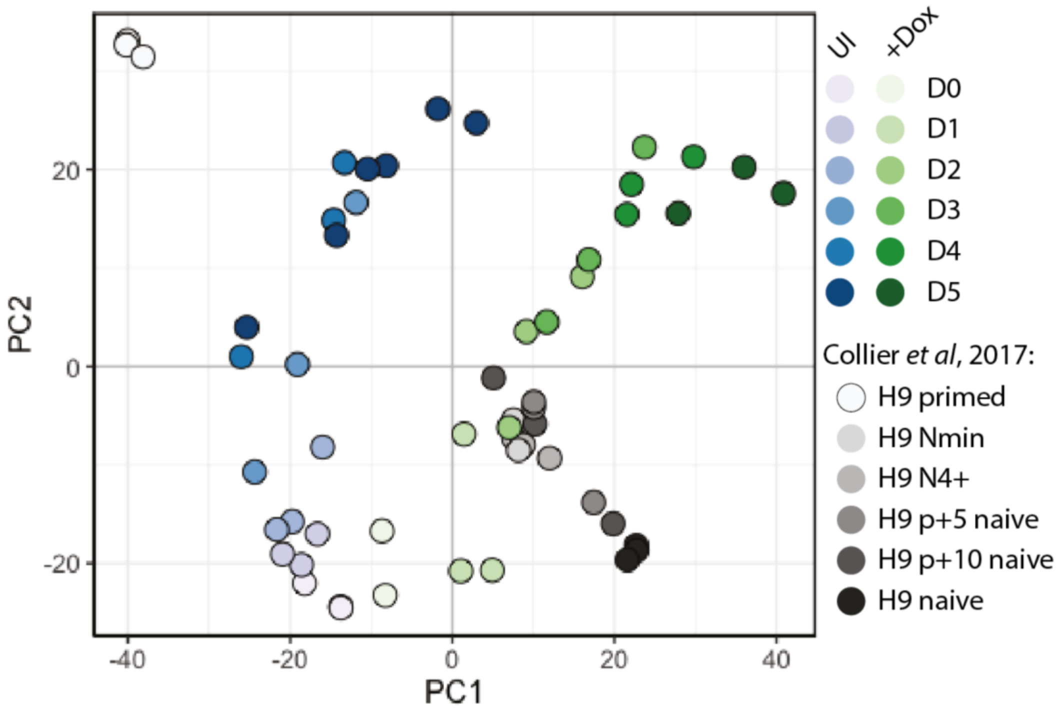

Additionally, we have generated the requested heatmap to allow a comparison of the magnitude of expression changes of particular genes of interest in uninduced and induced H9 KLF17-HA hESCs and previously published naïve and primed hESCs which is included in Fig. S4Q, as shown below.

© 2021. Published by The Company of Biologists under the terms of the Creative Commons Attribution License (https://creativecommons.org/licenses/by/4.0/).

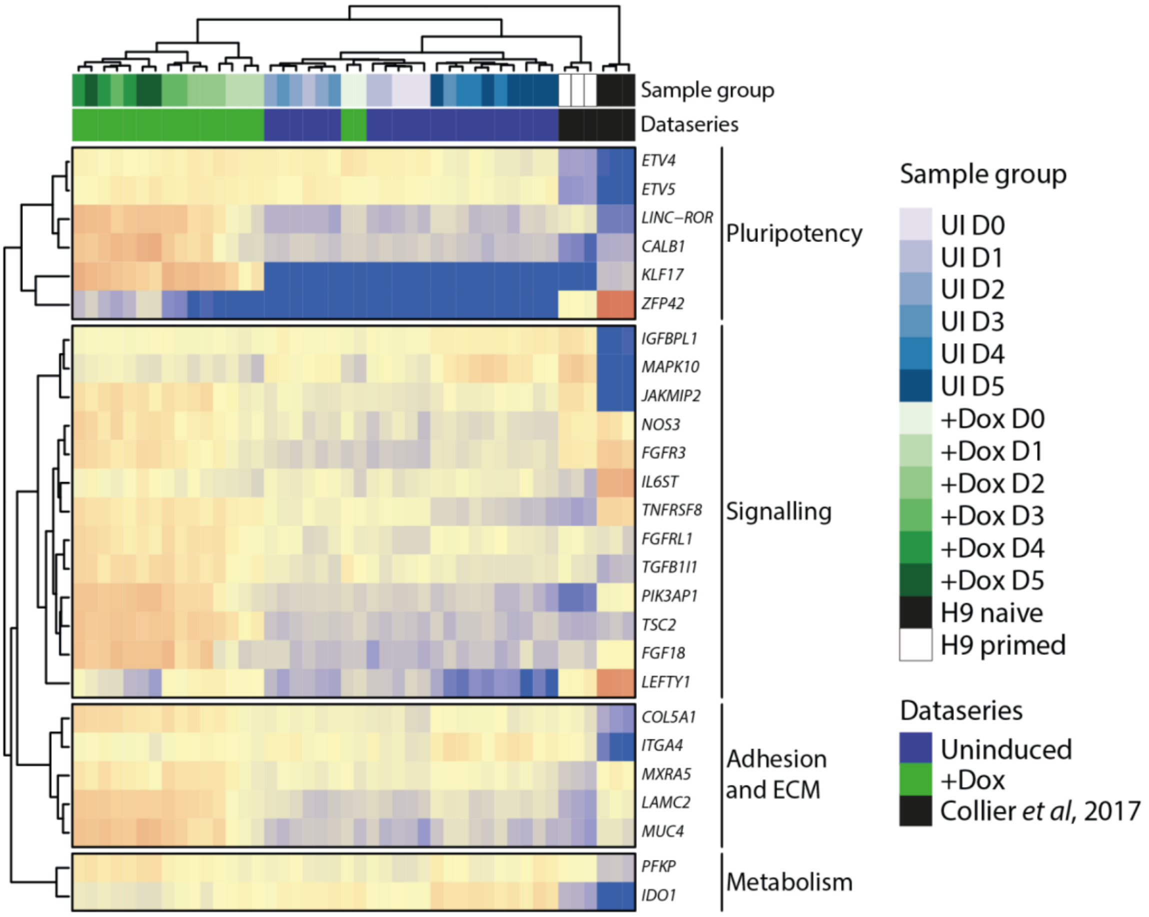

2. The authors suggest that KLF17 may be regulating members of the WNT and PI3K-AKT- mTOR signaling pathways based upon the observation that KLF17 overexpression in primed hESCs led to a rapid downregulation of WNT components and upregulation of PI3K-AKT- mTOR components. Can the authors validate this observation by performing ChIP- seq/CUT&Run/CUT&Tag for either endogenous KLF17 or HA-tagged KLF17 to see whether KLF17 directly regulates these components? Similarly it might be interesting to uncover the binding targets of Klf17 in naïve hESCs.

We agree with the reviewer that occupancy analysis would be very informative in this case. We have tried to perform ChIP-seq for KLF17 in the induced cells multiple times, using either an anti-HA antibody or an anti-KLF17 antibody. We and our external collaborators atthe Babraham Institute have found that unfortunately this does not work well and requires further optimisation. Instead, we have mined published ChIP-seq data for KLF17 occupancy in naïve hESCs established under alternative naïve conditions (HENSM medium, Bayerl *et al*, 2020). Theresults of this analysis are included in lines 242-248 and Fig. S6A-E.

3. The authors demonstrate by RNA-sequencing that WT and KLF17 null hESCs are transcriptionally similar along the time course of primed to naïve reprogramming (Fig6B). Can the authors please include published RNA-seq datasets for established naïve hESC cultures and/or intermediate reprogramming samples as mentioned in point 1 to validate successful reprogramming.

This is also an incredibly informative and important comparison and we thank the reviewer for suggesting it. As above, we have integrated the time course data from Collier *et al*, 2017 and present this data in Fig. S11B. We find that the clustering between the samples in this study and those previously published is very good, indicating successful resetting of both wild-type and *KLF17*$^{-/-}$ hESCs in this work.

© 2021. Published by The Company of Biologists under the terms of the Creative Commons Attribution License (https://creativecommons.org/licenses/by/4.0/).

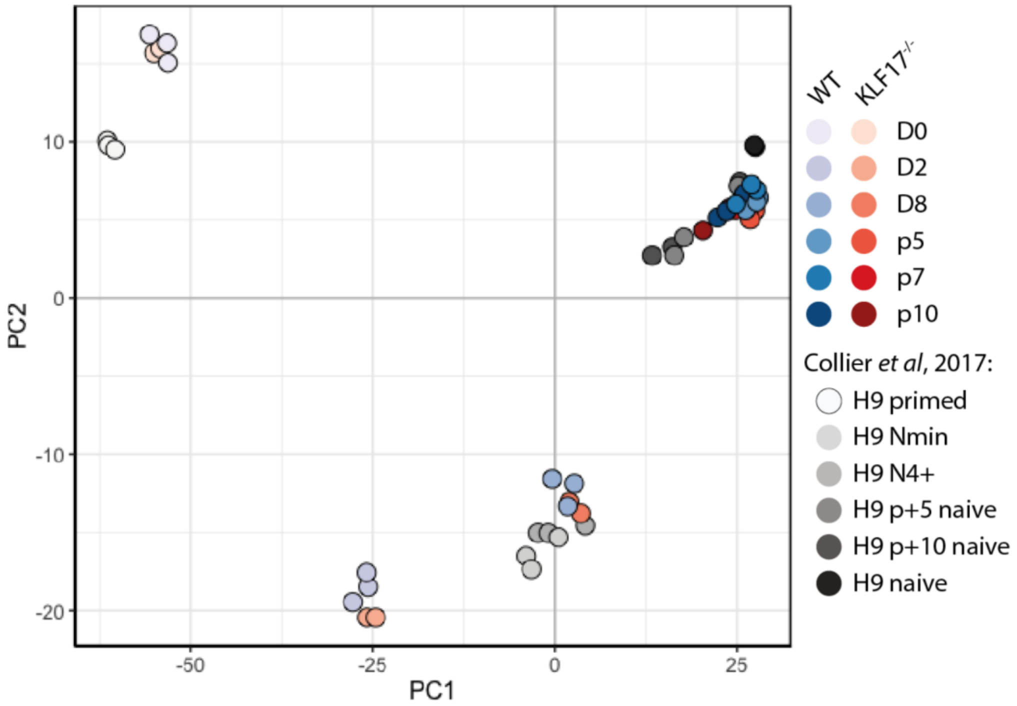

4. The authors claim that KLF17 null naïve hESCs are not as phenotypically stable as WT naïve hESCs (lines 389-392), based upon the observation that at p5 there are several hundred DEGs enriched in metabolic and signaling pathways. The data does not strongly support this conclusion given that globally the transcriptional profiles are highly similar Fig6B, and no phenotypic experiments have been performed to examine glycolysis rates oralterations in WNT signaling activity. Slight transcriptional changes at one time point are not sufficient to support this conclusion, especially given the trend no longer exists at a laterpassage. Can the authors please reconsider the tone of their conclusions or provide additional supporting data to justify them.

We appreciate the reviewer's comments and have heavily modified the results (lines 397-413) and discussion sections (lines 488-499) to ensure we are not overinterpreting the findings from RNA-seq analysis.

Minor points:

1. Citations for introduction lines 64-72.

2. It would be informative to label genes of interest on the volcano plots in Fig3B and S2B-C.

3. Missing citation line 250-251 Liu 2017 showed that transgene induction of KLF4 can induce naïve pluripotency.

We have updated lines 64-72 and 257-262 with the appropriate citations and made therequested changes to these figures.

4. Can the authors please comment on why DNMT3L staining appears to be nuclear localized, as expected, in Fig4A, but in Fig4C the staining appears to be both nuclear andcytoplasmic.

Across all panels in Fig. 4, DNMT3L staining is both nuclear and cytoplasmic. This is unusual,as we did not detect this pattern of staining in response to ectopic *KLF17* induction under primed conditions (Fig. S6). However, the levels of DNMT3L expression are much greater under the naïve conditions (PXGL and/or tt2iL+Gö) shown in Fig. 4. We suspect that the dramatic increase in the level of DNMT3L protein has led to a degree of non-specific localisation.
In particular, in Fig. 4C, the three-dimensional nature of the naïve hESC colonies makes the nuclear localisation slightly more difficult to detect when there is a background of cytoplasmic expression. We have updated the results section, lines 286-287 to acknowledge this difference in staining.

© 2021. Published by The Company of Biologists under the terms of the Creative Commons Attribution License (https://creativecommons.org/licenses/by/4.0/).

5. Can the authors please either show the data for the comments made on lines 263-266 or omit these statements.

We have added additional brightfield images (Fig. S7B) to demonstrate this data andadjusted the comments in lines 269-280.

6. Can the authors please clarify Fig6 and FigS8A-F to be consistent with showing days or passage points during the reprogramming time course. This would aid the reader to know what day passage 5 relates to.

7. Figure 3E – X-axis label should be improved.

We have made the requested changes to the labelling of these figures and thank thereviewer for their very helpful comments.

Reviewer 2:
KLF17 is an intriguing transcriptional factor that is expressed in human pre-implantation epiblast and naïve human pluripotent stem cells (PSCs), but not in the mouse equivalents. Nevertheless, the exact role of KLF17 in human is unknown. This manuscript by Dr Niakan and colleagues describes new understanding of role of KLF17 in human PSCs. First, they found that the expression dynamics of KLF17 differs from that of two other pluripotent genes, SOX2 and NANOG. Notably, they found KLF17 target pathways by qPCR and RNAseq.The authors also showed the function of KLF17 by overexpression and knockout.
Interestingly, the overexpression of KLF17 induced primed PCSs to naïve PSCs under tt2iL+Go and PXGL without KLF2/NANOG or valproic acid, which are normally required to induce naïve PSCs. However, KLF17 KO primed PSCs could be reset to naïve PSCs. The authors explained that KLF5 compensated for the role of KLF17. These findings are very interesting, in that they provide new insights on how KLF17 regulates pluripotency in humancells.

We are grateful to the reviewer for their very kind comments on our work.

Reviewer 2 Comments for the Author:
There are a few questions that need answering to strengthen the study's conclusions.
1) Although I expect redundancy in the KLF family, the authors need more data validate their claim that KLF5 functionally compensated the role of KLF17. KLF17 and KLF5 double KOPSCs will be required.

We appreciate that our current data is only suggestive of a possible compensatory role of KLF5 upregulation during naïve hESC establishment. As requested, we have made progress toward generating the required genetically modified hESCs.

In particular, we have generated independent clonal *KLF5*-mutant hESC lines by CRISPR- Cas9-mediated genome editing in a wild-type background. These lines each harbour compound mutations that lead to the introduction of premature termination codons (PTCs)within the zinc finger-encoding regions.

For the last 4 months, we have also been attempting to generate *KLF5*/*KLF17* double null mutant lines, by using the same CRISPR-Cas9 targeting approach in our existing *KLF17*$^{-/-}$ primed hESCs. As shown in the graph below, in comparison to the relatively high efficiencyof compound null lines generated in the wild-type background (*KLF5*), targeting in the *KLF17*$^{-/-}$ background is proving difficult (*KLF17*/*KLF5*). So far, screening 106 clones has yielded only a single line harbouring two independent frameshift alleles. While we have been able to identify four clones carrying a single frameshift allele, we are very reluctant tocarry forward such lines based on our previous experience in targeting *KLF17*, as described in lines 355-370 It is likely that the clones with only one identifiable indel harbour a large deletion or translocation event on the other allele that would be impossible to capture by standard genotyping. In order to characterise these lines and determine if they are suitablefor assessing the specific function of a dual *KLF17*/*KLF5* knockout, we would need to perform karyotyping by low-pass whole-genome sequencing and an in-depth "walk-along" approach to genotyping.

© 2021. Published by The Company of Biologists under the terms of the Creative Commons Attribution License (https://creativecommons.org/licenses/by/4.0/).

Therefore, our preference would be to carry forward clearly genotyped compound mutants from more than one clonal line as generated already for *KLF17* and *KLF5* single targeting.
However, the incredibly low rate of incidence (<1%) suggests that we would need to generate and genotype >200 additional double-targeted hESC lines in order to identify three independent clones that could be used in phenotypic experiments.

We anticipate that performing this additional CRISPR-Cas9-mediated targeting, clonal derivation, genotyping and phenotypic characterisation could take upwards of 12 months to do well. We would therefore like to ask the reviewers to kindly consider our current conclusions on the role of KLF17 without the additional experimental data from *KLF17*/*KLF5*double-null hESC lines.

We have removed unpublished data.

2) It is difficult to distinguish the direct effect of KLF17 or the result of different cell states. The downregulation of pPKA was analyzed at day 5 (Fig S3). Are all the data of biological replicates at day 5? What happens at day 1 or shorter time point after the induction?

We agree with the reviewer that it is difficult to understand the dynamic nature of changes in signalling pathways by investigating only the day 5 time point.

We have now updated our western blot analysis to include uninduced and induced samples at Days 1, 2 and 5, as well as further targets in the signalling pathway. We have updated this section in the manuscript on lines 204-217 and included an updated Fig. S5A.

© 2021. Published by The Company of Biologists under the terms of the Creative Commons Attribution License (https://creativecommons.org/licenses/by/4.0/).

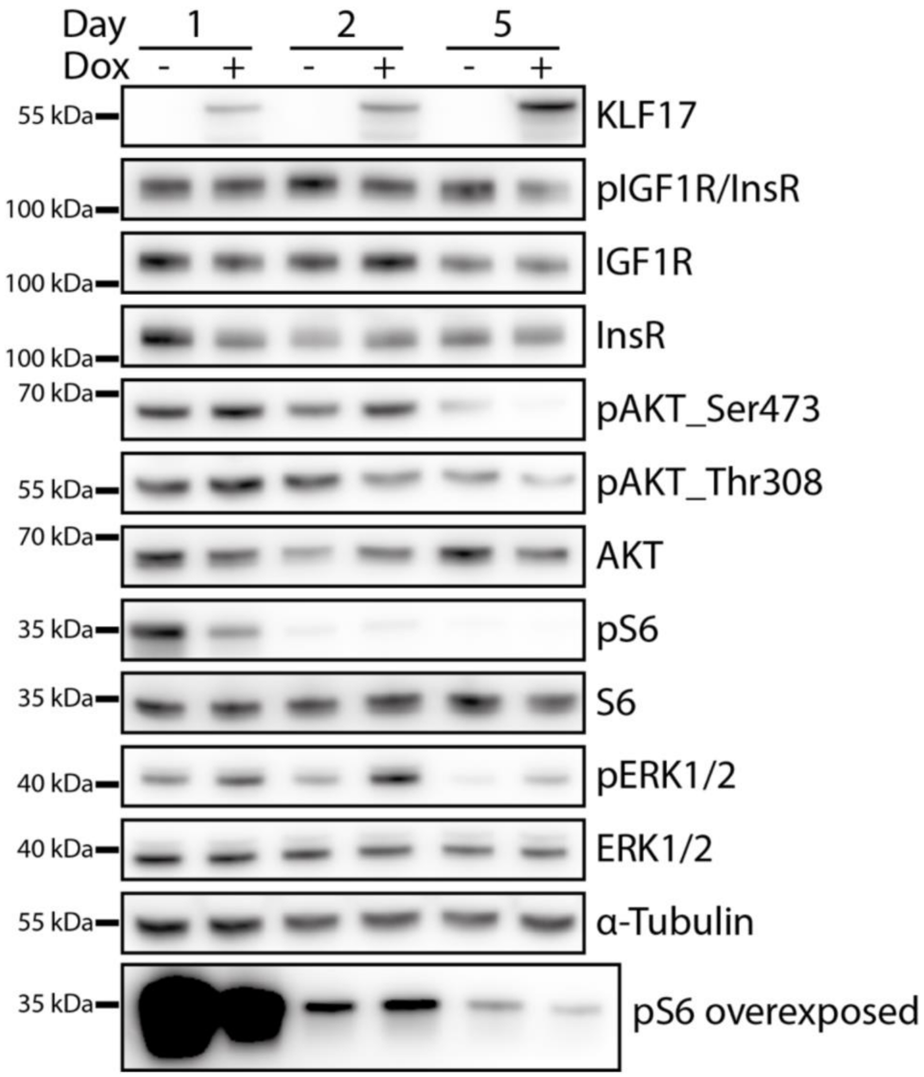

3) Figure 3 Why are the gene expression profiles so different between cells under no treatment (UI)? I understood these cells were only cultured under mTeSR1.
In addition, the gene expression profiles in cells under UI and +Dox shifted to the same PC1 direction (Fig 3A). Fig 3C also showed some genes have similar time courses in UI and +Dox. Do these results reflect KLF17?

We apologise for the confusion. The shift of UI cells in the PCA (Fig. 3A) is not reflective of *KLF17* expression, as our RNA-seq analysis shows no significant expression of *KLF17* in the absence of Dox induction (Fig. 3C). Rather, some of the variability represented in PC1 recapitulates the time of hESCs in culture following a passage. Even the uninduced hESCs are adapting between day 0 (1 day after plating following a passage) and day 5 (6 days afterplating), regardless of KLF17 expression status. This is a standard variability of hESCs and wehave clarified this point in the results section, lines 165-172.

Reviewer 3:
This manuscript from Lea et al. investigates the necessity and sufficiency of the transcription factor KLF17 during induction of naïve pluripotency in humanembryonic stem cells (hESCs). Previous work from the Niakan group based on single cell RNA sequencing showed that KLF17 is specifically associated with the human pre-implantation epiblast (EPI) (Blakeley et al., 2015). Here, Lea et al. monitored the dynamics of KLF17 expression in human pre-implantation embryos at the protein level, revealing a gradual restriction to the EPI lineage. The authors then turn their attention to naïve hESCs, in which KLF17 is known to be transcriptionally activated compared to conventional “primed” hESCs. Using a lentiviral overexpression strategy, they show that KLF17 activates the expression of naïve-associated transcripts and alters the expression of signaling

© 2021. Published by The Company of Biologists under the terms of the Creative Commons Attribution License (https://creativecommons.org/licenses/by/4.0/).

effectors, resulting in increased expression of PI3K-AKT components and reduced expression of genes associated with WNT signaling. They also showthat KLF17 overexpression drives the conversion of primed hESCs to naïve pluripotency in combination with PXGL conditions and that primed hESCs with mutations in KLF17 are still able to undergo chemical conversion to naïve pluripotency, suggesting redundancy by other KLF paralogs.

Overall, this is a carefully designed and well-written manuscript, which clarifies that KLF17 overexpression is sufficient to induce naïve human pluripotency, but dispensable for establishment of naïve hESCs under stringent culture conditions. An obvious question for future experimental work is to test with KLF5 (or other KLF paralogs, see point 3 below) indeed compensate for the absence of KLF17 during primed-to-naïve resetting. However, such experiments would require substantial time and effort and are not needed to support the main conclusions in this manuscript. However, I do request that the authors address the points below in their revision.

We thank the reviewer for their very kind comments on our manuscript and constructive feedback.

Reviewer 3 Comments for the Author:
Specific comments:

1. Lines 73-74: "In mouse embryonic stem cells (mESCs), the triple knockout of Klf2, Klf4 and Klf5 can be rescued by human KLF17, but not mouse KLF17 (Yamane et al., Development, 2018)". I'm not sure how the authors reached this conclusion from the Yamane paper, which showed little significant difference in rescue ability between mouse and human KLF17. Yamane et al. described their owndata as follows: "The rescue ability of human KLF17 was comparable to that of mouse Klf17 and lower than that of human KLF4."

We are grateful to the reviewer for pointing out this error and we have amended the text appropriately (lines 73-75).

2. The RNA-seq data indicated rapid and strong upregulation of various components of the PI3K-AKT-mTOR pathway upon KLF17 overexpression in primed hESC(Fig. 3E). Therefore, it is rather surprising to see reduction in p-AKT-Ser473 upon KLF17 overexpression (Fig. S3Q), although this may reflect negative feedback as the authors suggest. Have they checked the phosphorylation status upon KLF17 overexpression of other PI3K pathway components that they showed are activated in naïve and primed cells, e.g. AKT-T308 and S6 (pS6) (Wamaitha et al., 2020)?

This is an important point also raised by Reviewer 2. As stated above, we have now expanded our analysis of the PI3K-AKT signalling pathway to include a wider range of important pathway components, as well as earlier timepoints post induction of KLF17 expression. This has reinforced the notion of reduced signalling activity. These results are now included in the manuscript, lines 204-217 and as Supplementary Figure 5A.

3. KLF5 was upregulated in KLF17-/- cells undergoing chemical resetting (Fig. 6D), leading the authors to speculate that KLF5 may be compensating for KLF17.However, KLF2 and KLF4 are upregulated during the early stages of primed-to- naïve resetting as well. Therefore, the potential role of these other KLF paralogs (especially KLF4) in compensating for KLF17 cannot be easily dismissed given the well-described functional redundancy among Klf family members in mouseESCs (Jiang et al., 2008; Yamane et al. 2018). Even if KLF5 is the only KLF paralog upregulated in KLF17-/- cells, there may enough KLF4 around to compensate for both KLF5 and KLF17. It would be safer to suggest in the Discussion that KLF17 may be compensated by one or several of its paralogs in naïve cells.

We thank the reviewer for bringing this important point to our attention and we have updated the discussion to clearly state the possibility of compensation by KLF4 and/or KLF2 in lines 453-462 and 476-486.
We believe that there is some evidence in the literature for a for a potential overlap in function between KLF5 and KLF17 and we have updated the manuscript on lines 464-474 toexpand on this in the Discussion section.

© 2021. Published by The Company of Biologists under the terms of the Creative Commons Attribution License (https://creativecommons.org/licenses/by/4.0/).

4. Along the above lines, it is worth noting that Liu et al. (Nature Methods, 2017) showed that overexpression of KLF4 is sufficient for conversion of primedhPSCs into naïve hPSCs in t2iLGöY media. In contrast, Lea et al. report here that KLF17 was unable to convert primed cells to naïve pluripotency in tt2iLGo(a modified version of t2iLGo). This would imply that KLF4, but not KLF17, is capable of inducing naïve pluripotency in the absence of an external WNT inhibitor.

We thank the reviewer for reminding us of this relevant experiment by Liu *et al*, 2017. We have updated our Discussion section (lines 441-451) to discuss this experiment and how thisrelates to our results.

Second decision letter

MS ID#: DEVELOP/2020/199378

MS TITLE: KLF17 promotes human naive pluripotency but is not required for its establishment

AUTHORS: Rebecca Anne Lea, Afshan McCarthy, Stefan Boeing, Todd Fallesen, Kay Elder, Phil Snell, Leila Christie, Sarah Adkins, Valerie Shaikly, Mohamed Taranissi, and Kathy K Niakan
ARTICLE TYPE: Research Article

Thanks for submitting a revised version of the manuscript. The outcome of the re-view is positive. I am happy to tell you that your manuscript has been accepted for publication in Development, pending our standard ethics checks.

I would, however, suggest considering adding a note to the paper (see Editor's note), which you may be able to do so when the page proof is available for your approval.

Reviewer 1

Advance summary and potential significance to field

Prior studies have uncovered KLF17 as a marker of human naïve pluripotency. In this paper, the authors demonstrate the overexpression of KLF17 in primed hESCs (and under primed hESC culture conditions) induces a more naïve pluripotency-like expression program. The authors then speculate that KLF17 overexpression might enhance the conversion of primed hESCs to the naïve state. Indeed they find that chemical resetting medium enables efficient reprogramming upon KLF17 overexpression. Yet, they also show that s null mutation of KLF17 in conventional hESCs does not interfere with the naïve resetting or the maintenance of resulting naïve hESCs. Klf17 is an interesting transcription factor that is regulated differently between mouse and human. This study suggests that other Klf factors collaborate with Klf17 to promote naive pluripotency.

Comments for the author

The authors have satisfactorily addressed most of my earlier concerns. I now recommend this interesting study for publication in DEVELOPMENT.

Reviewer 2

Advance summary and potential significance to field

This is a review of a revised manuscript, thus the results will not be resummarised.

Comments for the author

The authors have made full revision to addres my comments. I have no more suggestions.

© 2021. Published by The Company of Biologists under the terms of the Creative Commons Attribution License (https://creativecommons.org/licenses/by/4.0/).

<u>Reviewer 3</u>

Advance summary and potential significance to field

Lea et al. have provided a detailed response to the reviewers' questions integrating additional transcriptome data into their figures and updating their western analysis of the AKT signaling pathway. They attempted to disrupt KLF5 in the KLF17-/- background but were only able to generate a single line harboring two independent frameshift alleles. I agree with the authors that additional clearly genotyped compound mutant lines would be needed to reach any firm conclusions regarding compensation of KLF17 by KLF5. However, such experiments are beyond the scope of the present study and not essential to support the main conclusions of this paper. Overall, this revised study carefully explores the role of KLF17 in human naïve pluripotency and will be of interest to the readers of Development.

Comments for the author

Since the authors were unable to perform KLF17 ChIP-seq, they incorporated ChIP data from Bayerl et al. 2021 into their analysis. It remains unclear, however whether the naïve-like cells in that study are in fact in a comparable developmental state as the naive cells used by Lea et al. I therefore advise the authors to be cautious when interpreting those ChIP data.

© 2021. Published by The Company of Biologists under the terms of the Creative Commons Attribution License (https://creativecommons.org/licenses/by/4.0/).
